# On the colour of wing scales in butterflies: iridescence and preferred orientation of single gyroid photonic crystals

**DOI:** 10.1098/rsfs.2016.0154

**Published:** 2017-06-16

**Authors:** Robert W. Corkery, Eric C. Tyrode

**Affiliations:** 1 Department of Chemistry, School of Chemical Science and Engineering, KTH Royal Institute of Technology, 100 44 Stockholm, Sweden; 2 Department of Applied Mathematics, Research School of Physics and Engineering, The Australian National University, Canberra, ACT 2601, Australia

**Keywords:** butterfly, gyroid, nanostructure, biophotonic

## Abstract

*Lycaenid* butterflies from the genera *Callophrys*, *Cyanophrys* and *Thecla* have evolved remarkable biophotonic gyroid nanostructures within their wing scales that have only recently been replicated by nanoscale additive manufacturing. These nanostructures selectively reflect parts of the visible spectrum to give their characteristic non-iridescent, matte-green appearance, despite a distinct blue–green–yellow iridescence predicted for individual crystals from theory. It has been hypothesized that the organism must achieve its uniform appearance by growing crystals with some restrictions on the possible distribution of orientations, yet preferential orientation observed in *Callophrys rubi* confirms that this distribution need not be uniform. By analysing scanning electron microscope and optical images of 912 crystals in three wing scales, we find no preference for their rotational alignment in the plane of the scales. However, crystal orientation normal to the scale was highly correlated to their colour at low (conical) angles of view and illumination. This correlation enabled the use of optical images, each containing up to 10^4^–10^5^ crystals, for concluding the preferential alignment seen along the 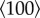 at the level of single scales, appears ubiquitous. By contrast, 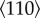 orientations were found to occur at no greater rate than that expected by chance. Above a critical cone angle, all crystals reflected bright green light indicating the dominant light scattering is due to the predicted band gap along the 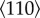 direction, independent of the domain orientation. Together with the natural variation in scale and wing shapes, we can readily understand the detailed mechanism of uniform colour production and iridescence suppression in these butterflies. It appears that the combination of preferential alignment normal to the wing scale, and uniform distribution within the plane is a near optimal solution for homogenizing the angular distribution of the 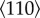 band gap relative to the wings. Finally, the distributions of orientations, shapes, sizes and degree of order of crystals within single scales provide useful insights for understanding the mechanisms at play in the formation of these biophotonic nanostructures.

## 1. Introduction

Photonic crystals interact with light to produce structural colour in a way that is fundamentally distinct from dyes and pigments. Photonic crystals contain a repeating motif of alternating materials with high and low optical dielectric permitivities, with a periodicity in the range of the wavelength of visible light. Given an appropriate engineering design or set of evolutionary selection agents, photonic band gaps, as calculated by Maxwell's equations, can arise in these periodic dielectric materials whereby certain frequencies of light are forbidden to propagate through the structure, leading to a selective reflection of these colours.

Photonic crystals are responsible for spectacular colour displays of plants and animals such as the iridescent blues of *Pollia* fruits and *Begonia* leaves [1], the multicoloured metallic iridescence of peacock tail feathers [2] and tropical butterflies [3], the dynamic and adaptive colour camouflage and displays of chameleons [4] and squids [5] as well as the blue skin of Mandrill monkeys [6]. Photonic crystals have also been found in fossilized organisms, from the elytra of ancient weevils [7] to the iridescent feathers of birds [8] and dinosaurs [9] and in other life forms including viruses [10] and bacteria [11].

Photonic crystals occurring in plants and animals have a characteristic iridescence, whereby their colour can change depending upon the angle of the viewer and/or the light illuminating it. Rapid motion of iridescent feathers can thereby lead to a mesmerizing flashing of colours that can have a positive effect in sexual selection or for confusing predators [12]. In some organisms, it has been clearly advantageous to suppress iridescence by employing a mosaic of variously oriented microscopic photonic crystals to achieve an overall averaged colour with little angular dependence.

**Figure 1.**
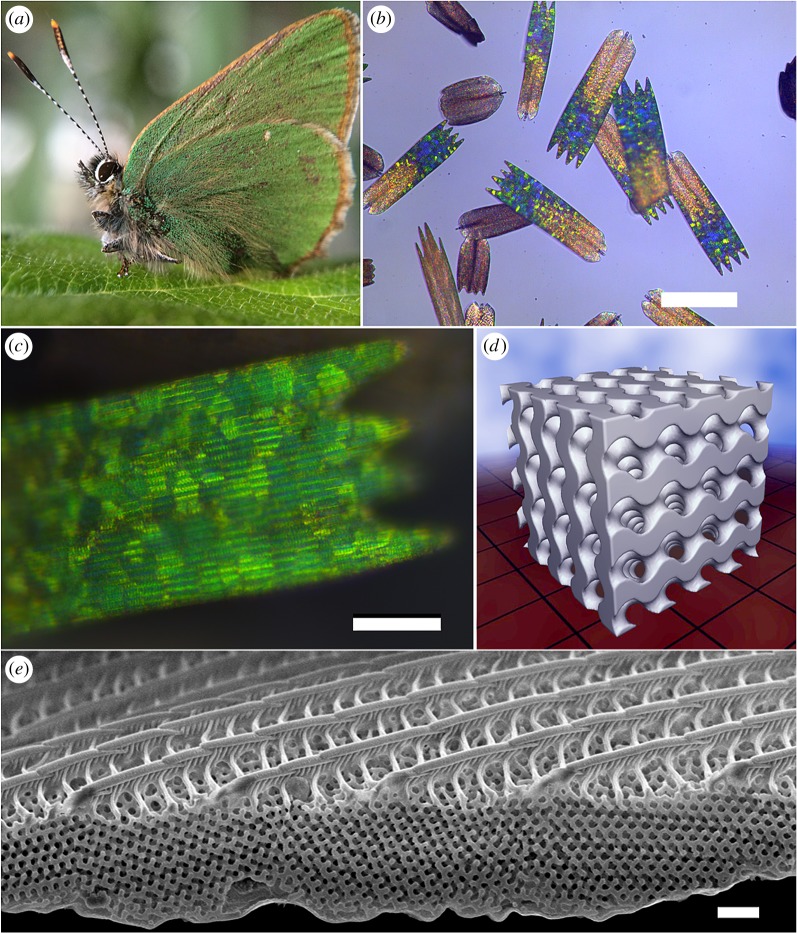
(*a*) *Callophrys rubi* (Linnaeus, 1758) also known as the green hairstreak; (*b*) optical microscope image of both coloured and brown cover scales from *C. rubi* taken at low NA; (*c*) high NA (high magnification) optical micrograph of a coloured wing scale showing that the characteristic green–yellow colour arises from many individual photonic crystal domains. Note that the longitudinal ribs are also visible; (*d*) computer-generated single gyroid model—4 × 4×4 unit cells; (*e*) SEM image of a coloured wing scale showing the ribbed upper surface, an undulating lower plate and five porous, single gyroid domains in different orientations. Note the continuous network spanning crystal grain boundaries suggestive of twinning planes, yet with some lattice defects such as dislocation planes and holes. Scale bars: (*b*, *c* and *e*) 100, 20 and 1 µm, respectively.

*Callophrys rubi* (the green hairstreak; Lepidoptera: Lycaenidae) is a common and widespread Nearctic lycaenid butterfly species from Europe. This butterfly, and other European and North American species of the same genera, are thought to use this method of iridescence suppression mentioned above to produce a foliage-matched [13,14], matte-green camouflage coating (figure 1*a*). The wings of these butterflies and some species of the related genera *Cyanophrys* and a few other species, notably *Teinopalpus imperialis* and *Parides sesostris*, contain thousands of variously oriented, three-dimensional single gyroid PCs to achieve a uniform green colour [13–24]. The single gyroid structure has crystallographic space group I4_1_32, a subgroup of Ia3d to which the original gyroid minimal surface discovered by Schoen [25] belongs. The gyroid minimal surface partitions space into two mutually interpenetrating labyrinths, each described as a branched tunnel network, where the medial axis of each tunnel system is enantiomorphic to the other. Formally, these networks are known as ‘srs’ or Y* nets [26]. Single gyroid photonic crystals in butterflies are thus chiral structures whose handedness depends on which tunnel network is in-filled.

The relative coverage of butterfly wing scales by photonic crystals with certain orientations is important for understanding the formation of these structures and for modelling their colour. In *P. sesostris* and *T. imperialis*, the relative balance of crystal orientations is known [23,24]—with each species displaying a single crystallographic axis parallel to the surface normal of the scales. Further, it is also important to observe the photonic response of single gyroids in each orientation to elucidate the quality of the crystals and to understand their optical properties. Experimental evidence directly correlating the photonic response of single gyroids crystals with their orientation is relatively scarce at visible wavelengths, for butterflies or for engineered materials [27,28]. Most evidence comes from the direct correlation of model crystal structures with their corresponding simulated responses [14,19,21,29–31].

Here, we report a microscopic structural analysis of the crystal orientations in three *C. rubi* wing scales. We find a strong preferential alignment of certain crystal axes normal to the planes of the respective wing scales. This preferential alignment is then correlated to the observed angular-dependent photonic response at the level of single domains. This correlation is then used to map crystal orientational preference over whole wings of *C. rubi,* and other butterflies species using optical imaging alone. The photonic response and formation of single gyroids are then discussed, as well as some broader implications for the overall non-iridescent colour of *C. rubi*.

## 2. Experimental set-up

### 2.1. Butterfly and scale preparation

*Callophyrs rubi* specimens were collected in an endemic pine forest within the Royal National City Park Stockholm. No permit was required for their collection. Specimens of *P. sesostris* (NHRS-JLKB00025254), *T. imperialis* (NHRS-JLKB00025255), *Cyanophrys acaste* (NHRS-JLKB00025236), *C. dumetorum* (NHRS-JLKB00025240) and *Chalybs hassan* (NHRS-JLKB00025242) were loans from the Department of Entomology, Swedish Museum of Natural History. Scales were removed from wing scales by gentle abrasion with a scalpel or pin. Individual wing scales were mounted on glass microscope slides or double-sided carbon adhesive tape using either a micromanipulation rig (i.e. scale 1 and scale 2) or by contacting double-sided tape to scales placed on a glass slide (scale 3).

### 2.2. Scanning electron microscopy imaging and image processing

Wing scales were imaged with either a JSM-7401F or JSM-7000F scanning electron microscope (SEM) using secondary electron detection using either upper (SEI) or lower (LEI) modes. Working distance was 7.8–8.0 mm with 5.0 kV accelerating voltage (7401F in LEI mode) or 10 mm with 3.0 kV accelerating voltage (7000F in SEI mode). Samples were uncoated and imaged at magnifications up to 18 000× on both microscopes.

Mosaic images of two entire wing scales and one partial wing scale were assembled from multiple individual images, each set taken at magnifications of 3500× (scale 2) or 4000× (scale 1 and scale 3). Overlapping regions of interest and minor histogram adjustments allowed accurate alignment of individual images with minimal contrast variation. Pixel resolution was 23.25 nm pixel^−1^ for scale 1 and scale 3, and 37.625 nm pixel^−1^ for scale 2. The resultant mosaic image files contain 8113 × 2592 pixels, 4700 × 2400 pixels and 7700 × 2900 pixels, for scale 1, scale 2 and scale 3, respectively. High-resolution mosaic images of scales 1, 2 and 3 are available as additional electronic supplementary material.

Power spectra of the assembled mosaic images were obtained using a two-dimensional fast-Fourier transform (FFT) algorithm. One-dimensional FFTs were generated by integrating the intensity values in two-dimensional datasets along circles of increasing pixel radius (radial integration) from 0 to 4096 pixels. At a resolution of 5.2553 × 10^−6^ nm^−1^ pixel^−1^, the *k*-space range was from 0 to 0.021526 nm^−1^. For whole-scale power spectra, the equivalent *d*-space range (where *d* = k^−1^ nm) was approximately *d* ≥ 2000–67 nm.

### 2.3. Domain boundary mapping

The mosaic SEM images corresponding to three wing scales from two specimens of *C. rubi* were manually mapped (912 in total). Boundaries of photonic crystals were marked by inspection at points of discontinuity of symmetry or azimuth of their constituent Miller planes in real space (see electronic supplementary materials, including section S2 for additional details). Domain boundaries were placed with an estimated average accuracy of ±100–200 nm. The area of each domain was then determined by an automated edge detection and area-calculating algorithm in the software package ImageJ.

### 2.4. Assignment of individual crystal orientations

Each of the 912 domains mapped using SEM in the three wing scales examined was assigned a crystal orientation based on detailed analysis of their structures and by comparison with tilt-series images of simulated gyroids and their power spectra (FFTs). For the simulations, the well-known trigonometric approximation to the gyroid minimal surface was used to construct SEM projections with a volume fraction of 20% (see electronic supplementary material, sections S11 and S12 for details).

Crystals were classified according to alignment of the following crystal axes normal to the plane of the scale: 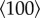, 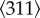, 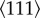, 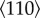 or to other classification. The assignments were made allowing for estimates of tilts away from these axes of up to approximately ±5° (classified as on-axis) or ±10° (classified as off-axis). Statistical checks of these assignments were made by comparing power spectra of polycrystalline domains in a single orientation with that of the power spectrum of the whole wing scale image when all other orientations were masked out. From this, the misidentification rate is estimated to be less than 5%. Further details plus examples are given in electronic supplementary material, sections S2 and S12 .

### 2.5. Optical microscopy and image processing

Prior to mapping the structural domains of individual wing scales, optical images of the same scales were taken in a colour-calibrated Leica microscope at a series of different magnifications/numerical apertures (NAs), using the following Leica objectives: 5×/0.12, 10×/0.25, 20×/0.4, 50×/0.75 and 100×/0.85 (note that the NA is directly related to the cone of angles of the incident light, see electronic supplementary material, section S5 for details). Images were recorded using a Leica DFC295 digital camera (3 megapixel, Micron MT9T001 progressive scan CMOS sensor fitted with a Bayer mosaic RGB filter and a 650 nm near IR low cut filter).

Image analysis was used to find the distribution of colour hues in each image and then relate these to wavelengths corresponding to the hue values obtained for narrow band colour filters of known wavelength: Edmund optics OD4 12.5 mm (FWHM 10 nm) filters centred at 440, 486, 492, 540 and 580 nm. Images to be used for colour calibration were collected by placing a colour filter directly in front of the light source, and reflecting this light to the camera from a broadband dielectric mirror (Newport, 300–1100 nm) placed in focus below the objective. Prior to placing colour filters in the light path, the digital camera was white-balanced against the white light source. Images for colour calibration were collected with the camera gamma set to 1.0 and the integration time set to the maximum value just below saturation of individual pixels in any of the RGB channels. Typical exposures were 0.5–200 ms. Eight bit RGB TIFF files were converted to an image stack comprised of 8 bit grey-scale hue, saturation and brightness channels using ImageJ software (1.49v). A 256 channel hue histogram was output to a two column file containing the number of pixels per hue channel.

Hue histograms corresponding to the different wavelength filters were fitted with Gaussians allowing hue to wavelength conversion for each filter for fixed imaging conditions. A calibration curve was then generated from the hue-wavelength data using an interpolation function in KaleidaGraph (version 4.5.2). This allowed production of pseudo-spectral plots for each individual domain as a function of NA.

### 2.6. Optical spectroscopy

Optical reflection spectra of *C. rubi* were obtained by using a modified upright Axio microscope (Zeiss, Germany) using a fibre-optic coupled to a handheld spectrometer (Spectral Products, SM440-USB). Similar to the image analysis process mentioned above, a broadband dielectric mirror (Newport, 300–1100 nm) was used for calibration. *Callophrys rubi* spectral intensities were divided by the mirror spectral intensities collected with a series of Zeiss objectives of varying NAs (each intensity first adjusted by subtracting a dark background. The list of objectives is shown in electronic supplementary material, section S5). The methodology allowed the average spectral response from multiple domains within a scale to be determined as a function of NA.

### 2.7. Band gap calculations

Band gaps were calculated using the MIT Photonics Bandgap (MPB) [32] software for predicting mid-gap frequencies and comparison with observed data. The software was run with a resolution of 16 and a mesh size of 8 for the first six bands. Higher resolution and mesh sizes slowed the calculations without adding significant information (see electronic supplementary material, section S7 for additional details).

## 3. Results

### 3.1. Analysis of the wing scale structure in *Callophrys rubi*

The wings of *C. rubi* comprise overlapping coloured cover scales and non-coloured ground scales, which are more obviously elongated near the wing extremities. The growth sequence appears to be an alternation of cover and ground scales within a single row, with subsequent rows grown over the previous ones. Upon removal of the scales from the centre of the wings, regularly spaced lines of sockets into which the foot of each scale docks can be observed and their separation distances measured (electronic supplementary material, figure S1*a*). Processing the images of these aligned socket arrays using FFT allowed the quantification of the periodic patterns. Rows of sockets are spaced apart by approximately 80 µm, and separated in each row by an average of approximately 20 µm. Therefore, the scale number density for each scale type is approximately 31 250 cm^−2^

Statistics related to various wing scale dimensions were generated by measuring more than 120 coloured wing scales of the butterfly *C. rubi* (see electronic supplementary material, section S1 for details). The average scale size is approximately 171 × 48 µm with a thickness varying from 1 to 3 µm. The highly coloured region is largely restricted to the distal half of the scale (figure 1*b*; electronic supplementary material, S1). The wing scale has a perforated upper (superior or abwing) lamina surface and non-perforated lower (inferior or adwing) lamina surface (figure 1*e*). Each scale has two to six distal protrusions (teeth) (figure 1*b*,*c*), a pair of proximal lobes and a pedicle with which it joins to the scale socket on the wing. The upper lamina is decorated by a series of closely spaced longitudinal ribs (ribs or ridges), with those at the edge (marginal stria) of the scale joining the upper and lower laminae. The spacing between ribs is approximately 2 µm. Longitudinal ribs are joined in the upper laminae by cross-ribs (transverse ridges or flutes) that run roughly orthogonal to the main ribs and are spaced about 700 nm apart (figure 1*e*). The cross-ribs appear to be outgrowths from a series of close-spaced vertical corrugations of the main ribs spaced approximately 100–200 nm apart (figure 1*e*). Between the upper and lower laminae is a matrix of fused polycrystalline gyroid domains. This matrix comprises domains that are ordered at the distal end and disordered towards the proximal end, although a clear pattern of ordered fused domains is observed closer to the proximal end along the marginal striae and along the scale centreline. Single gyroid crystals are themselves fused to and contiguous with the ribs and cross-ribs (figure 1*e*) and also appear to be only loosely attached to the lower lamina as evidenced by frequent delaminations. Lastly, holes and defects occur along crystalline domain boundaries and reduce in extent towards the distal end. Holes also occur in the domain boundaries on the lower lamina and occasionally are seen in cross-sections as defects on domain boundaries between the upper and lower laminae.

### 3.2. Crystal size, orientations and lattice parameters

A total of 912 domains were structurally mapped and analysed in three wing scales (e.g. electronic supplementary material, figures S3 and S4) yielding statistics on the distribution of domain sizes (electronic supplementary material, figure S5) and indicated the geometric relation between neighbours. The shapes were mostly distorted hexagons arranged at random. Three-way junctions between crystals were common, having approximately 120° angles resembling plateau junctions in foams, consistent with some boundary length minimization related to surface or line tension between immature crystals during growth. Some departure from this mode of boundary minimization was evidenced by the straight-line borders, often with angular and re-entrant structures consistent with facets. The observation of these features suggests that crystallization forces played a significant role in the determination of the boundary properties late in the domain formation. The appearance of one or more hole defects at the vertices of the domains was not uncommon, with adjacent holes often forming crystal boundaries (electronic supplementary material, figure S3).

High-resolution cross-sectional images (figure 1*e*; electronic supplementary material, figure S6) revealed that single domains generally traverse the entire lumen of a wing scale, with close to vertical domain boundaries. However, in some rare occurrences, the boundaries are closer to horizontal, leading to domains of different orientation sharing the same vertical section (see the middle domain of electronic supplementary material, figure S6*b*). It was also noted that most, if not all domains displayed fused margins where the solid chitin structure was continuous across the boundaries, despite the symmetry breaks. This manifests locally as either a zone of intermediate crystallographic disorder or a dislocation with no significant crystallographic disorder. A survey of these boundary types is not the subject of this current work and was not investigated in detail. A significant thickness decrease from the distal to the proximal end of the scales was observed as a typical feature in *C. rubi* (e.g. electronic supplementary material, figure S6*a*). Average wing scale thickness of the ordered areas towards the distal ends ranged from 1.5 to 3.5 µm, whereas disordered areas towards the proximal end were typically thinner than 1.5 µm (electronic supplementary material, figure S2*k*,*l*).

**Figure 2.**
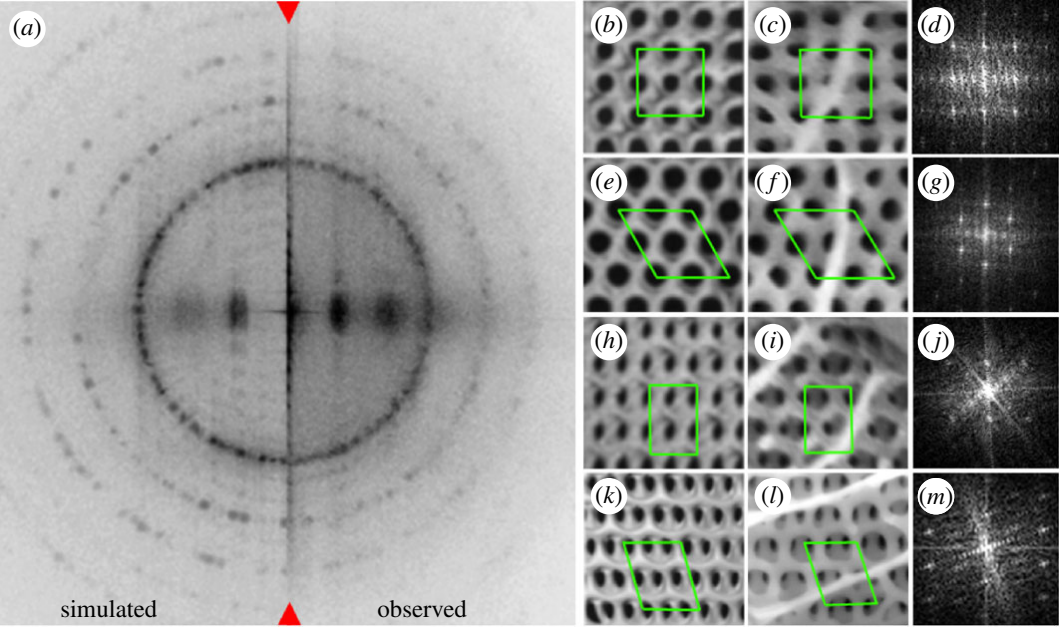
(*a*) Split two-dimensional Fourier transform of the high-resolution SEM mosaic wing scale image (left) simulated and (right) observed (also see electronic supplementary material, §3). The fact that the amplitude of each discretized ring is evenly distributed indicates that the azimuthal orientation of these planes is random. Images of the simulated (*a*, *e*, *h*, *k*) and observed single gyroid crystals in *C. rubi* (*c*, *f*, *i*, *l*) and representative FFTs of individual domains (*d*, *g*, *j*, *m*). Note that tunnels or holes that are characteristic of the single gyroid crystals are visualized as circular black features (〈100〉 (*b*, *c*) and 〈111〉 (*e*, *f*)) or elliptical (〈110〉 (*h*, *i*) and 〈311〉 (*k*, *l*)) and the solid material as white to grey. Boxes are visual aids to highlight the different geometric relation of the tunnels for each orientation. Domains images have been rotated in azimuth to align at least one set of vertical {110} planes in the horizontal. The consequence is that the cross-ribs appear then at various angles. This rotational alignment also aligns the spots in their Fourier transforms allowing them to be stacked to give an effective single-crystal pattern for each orientation which was useful in confirming the assignments of domains to a particular direction (figure 3*d*–*g*). See electronic supplementary material, sections S3 and S11 regarding the details of the projected plane lattices corresponding to the dominant observed 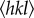 orientations. Image scale is given by the vertical height of the green boxes = 2*d*_110_ = 486 nm.

Power spectra (FFTs) from each of the high-resolution, whole-scale mosaic images yielded a series of sharp point-like and ring-like maxima in the frequency domain (figure 2*a*). From these data, the rib spacing, cross-rib spacing and single gyroid unit cell dimensions averaged over a whole scale were extracted (see later in this section).

**Figure 3.**
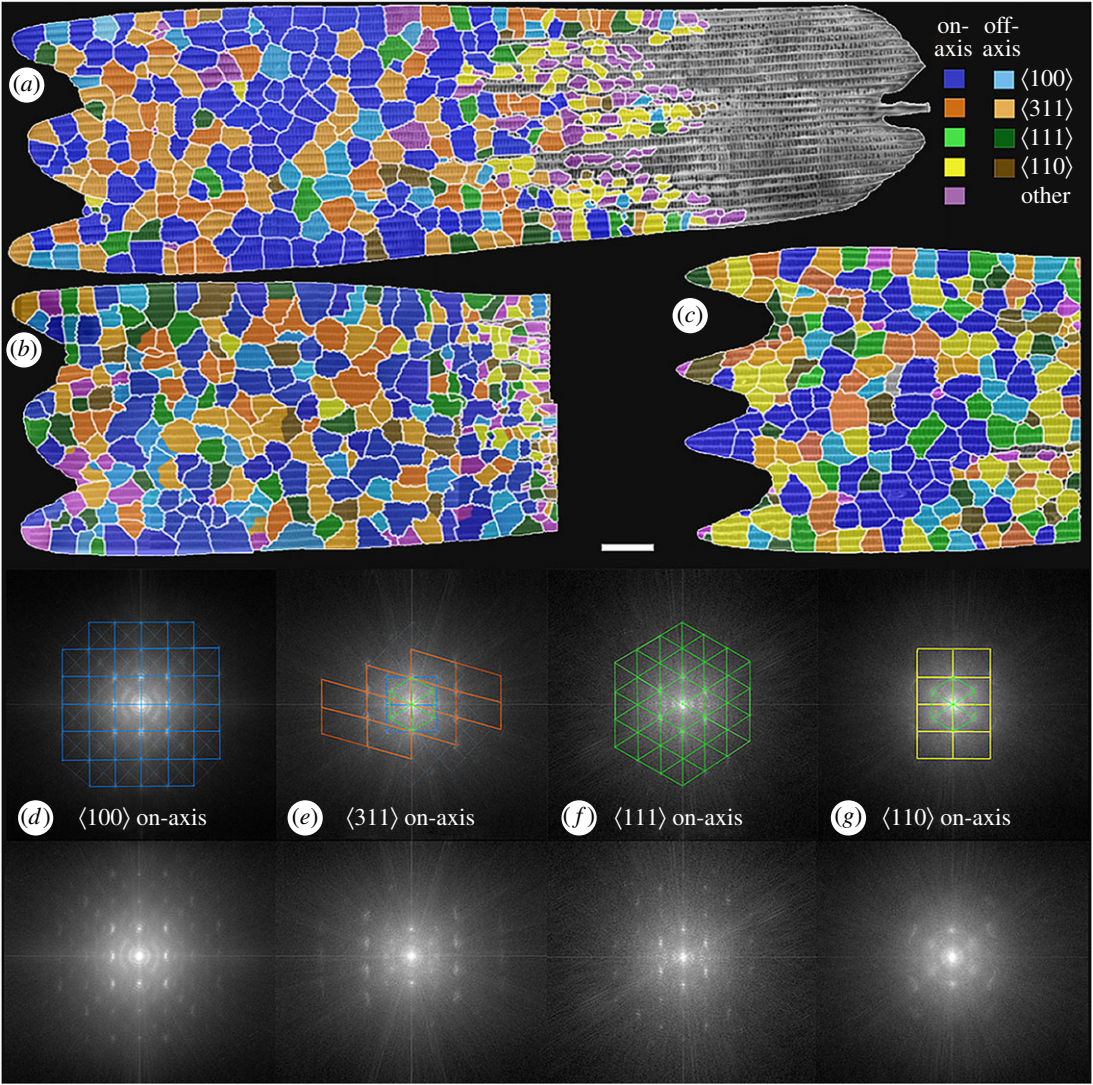
Domain maps (*a*–*c*) for three wing scales (1–3) overlaid on their corresponding SEM images. Boundaries were delineated by abrupt changes in the azimuth and/or symmetry relations of the crystallographic planes (see electronic supplementary material, section S2 and high resolution mosaic images for details). The distal end of the scale (i.e. towards the left of the images above) is rich in well-ordered crystals and so is referred to here as the ‘ordered’ region of the scale. The proximal end of the wing scales are always comprised of small, often isolated, poorly crystallized to disordered domains, with the crystalline fraction often relatively enriched in 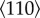 oriented domains. Cross-sections through the proximal end show the scales to be relatively thinner. Colour code: dark/light blue, on/off-axis 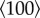; dark/bright orange, on/off-axis 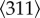; bright/dark green, on/off-axis 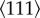; yellow/brown, on/off-axis 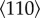; pink, other (disordered/unidentified). See electronic supplementary material, figure S12 for an enlarged view. Each panel in figure 3*d*–*g* displays the sum of all FFTs patterns taken from individual on-axis domains from wing scale 1 after azimuthal alignment (see electronic supplementary material, §12 for more details). Note that the order from left to right is the sequence in which the domains appear by rotation about a single 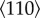 axis. The coloured grids drawn on top of the FFTs are constructed to highlight the unique symmetry displayed by domains of a particular orientation class. FFTs corresponding to the off-axis domains are given in electronic supplementary material, figure S65. Scale bar is common to all panels, (*a*–*c*) 10 µm; (*d*–*g*) 0.0077 nm^−1^.

Structural mapping at the individual domain level is shown in figure 3*a–c*. Domains fell into distinct alignments within approximately 5° or 10° of the four distinct crystal axes shown in figure 2*b*–*l*, i.e. the 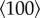, 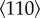, 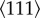 and the 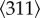 directions. In brief, domains were classified by visual inspection as being oriented along the 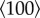 or 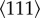 directions if they displayed 90° (square, e.g. figure 2*b*,*c*; electronic supplementary material, figure S35) or 120° (hexagonal, e.g. figure 2*e*,*f*; electronic supplementary material, figure S38) arrays of holes. Domains were classified as being oriented along the 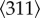 or 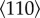 directions if they displayed approximately 74° (oblique, e.g. figure 2*k*,*l*; electronic supplementary material, figure S41) or 90° (rectangular, e.g. figure 2*h*,*i*; electronic supplementary material, figure S46) arrays of elliptical holes (see electronic supplementary material, sections S2 and S12 for a more detailed description of the mapping and assignment procedures).

As a check of the symmetry relations of each orientational class, an average FFT was generated by effectively stacking every FFT taken from individual domains within a class (figure 3*d*–*g*) (see the method described in electronic supplementary material, §S12). The effectiveness of the visual inspection method is evidenced by the fact the single-crystal Fourier transform of each orientation class is distinct (except for the off-axis 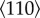) and consistent with the respective symmetry relations for that particular orientation (electronic supplementary material, section S3). Further, the differences seen in the on- and off-axis FFTs confirm that the visual inspection method is effective in distinguishing the degree of alignment of domains. Of particular importance here is that the brightest spots in every pattern are almost perfectly symmetric across both the horizontal and vertical axes indicating that the crystal classes are related to each other by rotation around a single high symmetry crystal axis. This is further supported by the fact that every pattern except that for the off-axis 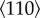 domains has only the components of its immediate neighbours as lower intensity ‘contaminants’.

**Figure 4.**
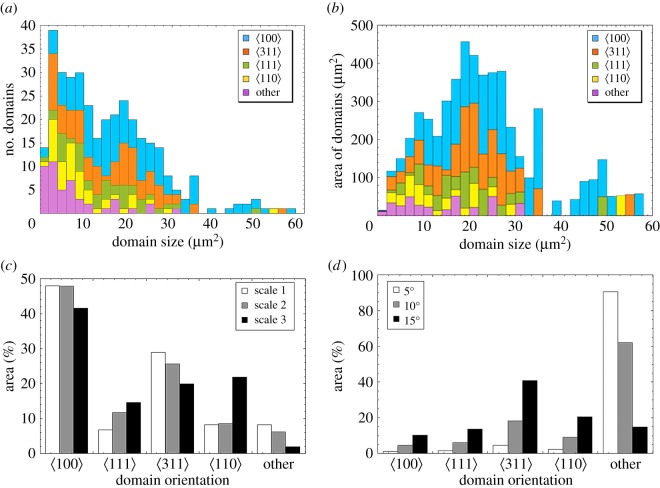
(*a–d*) Histograms of wing scale coverage by domains of different orientation for wing scale 2, showing the total number of domains versus domain size (*a*) and the total area of domains versus domain size (*b*). Statistics for scale 1 and 3 can be found in electronic supplementary material, figure S13. Bar graphs displaying the coverage by crystallites of a particular orientation are shown in (*c*). The relative probability of a particular domain having a preferred orientation within 5–15° of a particular axis by random alignment is given in (*d*). Calculation of the probabilities for preferred alignment are given in electronic supplementary material, §S4.

Domains within wing scales showed a distinct preference to align along a 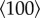 crystallographic axis. The observed coverage by 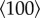 oriented domains was 40–50% (of the ordered regions) of each wing scale (figure 4*a*–*c*), and much larger than the expected coverage (less than 10%; figure 4*d*) for a random distribution. The other three observed directions were roughly as expected for a random distribution within a margin of error (figure 4*d*). Spatially, domains of similar orientation did not appear to cluster in certain parts of the scale, except for numerous smaller 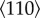 oriented domains seen in or near the disordered proximal region(s) of all three scales.

**Figure 5.**
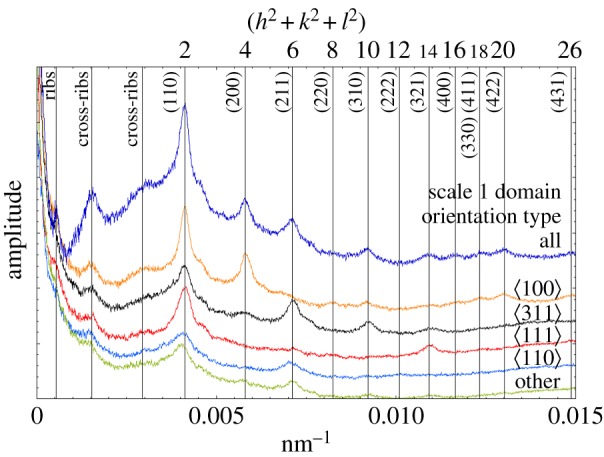
Peaks in the amplitude of the radially integrated FFTs reveal the characteristic (inverse) distances of ribs, cross-ribs and single gyroids of wing scale 1. For example, the peak at 0.0015 nm^−1^ directly gives the average distance between cross-ribs as 1/0.0015 and 667 nm. Each peak labelled (*hkl*) (e.g. (110)) identifies interplanar spacings of the single gyroid projected into two dimension (see electronic supplementary material, section S3 for additional details).

SEM mosaic images of each scale were Fourier transformed unmasked, or masked (e.g. see electronic supplementary material, figure S15), to only show the response of domains of a certain orientation (see electronic supplementary material, figure S16*a* for an example of the FFTs obtained from scale 1). Radial integration of these two-dimensional transforms yielded the one-dimensional patterns seen in figure 5. Average crystal structure parameters such as unit cell were accurately determined from these. The masked transforms confirmed the consistency of the assignments of orientation done at the individual domain level (see electronic supplementary material, sections S3 and S12).

The unit cell parameters determined from orientation-masked datasets were found to be independent of the domain orientation, with an average value for three scales of 341 ± 3 nm (see electronic supplementary material, table S2 and figure S16*b*). A unit cell value of 344 nm was used for modelling the photonic responses in §3.5.

### 3.3. Correlating image colour and crystal orientation for individual domains

**Figure 6.**
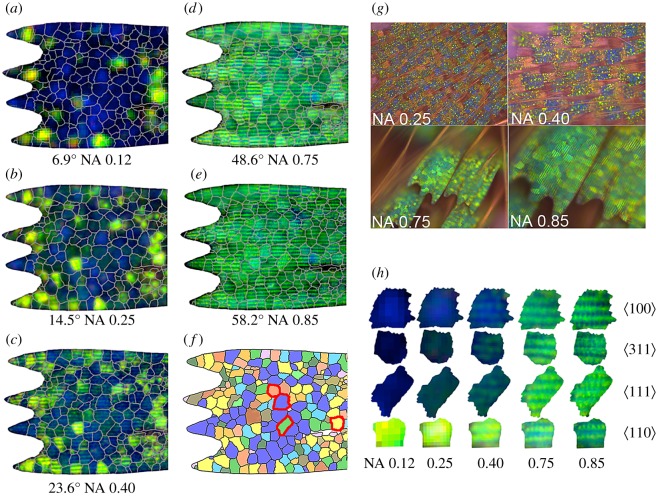
Optical microscopic images of *C. rubi* wing scales using objectives of increasing numerical aperture. Note the distinct iridescence of the scales, changing from blue/yellow at low NA to green at high NA (see electronic supplementary material, table S3 for the list of objectives used). (*a*–*e*) Scale 3 with domain boundaries from SEM mapping (*f*) overlaid with the optical images at increasing NA values and angles. Larger numbers of scales displaying the same phenomena are shown in (*g*) at different NAs. Representative domains of each orientation type highlighted with red outlines in (*f*) have been cut out from images (*a*–*e*) and appear in magnified form in (*h*) as a function of NA.

Optical imaging (figures 1*b*, 1*c* and 6) showed that at low NA, the scales contained domains that mainly scattered in the blue with minor contributions of yellow–green light, while at higher NA scattered only green light, with the transition occurring between NA 0.40 and 0.75 or from about 24° to 48° in solid incidence angle.

Overlaying these optical images (in register) on the SEM images (figure 6*a*–*e*) showed that changes in the observed optical colours were strongly correlated with both individual domains boundary locations and their respective orientation assignments. Significantly, these overlays made it possible to consistently assign a typical low-NA scattering colour to a specific domain orientation type. Brighter blue scattering correlated to 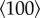, dark cyan to 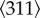 and 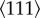, and very bright green–yellow to 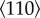 oriented domains.

Blue to green iridescence was observed with increasing NA in the light scattered from the dominant 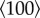 oriented domains (figure 6*a*–*e*). Using a one-dimensional analogy for this iridescent behaviour, the transition can be understood by the change from blue scattering perpendicular to the {100} crystallographic planes to the more highly scattering green–yellow perpendicular to the {110} planes within these domains. The {110} planes, which are tilted at 45° to the normal in 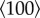 oriented domains, effectively come into view when the incident and scattered light is highly angled to the optical axis at high NA (see electronic supplementary material, figure S17). The angle at which the strongly scattering {110} planes come into view for other orientations can be gleaned from electronic supplementary material, table S4.

A similar iridescence is seen when 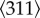 oriented domains start to scatter significantly more brightly at approximately the angle (NA) expected when the {110} planes come into view (i.e. 31.48°). Nonetheless, some weak green reflections were observed at 24°, suggesting the scattering peaks are broadened. Contributions from {100} planes could be expected to appear at 25.24° in 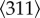 oriented domains, but no obvious blue scattering was observed, likely because the scattering in the 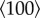 direction is far less intense than that in the 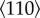 direction (see below).

Finally, the 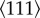 oriented domains start reflecting significantly more light just as the NA becomes large enough for the {110} planes in these domains to be reflective (i.e. greater than 35°) and again these are a green to yellow/green colour.

### 3.4. Spectral and pseudo-spectral determination of scattered colours

**Figure 7.**
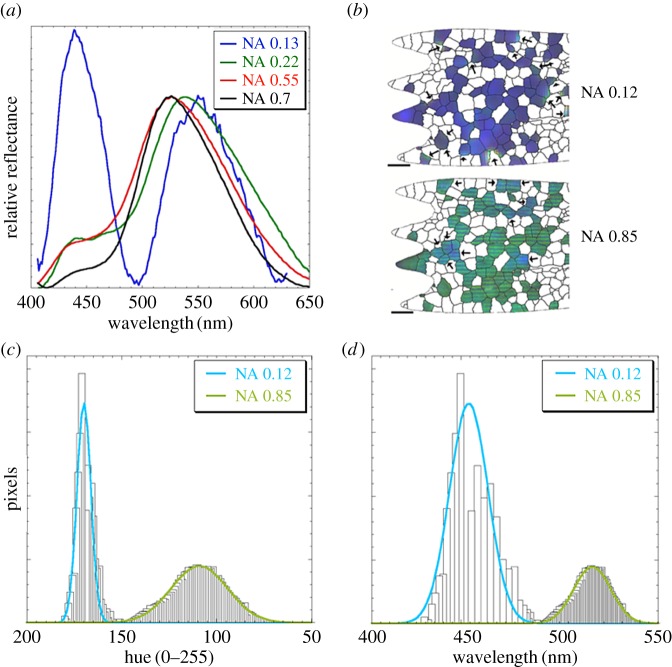
Blue–green iridescence observed in the spectra and pseudo-spectra of *C. rubi* cover wing scales. (*a*) Spectrometric curves with background subtraction and intensity normalization (at 520–550 nm) (see electronic supplementary material, figures S18 and S19 for raw curves). Note the presence of the small shoulder near 465–470 nm in the NA = 0.22 spectrum (also seen at NA = 0.13 in electronic supplementary material, figure S19). (*b*) Images illustrating the blue–green iridescence of 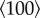 oriented domains in wing scale 3 at low and high NA. The arrows indicate outlier areas of defects in the wing scale (such as local crystal thinning) and artefacts of the imaging (e.g. blurred domain edges at low resolution). These indicated outliers were removed in constructing the histograms in (*c*) and (*d*) (also see electronic supplementary material, figures S21 and S22). The latter pseudo-spectra is in good agreement with the spectral data confirming that estimates of crystal orientation preference can be extended from single scales to large areas of butterfly wings by using imaging alone.

Spectra taken from various wing scales as a function of NA (figure 7*a*) confirm the change from blue and yellow–green dominated scattering to purely green scattering at high NA. The scattering peak near 440 nm is attributed to the photonic response from the area-dominant 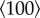 oriented domains, while the scattering peak that shifts from near 550 nm at NA = 0.13 (7.5°) to 525 nm at NA = 0.55–0.7 (33.4°–44.4°) is attributed in turn to the photonic response from the 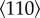 oriented domains. The blue-shift in the latter (iridescence) is owed to the scattered light from off-axis 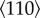 oriented domains, indicating the intensity drops off relatively slowly out to 10° or even 15° angular displacement. We note, albeit to a much lower extent, a concomitant red-shift in wavelength of the 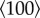 response with increasing NA.

The relative intensities of the blue and green/yellow peaks as a function of NA were estimated by measuring the areas under the corresponding peaks. Inspection shows that as the NA is lowered, the relative scattering in the blue increases. At an NA = 0.13, the ratio of the area under the green/yellow peak to the blue is 1.12. Given that 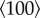 oriented domains in the three mapped wing scales are 4× the area of 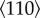 domains (figure 4*c*), then the relative scattering power of 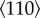 domains is around 4.5× that of the 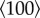 domains. Additionally, at low NA, a shoulder was also observed at 465–470 nm (figure 7*a* NA 0.22; electronic supplementary material, figure S19). This weak band is attributed to the 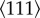 for modelling purposes. However, it could equally be assigned to 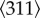 oriented domains on the basis of the similar scattering character observed in masked optical images of the mapped wing scales for both of these respective directions.

It was of high interest to understand the spectral response at the level of individual domains. However, the spectrometer used in this study had insufficient resolution for probing individual domains, particularly at low NA where the integration area on the scale averaged many domains. So, to complement the spectrometric data, pseudo-spectral data were generated from RGB images using wavelength-calibrated optical microscopy with resolution of individual domains over the entire range of available NAs. Figure 7*c* shows a hue histogram extracted from an image of a structurally mapped wing-scale. Figure 7*d* shows the wavelength-calibrated pseudo-spectra derived therefrom (see the calibration curves in electronic supplementary material, figure S20*a*,*b*).

The hue-based wavelengths determined by imaging (as a function of the orientation) were in good agreement with those obtained from the actual spectral data (e.g. compare data in electronic supplementary material, tables S5 and S6). The image-based pseudo-spectral determinations showed that average wavelength of scattering from domains of any orientation at high NA was approximately 520 nm. This close match, despite the absence of intensity information in the image-based pseudo-spectral data can be accounted for by considering the relatively stronger scattering in the 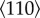 direction as discussed above and in §3.7. The close match also allowed increased confidence in mapping domain orientations using colour images alone, allowing assessment of the probability of finding 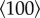 and 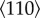 over much larger areas of butterfly wings than could be practically possible by SEM alone.

### 3.5. Modelling the angle-dependent iridescence and photonic response of single gyroids crystals in *Callophrys rubi*

Critical details of the photonic response of the crystals in *C. rubi* can be modelled in solvers to return the band gap (stop gap) map as a function of various parameters such as dielectric constant and volume fraction. A routinely used tool for this is the MIT photonic bands (MPB) software [32] which can return mid-gap frequencies of photonic crystals at normal incidence. Below we show that these same frequencies are conveniently and accurately predicted as a one-dimensional iridescence phenomena related to the dominant {110} planes of the single gyroid.

Maxwell's equations were solved using the MPB package to determine the band structures for the single gyroid. The unit cell was set to 344 nm (the experimentally determined value for scale 3; see electronic supplementary material, table S2) and the dielectric constant of 2.4. Band diagrams for 25 and 30% fill fraction are shown in electronic supplementary material, figure S23*a*,*b*. Additionally, a band gap map constructed from multiple MPB runs with varying volume fraction of chitin (20–50%) is also shown in electronic supplementary material, figure S23*c*. From this latter figure, it is found that a volume fraction of 23% is consistent with the spectral and hue data measured at low NA.

**Figure 8.**
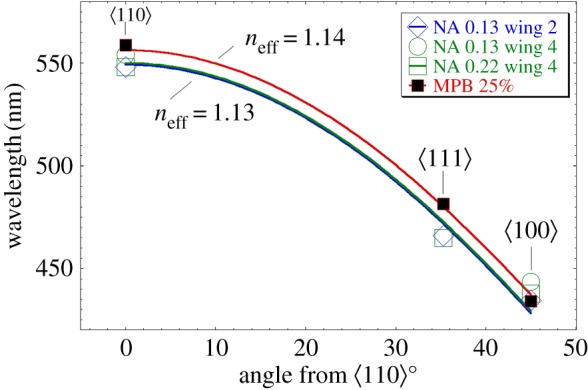
Nonlinear regression fits of the Bragg–Snell law (equation (3.1)) to the observed and simulated MPB data. Open symbols correspond to the wavelengths of the low-NA spectral peaks (figure 7*a*). Filled symbols are mid-gap theoretical wavelengths using MPB (for a unit cell of 344 nm, 25% volume fraction and chitin refractive index of 1.55 (*ɛ* = 2.4)). The best fit to the experimentally determined spectral data returns *n*_eff_ = 1.13, equivalent to a volume fraction of 23%. The best fit of the one-dimensional model to the MPB data returns *n*_eff_ = 1.14 equivalent to a volume fraction of 25%, which was the input value used to generate the MPB data in the first place.

On the other hand, following the alternative one-dimensional approach, spectral data were fit to the Bragg–Snell law for a one-dimensional multilayer reflector (figure 8):3.1
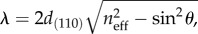
where *d* is the {110} interplanar distance, *n*_eff_ the effective refractive index and *θ* the angle between 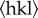 and 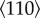.

Wavelengths were determined spectrometrically at low NA and assigned to photonic responses along the 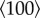, 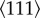 and 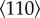 axes from several wing scales, with *d* equal to the measured {110} interplanar distance (i.e. √2 × 344 = 243.2 nm).

Figure 8 shows a single-parameter fit of equation (3.1) to the spectral data, returning an effective refractive index of 1.13. This allowed an estimate of the volume fraction of 23% based on a parallel effective medium approximation for the single gyroid structure (electronic supplementary material, figure S24). The two estimates of volume fraction, i.e. from MPB and from spectra, were in good agreement, showing that a one-dimensional model could predict the wavelengths of observed scattering/iridescence. As Poladian *et al*. [19] noted in estimating the scattering within the first Brillouin zone (BZ), the ratio of highest and lowest reflected frequencies will be approximated by the ratio of the highest and lowest distances in reciprocal space of the rhombic dodecahedra defining the BZ. Therefore, the largest ratio will be the distance √2 ≈ 1.41. Following this approach and given the observed maxima for 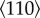 oriented domains occurs near 550 nm, the predicted light scattered from the 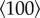 oriented domains is expected to be centred in the UV at 389 nm. This is close to the value of 397 nm found by Michielsen *et al.* [14] using simulations (volume fraction = 0.17, a unit cell of 363 nm and a chitin refractive index *n* = 1.55 + i0.06) in the time domain, but is in contrast with the observed value and that found here using simulation in the frequency domain (MPB), which are both near 440 nm. The ratios found here by observation and simulation in the frequency domain are therefore ≈1.28 instead of √2. This ratio of ≈1.28 can be extracted from the data of others using MPB, e.g. fig. 4.8 and 4.9 of Saba [30] and varies little with fill fraction. Thus, it appears that a one-dimensional multilayer reflector model based on the {110} planes can more accurately predict observed iridescence than one derived from the ratios of distances in reciprocal space or from simulations in the time domain. The one-dimensional model can then be used to construct a reasonable model of the total scattering wavelengths from a single gyroid crystal in *C. rubi*, as will be discussed in the next section.

### 3.6. Stereographic projection of the observed scattering from *Callophrys rubi*

**Figure 9.**
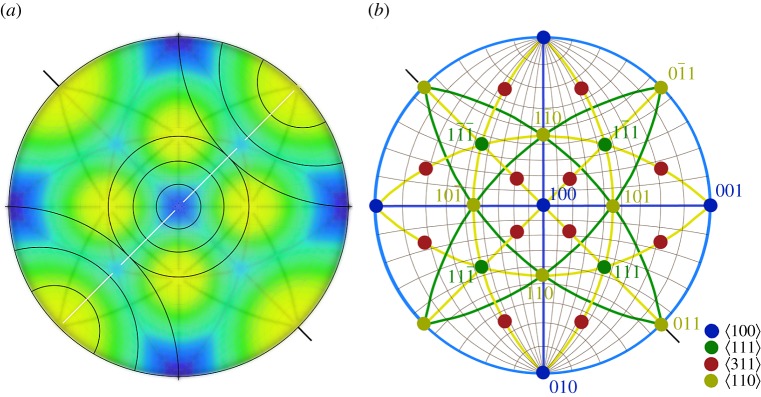
(*a*) Stereographic projection of colour response in *C. rubi*—centred on 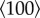. The plot is based on the observed spectral maxima and a one-dimensional model of angle-dependent iridescence from {110} planes, and (*b*) corresponding stereographic plot of crystallographic planes and poles for [100] oriented gyroid crystals. Small circles in (*a*) represent light cones at NAs corresponding to 15°, 30° and 45° centred on the [100] and [011] orientations. The curves in (*b*) are the corresponding traces of the poles (the traces corresponding to the 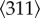 poles are omitted for clarity). The short black lines on the exterior of each diagram represent a 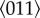 axis, which is a rotation axis by which each of the observed orientations in *C. rubi* can be transformed onto each other because of a common set of {110} planes. This transformation relationship is indicated by the white trace in (*a*) and the yellow traces in (*b*).

The spectral data allowed construction of an experimental stereographic projection of the scattering wavelengths (figure 9). The colours are based on the observed spectral wavelengths and interpolation between these wavelengths using the one-dimensional scattering model of the previous section. Only one hemisphere is shown, which contains 24 repeats of the first BZ, each defined by the edges between the smallest triangles formed between the poles: 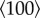, 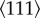 and 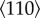. While the 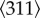 crystallographic projection is not formally in the first BZ, its director can still be co-projected to a pole within the first BZ to illustrate its relative orientation. This is important due to the common observation of 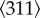 oriented domains.

Figure 9*b* illustrates the angular relationship between the various crystallographic orientations visible in figure 9*a*, including the poles and traces to the Miller planes. For convenience, it is centred on the [100] axis. Note that poles on the outer circle in figure 9*b* represent {*hkl*} planes tilted vertically and the central pole point corresponds to the horizontal (100) planes. Tilt angles on the horizontal axis can be read from the graduated spherical grid. For example the central [100] pole in blue is 45° from the light green [101] and [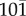] poles as expected. Note that when a 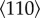 axis is horizontal, as the [011] axis is in figure 9*b*, two sets of {110} planes, i.e. the (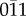), and (011) are vertical and at right angles to each other. This can be seen as the square lattice in the real space image shown in figure 2*b*. Likewise, we can readily show that in each of the observed orientations, at least one set of {110} planes is always vertical, independent of the orientation. This illustrates two facts: (i) that {110} planes are always vertical or near vertical in every domain and (ii) that all observed domain orientations are thus related by rotation around the poles to those {110} planes, i.e. a 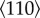 axis.

### 3.7. Pseudo-spectral determination of iridescence in individual photonic crystals

Optical micrographs of scale 3 were masked to collect colour information from domains oriented in only one direction (e.g. figure 7*b*). Wavelength calibration of the sensor then allowed pseudo-spectral wavelength distributions (hue histograms) to be generated as a function of both domain orientation and NA (electronic supplementary material, figures S21 and S22). These pseudo-spectra show that domains of a given orientation scatter with a maximum centred at a particular wavelength. These also show that pseudo-spectra collected at low NA provide a good approximation to the scattering wavelength at zero NA (i.e. perfectly normal incidence and reflection), in close correlation with the spectral and simulated data (figures 8; electronic supplementary material, figure S20*c*).

Evidence for iridescence in the pseudo-spectral data can be seen in electronic supplementary material, figure S20*c* as a systematic shift in the wavelength maxima with increasing NA. This shift towards 520 nm is independent of orientation type. Electronic supplementary material, figure S20*c* shows that the wavelength maxima for the green spectral peak (attributed to 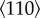 oriented domains) as a function of NA is very similar to those obtained from pseudo-spectra of 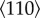 oriented domains. By contrast, the wavelength of the detected blue peak (attributed to 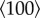 oriented domains) in the spectral data has little NA dependence compared with the pseudo-spectral peaks derived from images of 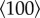 oriented domains. This strongly indicates that the scattering intensity drops off rapidly with angle away from the 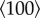 but not so quickly for the more strongly scattering 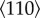 oriented domains. As opposed to the spectral data that contain intensity information, both on- and off-axis scattering contribute equally in the pseudo-spectral data since it is based solely on counting pixels of the same RGB hue range, and disregards intensities. For example, both a bright blue pixel and a dark blue pixel are counted with the same weight in a pseudo-spectrum. A summary of the pseudo-spectral data appears in electronic supplementary material, tables S5 and S6 derived from figures S21 and S22.

### 3.8. Optical determination of gyroid orientations over whole wing scales of *Callophrys rubi*

**Figure 10.**
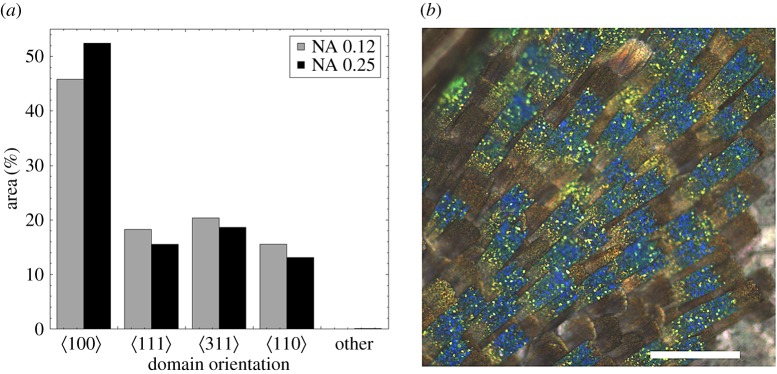
(*a*) Optically determined domain orientation preferences for ∼190 scales (NA 0.12, 5×) and ∼50 scales (NA 0.25, 10×). Note the similarity of the preferential alignment with that determined structurally using SEM (cf. figure 4*c*) (*b*) optical image corresponding to the NA 0.25 data in (*a*). Note that the red/orange and brown areas in (*b*) are where cover scales have been lost. These areas were masked out prior to analysis. Scale bar, 200 µm.

Regions of wings containing approximately 20–250 scales were imaged at low NA from several individual butterflies. Corresponding hue histograms then enabled an accurate estimate of relative coverage of 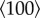 and 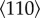 oriented domains (figure 10*a*) over larger areas of wings (figure 10*b*) than would be practically feasible using SEM mapping. This optical method (electronic supplementary material, figures S25–S27) also allowed an estimate of the coverage by 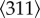 and 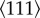 oriented domains, though with less accuracy than the other two directions. Figure 10*a* shows that 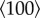 oriented domains cover approximately 40–50% of the observed wing scales, which generalizes the earlier finding that non-random processes are active in determining crystal orientation in *C. rubi*. The same analysis also confirmed that orientation of domains along 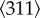, 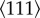 and 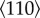 directions was consistent with that expected for a random process (see §3.2). The implications of these coverage rates for formation of single gyroids are discussed in §4.3.

### 3.9. Suppression of iridescence at low magnification

Below some threshold magnification, adjacent green/yellow 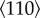 and blue 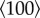 oriented domains will not be resolved. A trichromatic RGB sensor (such as a CCD or a human eye) will then necessarily show the two domains as being of intermediate colour, and the effect of iridescence suppressed. This mechanism is discussed below.

Low magnification (approx. 1×) and low NA (NA angle = approx. 1°) images were collected from whole *C. rubi* wings at a distance of about 6 m with a telephoto lens and a narrow beam of illumination. These images (see electronic supplementary material, figure S28) showed a dominant green colour, slightly blue-shifted when the wing was normal to the view. This green colour is attributed to mixing of blue light from the dominant, but relatively weakly scattering 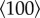 oriented domains and green–yellow light from the less common but significantly more brightly scattering 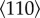 oriented domains. This type of mixing is due to the optical uncertainty in the position of each scattering domain with respect to the optical device and the hue shift associated with averaging two colours in an RGB detector. This phenomenon is thereby the likely mechanism involved in the naked eye observations of green scattering from *C. rubi* and related species when illuminated with a low NA light source. However, the presence of the blue scattering, even below resolution limits, should still be detectable with appropriately tuned vision systems. Thus, the preferred alignment of 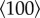 domains maybe useful for conspecific recognition while not compromising the overall green camouflage seen by predators lacking short wavelength discrimination. Indeed, lycaenids have evolved enhanced colour vision in the short wavelength part of the spectrum, with blue cues suggested as an important signal in communication [33].

### 3.10. Extension of the results to other species of butterflies

Image-based colour analysis performed on a selected set of *Lycaenid* butterflies confirmed a similar blue to yellow green iridescence at low to high NA. Several of these species showed a preferred coverage by 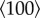 oriented domains. This shows that the findings are not species specific but are found among various species of the genus *Callophrys* and species of other genus such as *Thecla* and possibly *Cyanophrys*. It was not possible to determine if this was applicable to *P. sesostris* and *T. imperialis*, due to their particular added optical filters [23,24] (see electronic supplementary material, figures S29–S33).

## 4. Discussion

### 4.1. The colour and iridescence of single gyroid photonic crystals in *Callophrys rubi*

The non-iridescent colour of *C. rubi* seen from a distance is well accepted to arise from the integrated photonic reflections of thousands of iridescent small crystals oriented in many different directions. Imaging under low NA conditions at normal incidence shows that the domain type covering most of the visible surface are those reflecting blue light, with occasional domains reflecting bright yellow–green light. SEM mosaic images of scales show clear crystal lattices which domain boundaries could be readily mapped. By correlating the domain boundaries mapped onto these SEM images with optical images of the very same scales, we confidently concluded that regions of blue and green–yellow scattering always fell within the same boundaries as those constructed in the structural domains maps. Furthermore, the direct correlation of certain identifiable symmetries present in SEM images with the low NA optical responses allowed us to experimentally confirm that real (not theoretical) single gyroid photonic crystals in butterflies reflect wavelengths along certain crystal directions in good accordance with Maxwell's laws. Therefore, this work has established the first experimental evidence, directly correlating iridescent scattering behaviour with crystal orientation for three-dimensional gyroidal photonic crystals at visible wavelengths.

The scattering from 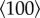 and 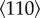 domains at low NA have maxima at 440 nm and 550 nm, respectively. To explain the net overall green colour observed at a distance, it had been previously proposed [34] that multiple scattering arising from domain boundaries erases the ‘memory’ of the angle of incidence, thus homogenizing the overall scattering response. However, the grains boundaries in *C. rubi* provide limited dielectric contrast for strong multiple scattering to occur, so any change in the angle of the scattering at the boundaries will remain largely undetectable. This is deduced from the fact that the crystals are intimately associated, and in essence topologically continuous as observed in cross-section polished SEM images (see, for instance, figure 1*e*). Notice that the bright yellow green scattered colours in, for example, figure 6*c* have sharp borders and are contained within the structurally determined domain boundaries. This is inconsistent with strong scattering from domain boundaries.

Instead, we suggest that the dominant green colour observed in the far field results from a mixing of colour from the individual domains when these are no longer optically resolved. This is analogous to the pointillist way colour is generated on computer or TV screens via the mixing of red, green and blue pixels near or beyond their resolution limit. With higher concentrations of larger optically resolved domains on the wing surfaces, colour demixing would be expected, with a concomitant increase in local iridescence.

Studies relating to taxa in which colour is produced by polycrystalline single diamond, rather than single gyroid domains, with sizes below the optical resolution limit show a similar effect to what is seen in *C. rubi* [7,35,36].

### 4.2. Preferred orientation of gyroid crystals in *Lycaenid* and *Papilinoid* butterflies

Three-dimensional photonic crystals in the wing scales of the butterfly, *C. rubi*, are dominantly oriented with their respective 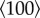 axes normal to the surface of the scales, but not exclusively, as has been explicitly noted previously by others, albeit using smaller datasets [28,37]. This non-exclusive dominance by 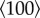 oriented domains is also very likely occurring in other dull green *Lycaenid* butterflies, as was inferred here after a preliminary examination of other individuals. This particular mode of preferred orientation leads to the suppression of iridescence, showing instead a uniform coloration, even with some variation in the relative concentration of crystal orientations. However, this variation must be necessarily limited. For example, without incorporation of other optical elements, a relatively high concentration of the brightly scattering 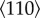 oriented domains will lead to non-uniform coloration (including iridescent areas if clusters large enough to be resolved are formed), and also to less intense scattering from the wing scale when observed off-axis because the brightest scattering will become restricted to the direction normal to the wing scales.

Nonetheless, this is not the only known strategy employed by butterflies for producing dull green colours using polycrystalline single gyroids with similar single-crystal optical properties to *C. rubi*. For example, preferred alignment occurs exclusively along 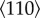 and 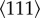 axes in the dull green wing scales of the *Papilinoid* butterflies, *P. sesostris* and *T. imperialis*, respectively [23,24]. The former should reflect all but green in every direction away from the wing surface normal, and the latter should appear quite dark at normal incidence, given the poor reflectivity found here along the 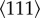. However, the non-iridescent green colour of these particular *Papilinoids* is owed to poorly understood optical structures overlaying the gyroidal lumens that act as diffusers or waveguides [23,24]. Clearly, different strategies have evolved to achieve the same dull green colour using otherwise similarly iridescent photonic crystals in distantly related butterflies.

Nevertheless, these distant relatives show similarities in their in-plane or azimuthal crystal orientational characteristics: first, the individual crystals are all azimuthally isotropic, so in this respect the degree of in-plane control the organisms exerts over the gyroids appears neutral. Second, there is a large propensity in the three species to align their respective tunnel axes approximately parallel to the surface normal. This alignment of the pore system seems to be the rule, without an obvious explanation. Third, as a consequence of the particular preference the three species show for aligning their 〈100〉, 〈110〉, 〈111〉 or 〈311〉 crystal axes approximately normal to the surface, one or more sets of {110} Bragg planes are necessarily aligned approximately parallel to the surface normal. In the case where domains are aligned with a 〈111〉 axis normal to the surface, three sets of {110} Bragg planes are parallel to the surface normal. For 〈100〉 alignment, two sets of {110} Bragg planes are parallel to the surface normal. For 〈110〉 and 〈311〉 alignment, a single set of {110} Bragg planes are parallel to the surface normal.

Therefore, a general construction rule for the orientation of domains in the wing scale of *C. rubi, P. sesostris* and *T. imperialis*, appears to be keeping the {110} Bragg planes normal to the surface. In this way, *C. rubi* is a 90° rotated variant on that of *P. sesostris.* This rotation of domains is illustrated in electronic supplementary material, figure S34 and movie S1, where the gyroid structure is rotated around a single 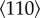 axis and the four orientations, seen in figure 2*b*–*l*, are rotated into view.

### 4.3. Formation of gyroids in butterflies

Single gyroid structure formation in viable wing-scale cells of *C. rubi* and other organisms is thought to proceed via a poorly understood in-folding of the plasma and smooth endoplasmic reticulum membranes followed by, or concurrently with, their chitinization [21,38,39].

Our observations and measurements place some limits on the formation and location of gyroids within the scales of *C. rubi*. For example, the ordered regions of polycrystalline gyroids are restricted to an area of 43% at the distal end of the scale (e.g. electronic supplementary material, table S1 and figures S1*b* and S2*f*). Further, the weak but positive correlation found between the rib/cross-rib spacings and the ordered length (electronic supplementary material, figure S2) suggests the rib spacing widens in concert with the development of the underlying ordered material and does not reach its final spacing until the phenomena that induces order had become well developed, despite the fact the ribs and cross-ribs appear to be well formed prior to the onset of gyroid formation [39].

We have also found that the domain size/area distribution is distinctly bimodal (figure 4*a*,*b* and electronic supplementary material, figure S13), with larger domains at the ordered distal end of the scale and small domains towards the disordered proximal end. However, the area-weighted distribution, being less sensitive to the population of small domains, is well fit by a single lognormal curve, peaking at approximately 20 µm^2^ per domain (electronic supplementary material, figure S5). The lognormal distribution is consistent with a process of random nucleation and growth of the gyroid crystals [40]. Domain size within the ordered regions of the three mapped scales was found to have a slight dependence on the perpendicular distance from the marginal striae, including the teeth (not shown) indicating slightly early nucleation, smaller diffusion lengths/faster growth rates closer to the walls and/or underlying cytoskeletal actin filaments [41].

Domain junctions in the ordered regions were generally similar to plateau junctions in two-dimensional foams. The tunnel and walls of neighbouring domains in the ordered regions were typically fused and topologically continuous across domain boundaries indicative that single gyroid crystallization had not terminated before the growing domains collided allowing some lowering of the energy of the boundaries. In most cases, three neighbouring domains form triple junctions, as in two-dimensional foams, with four-way junctions being far less common, but when present, often containing crystallographically disordered material or defining a hole in the polycrystalline structure.

By contrast, domains in disordered regions peak in the number-weighted distribution between 5 and 10 µm^2^ per domain (electronic supplementary material, figure S13). Domains become more separated towards the proximal end, and are characterized by a random bicontinuous structure similar to the perforated and random sponge networks seen in other butterfly species [28,42]. In the thinnest, disordered parts of the scales, a distinct layered anisotropy in the random cuticle structure can often be seen in cross-section SEM images, synonymous with a pseudo-lamellar structure intermediate between a random bicontinuous sponge and a lamellar phase in the language of self-assembled liquid crystalline phases [43].

The measurements of the scale thicknesses in the ordered region towards the distal end averaged 2.43 ± 0.76 µm, whereas in the disordered region towards the proximal end averaged 0.83 ± 0.28 µm. This compares with thicknesses for *P. sesostris* of 4.3 ± 0.3 µm (data extracted from their published figure [23]) and for *T. imperialis* of 3.0 ± 0.7 µm (determined by XRD [24]). Scales from these two species have larger ordered regions (estimated to be up to 90% compared with 43% found here for *C. rubi*). Domains appear to be uniform in size throughout their respective scales, suggesting that these butterflies have control mechanisms for growth and orientation of gyroid crystals different to that of *C. rubi*.

There is also a notable and characteristic relative enrichment of 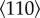 oriented domains in the disordered regions in the wing scales of *C. rubi* and in other butterflies examined here, namely *C. rubi fervida*, *C. rubi sibirica*, *C. apama*, *C. dumetorum*, *Cy. amyntor* and *Cy. acaste*.

The strong correlation of increased frequency of 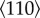 oriented domains in the thinner, otherwise disordered region in the scales of *C. rubi* suggest that this orientation is effectively the only stable one in the thinner regions. This suggest the upper and/or lower bounding plates may exert an influence in the thinner regions, also a notion supported by the observation here of pseudo-lamellar structures, reminiscent of the thin film reflectors of *Celastrina ladon* [44], running parallel to the lower lamina.

According to Ghiradella [44], the viable wing scale cell retracts back through the socket cell into the epithelial cell in the wing membrane during the later stages of the scale cell development. Given that the distribution, orientation, degree of order and thickness of the domains seem to have an axial dependency along the length of the scale, this transition from ordered to disordered suggests that ordering is lost as the retraction of the cell nears the proximal end. A possible explanation could be that the cell enters into a pre-senescence/pre-apoptotic phase, the exact point along the scale apparently coinciding with the overlap of the other tiled scales, so as to avoid wasting valuable energy producing further ordered domains. The very existence of the transition zone around half way along the scale is highly suggestive that the cost is high either biochemically or in added weight. Indeed, the relationship between the cost of manufacture and the overlap zone points to the fact that these two factors are likely traded off. Formation of poorly ordered gyroids or none at all in the overlap area of scales may also be a consequence of the higher humidity (lower drying rates) and limited access to oxygen expected in that region.

Finally, the finding of a single axis relating all the seen orientations in *C. rubi* (figure 3*d*–*g*) may have implications for the formation of single gyroids in their wing scales. Taken as a whole, this image indicates that not only do all of the domains in scale 1 fall into distinct preferred alignments but that these are related by rotation around a single high symmetry axis. Given the unusual finding of significant alignment of domains along the high order 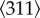 direction, this indicates that the high symmetry axis in question that relates all of these domains is in fact a 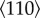 axis, and such a restriction is worthy of further investigation as to assess if this is due to a biological or purely physical mechanism.

### 4.4. Implications for chiral analysis

Noting that single gyroid crystals are chiral in the dominant 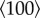 direction, and that this is predicted to produce a strong, structurally dependent preferential reflection of circularly polarized light [30,45], a natural question arises as to whether chiral signalling could have evolved in butterflies. In a future paper, we will examine why this chiral band gap has not been seen [46,47] and explore the possibilities for detecting chiral optical photonic responses from butterflies.

## 5. Conclusion

High-resolution structural maps of single gyroid domains in the cover wing scales of *C. rubi* show that 200–300 domains occur in each scale, each having an area of 15–20 µm^2^. Single gyroids were preferentially oriented along four distinct crystal axes, with 40–50% of these along 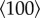 axes suggesting some biological process is involved. The remainder were oriented along 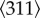, 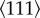 and 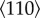 axes. These axes are related by rotation around a single 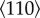 axis, hinting at a further biological control. Domains are otherwise randomly oriented in the plane of the scales with an average unit cell parameter of 341 ± 3 nm.

The intrinsic, angular-dependent colour response (iridescence) of single gyroids was experimentally determined by correlating the low NA scattering colour of hundreds of domains with their previously determined orientations. The iridescent wavelengths found closely matched theoretical predictions, confirming the general high quality of crystals contributing to the colour of *C. rubi* and providing the first experimental evidence for the correlation of photonic response as a function of crystal orientation for any single gyroid, either engineered or natural.

Low NA blue scattering (440 nm) correlated with the dominant 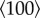 oriented crystals. Bright green–yellow scattering (550 nm) correlated with the less abundant 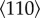 oriented domains. Scattering from 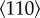 oriented crystals was approximately 4.5 times brighter than from 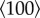 oriented crystals. The 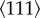 and 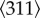 oriented domains scattered weakly in the cyan.

The coverage of single gyroids oriented along 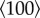 and 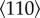 found by the structural mapping was then confirmed over larger areas incorporating approximately 10^4^–10^5^ domains per image, using the correlation between colour and structure. Preliminary analysis of species of other *Lycaenid* butterflies using this method confirmed both the propensity of other gyroid-containing species to use a similar mode of preferred alignment for their colour, and the generality of the method.

Imaging the wings of *C. rubi* at low NA and below the Rayleigh limit for resolving individual domains also resulted in predominantly green scattering, suggesting that very few brighter green–yellow 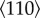 oriented domains are required to generate the green colour perpendicular to the line of sight. However, in most cases, the line of sight will not be perpendicular. This implies that the preferred alignment along the 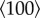 plays an important role in distributing the scattering in a solid angle centred near 45° from the surface normal of the wings, and complemented at smaller and larger scattering angles by domains of the other observed orientation, enabling very efficient suppression of iridescence.

## Supplementary Material

Supporting Information file

## Supplementary Material

High resolution layered image of scale 1

## Supplementary Material

High resolution layered image of scale 2

## Supplementary Material

High resolution layered image of scale 3
