# Supplementary material for: On the colour of wing scales in butterflies: iridescence and preferred orientation of single gyroid photonic crystals
Source: Interface Focus. 2017 Jun 16;7(4):20160154. doi: 10.1098/rsfs.2016.0154 (PMC5474040; doi:10.1098/rsfs.2016.0154)
Supplement: Supporting Information file [file rsfs20160154supp1.pdf]

SUPPORTING INFORMATION

Journal of the Royal Society Interface Focus

http://dx.doi.org/10.1098/rsfs.2016.0154

On the colour of wing scales in butterflies: iridescence and preferred orientation of single-gyroid photonic crystals

Robert W. Corkery[#]* and Eric C. Tyrode[#]

[#]Department of Chemistry, School of Chemical Science and Engineering, KTH Royal Institute of Technology, SE-100 44 Stockholm, Sweden.
*Department of Applied Mathematics, Research School of Physics and Engineering, The Australian National University, Canberra, ACT 2601, Australia

* Corresponding author: corkery@kth.se

† Additional Electronic Supplementary Information available: Animation of the structure rotating around one axis, and full-size, high resolution composite SEM and optical images of wing scales 1, 2 and 3 in layered format (.tif file).

Content: **Pages**

- Section 1: Wing/scale structural analysis and measurements. *2-5*
- Section 2: Mapping of crystal domains: boundaries and areas. *6-8*
- Section 3: Symmetry relations of 2D projections of 3D single gyroids. *9-13*
- Section 4: Domain orientation and probabilities. *14-17*
- Section 5: Correlation between scattering angles and crystal structure. *18-20*
- Section 6: Spectral response. *21-26*
- Section 7: Modeling of photonic response. *27-28*
- Section 8: Determination of orientation distributions from colour images. *29-32*
- Section 9: Telephoto imaging *C. rubi*. *33*
- Section 10: Extension to other butterfly species. *34-35*
- Section 11: Movie description. *36*
- Section 12: Mapping of crystal domains: orientations *37-54*
- Section 13: Notation for describing crystallographic axes and planes 55
- Section 14: References 55

Section 1. Wing/scale structural analysis and measurements

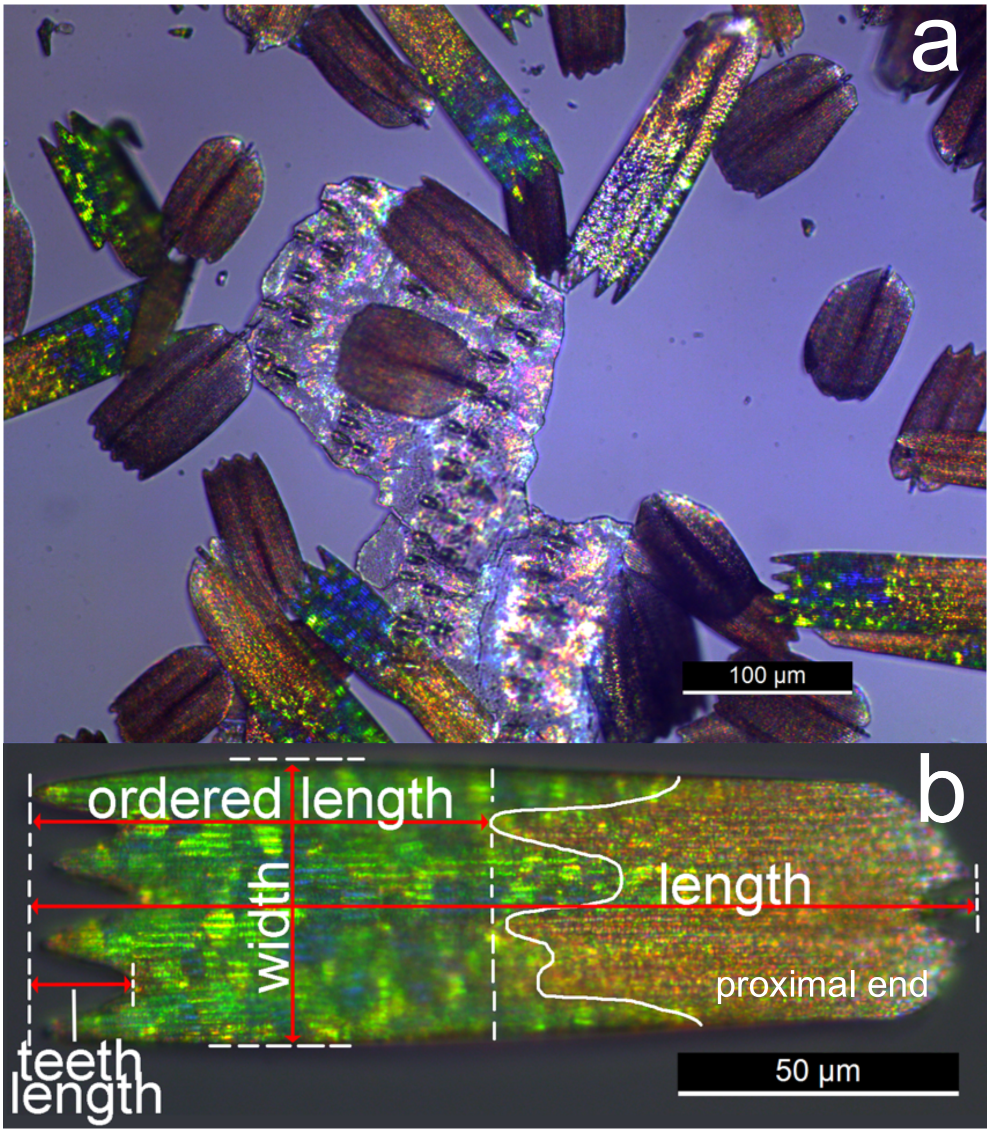

***Figure S1**. **(a)** Portion of a wing showing ground and cover scales and the sockets into which they are anchored. **(b)** Single wing scale (cover scale) from the lycaenid butterfly Callophrys rubi (C. rubi), showing its main features. Note the coloured section to the left (distal end) and the non-coloured section to the right (proximal end). The coloured section contains multiple single gyroid crystals in various orientations.*

Table S1. Summary of measurements on individual wing scales of *C. rubi*

Scale measure	scales measured*	Mean	Std dev.
Length μm	99	171	22
Width μm	117	48	8
Ordered length μm	116	77	16
Teeth length μm	115	15.4	3.8
Teeth per scale	112	3.9	0.4
Length/width ratio	98	3.68	0.52
Ordered length/total length	99	0.43	0.06
Tooth length/total length	98	0.09	0.01
Ordered rib spacing μm ‡	37 (20)	1.93 (1.94)	0.09 (0.11)
Disordered rib spacing μm ‡	25 (6)	1.92 (1.92)	0.09 (0.08)
Cross rib spacing μm ‡	(20)	(0.69)	(0.02)

Scale measure	Sections measured**	Mean	Std dev.
Thickness (distal) μm	76	2.43	0.76
Thickness (proximal) μm	133	0.83	0.28

** numbers in parenthesis are determined at high magnification i.e. using 50-100x objectives.*
‡ rib spacings determined from power spectra using FFT
*** determined by SEM cross sections for only 5-10 scales*

Details of the *C. rubi* optical microscopy based scale-statistics:

General description: a total of 119 unique scales that had been previously randomly sampled and placed on glass slides were photographed and measured. Each measurement of distance was made manually using a digital ruler in Image J based on the scale bar that was imprinted in the original photographs taken with the Leica microscope and camera. Measurements were made with an estimated accuracy of better than 3 pixels. This is the basis for the estimated error.

Scale length was measured along the longitudinal axis from the longest distal tooth to either the proximal lobe or the attachment pedicle. The scale width was measured at the widest part of the scale, orthogonal to the longitudinal axis. The scale colour length was measured like the full scale length. It is defined as the distance from the furthest distal tooth to the transition to disordered photonic reflection (see Figure S1b). The post transition region is marked at an NA of 0.25-0.4 (10-20x magnification. See Table S3) by the notable quick reduction in blue scattering and the rise of both yellow and red/brown scattering from notably smaller domains. At higher NAs (i.e. 0.75-0.85 corresponding to a 50x-100x magnification), the transition was more easily seen as the rise of yellow/red/brown scattering. Often the transition is a curved boundary, the ordered regions running further along the marginal stria than the center. However, it was commonly observed that a third lobe of order ran further along the scale following the centerline. The effect is like three-pronged ordered region inter-grown into a two pronged disordered region. The margin was estimated as the position to which the one or two disordered prongs became substantially evident. Teeth length was taken as the largest distance between adjacent distal and proximal points of one distal tooth measured parallel to the longitudinal axis. The number of teeth was also recorded.

Additionally, power spectra were generated as mentioned in the experimental section of the main article using the FFT algorithm in Image J in order to measure the rib spacing (20-100x) and also the cross rib periodicity (50-100x). The resulting 2D power spectra images allowed frequency space measurements and the positions of maxima to be tabulated. The measurement errors were estimated by repeat measurements and noting the variability. When possible, FFT measurements were taken separately from the ordered and disordered regions.

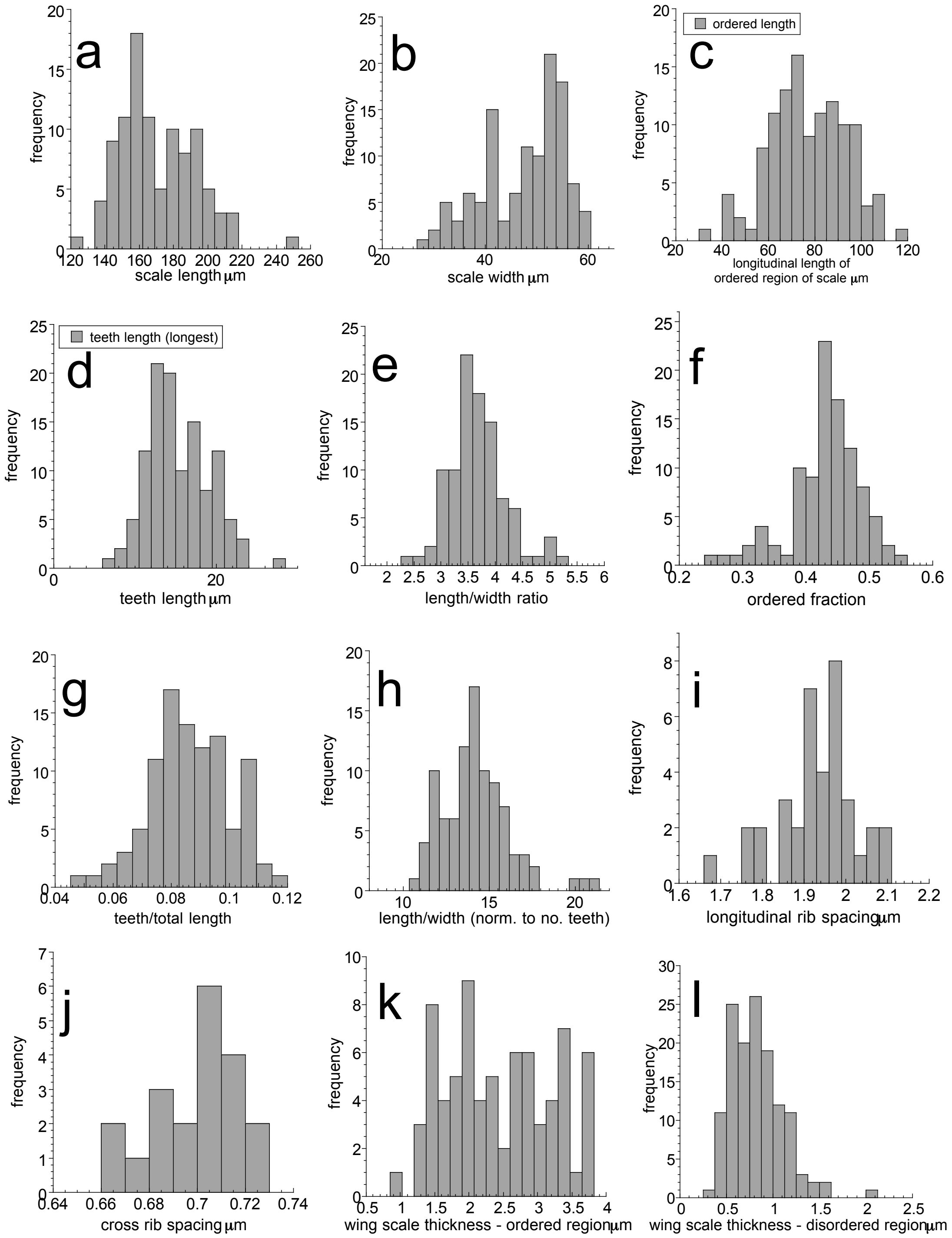

Figure S2. (a-l) *Histograms of various measurements obtained for the wing scales of C. rubi examined here and summarized in Table S1.*

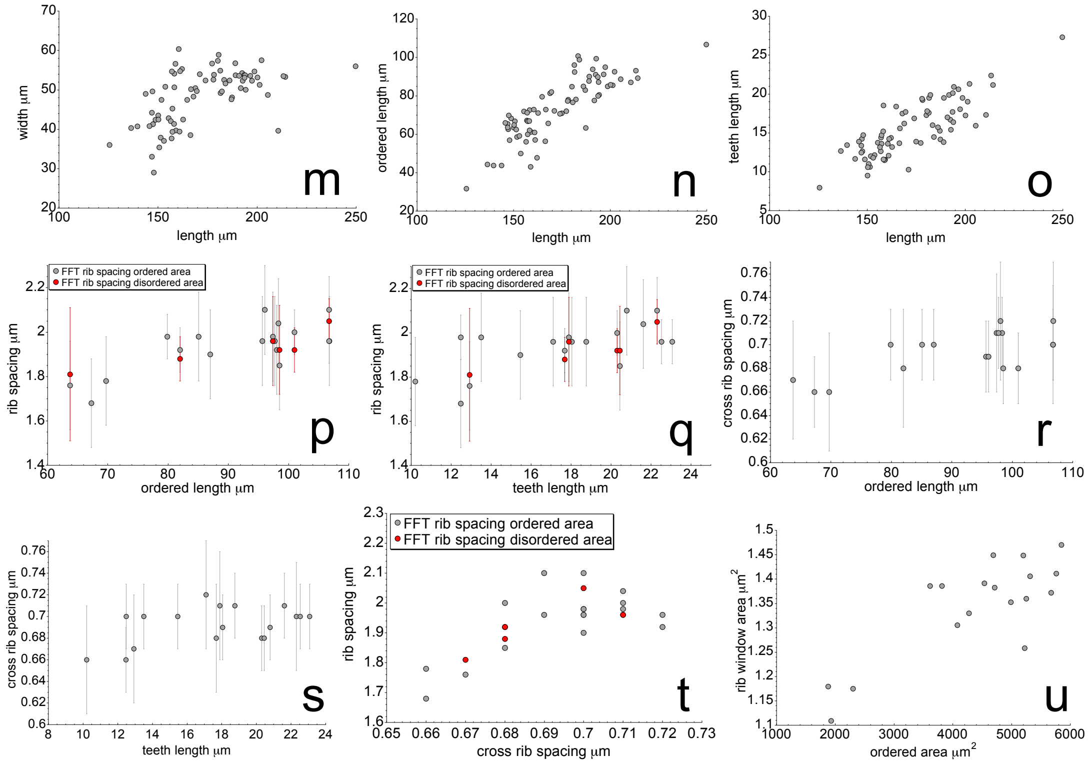

Figure S2(m-u) *Scatter plots of various measurements for wing scales of C. rubi (data for scales with four teeth only).*

There appears to be a weak trend that suggests that as the length of the ordered area of the scales become longer, both the rib spacing (Figure S2p) and cross rib spacing (Figure S2q) becomes wider. This is also reflected in the fact that wider rib spacing correlates to wider cross rib spacing (Figure S2t). This suggests that the rib spacing is modulated by the development of the underlying ordered material and does not reach its final spacing until the phenomena that induces order is well developed.

Further, there is a very strong and clear correlation of teeth length versus total length of the scale (Figure S2o). This is a very clear indication that the controlling factor for teeth length is the same as that for total scale length. In this way the teeth lengths are an indicator of the maturity of the scale. Likewise, a similar statement can be said for the length of the ordered region versus teeth length, consistent with more colour developing as the scale grows.

Section 2. Mapping of crystal domains: boundaries and sizes

Domain boundaries were straightforward to map by careful inspection. A typical representative area is shown in Figure S3a. With practice, the domain boundaries were easily identified, aided primarily by the ability of human vision to detect rows of aligned tunnels or holes (black in Figure S3a) until they terminate or change direction. These terminations can then be used to accurately define the boundaries. Often the boundaries were faceted or stepped, but no systematic study of this phenomena was undertaken (see for instance Figure S3a, S4 and S12). Larger holes in the otherwise gyroidal polycrystalline mosaic were exclusively found on domain boundaries and these larger holes were often seen to form networks at the vertices of multi-domain junctions. As explained in detail in Section 13 below, for the determination of crystal orientation for a given domain, the most easily assigned were those with their respective <100> or <111> axes normal to the surface of the scale. These are exemplified in Figure S3a (see Section 12 for a thorough explanation of the procedure).

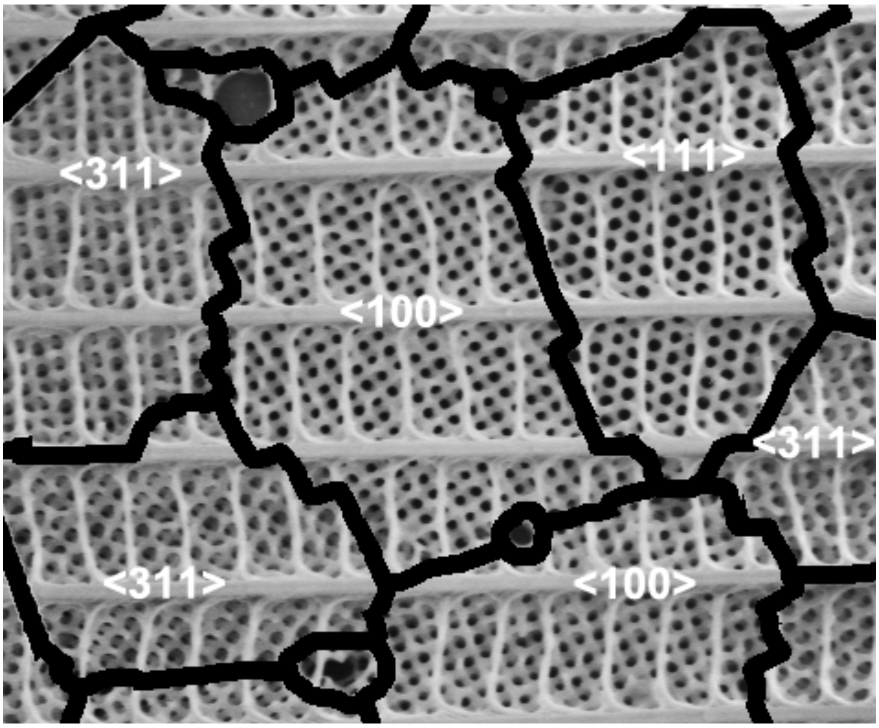

Figure S3a. *SEM image of a colored wing scale from C. rubi with typical domain boundaries shown. Domain crystal orientation are also shown in white (see Section 12 for details)*

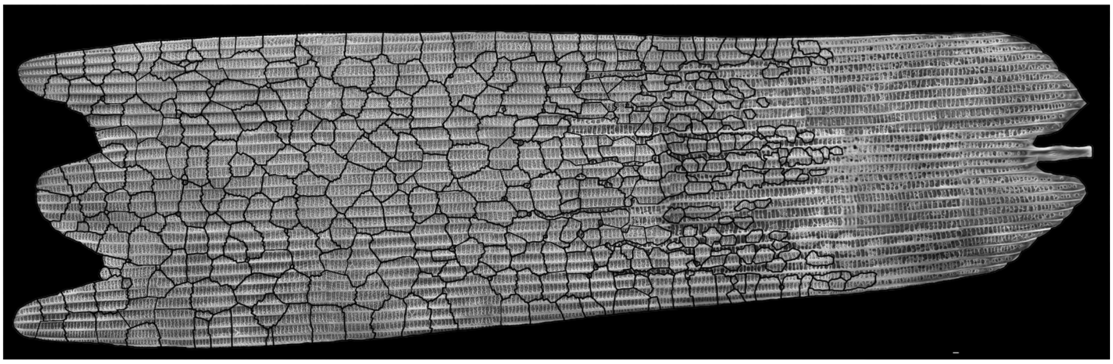

Figure S4. *Composite SEM micrograph of a single ventral wing scale from C. rubi. The SEM image is composed of 21 separate SEM images with resolution of ca. 10nm.*

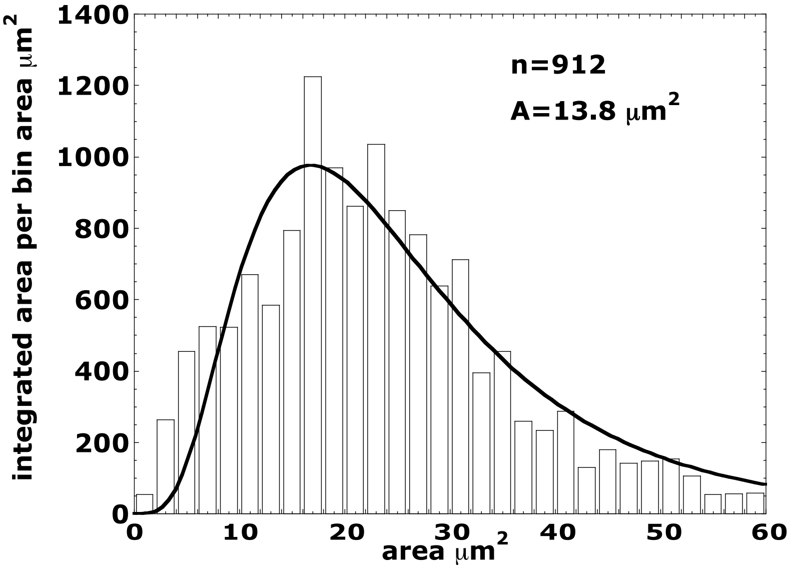

Figure S5. *Area-weighted histogram of area per domain (n=912) found for the three SEM-mapped wing scales of C. rubi.*

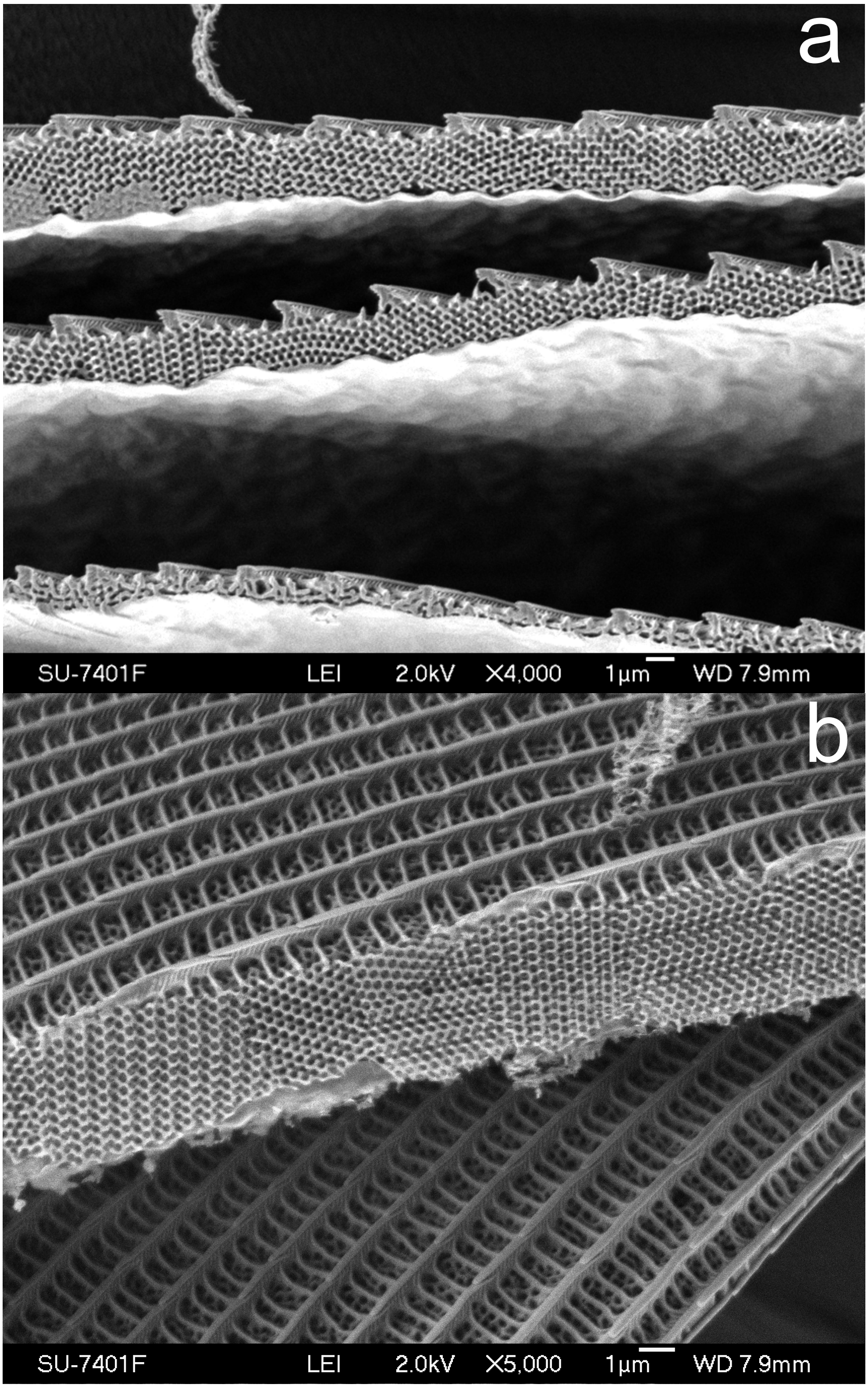

Figure S6(a,b). *Cross-section SEM images through wing scales of C. rubi. In the top image three scales were fortuitously captured in a single cut. The cuts occur at different positions along the wing and illustrate the general finding that ordered gyroids transition to disordered random bicontinuous structures as the scales become thinner. The lower image clearly illustrates the polycrystalline nature of the single gyroid photonic crystals in C. rubi.*

Section 3. Symmetry relations of 2D projections of 3D single gyroids

Four projections of the single gyroid structure are considered below, namely those projections along the <100>, <111>, <110> and <311> crystallographic axes.

a) Projection on the <100> axis

The single crystal FFT pattern in Figure 2d of the main text corresponds to that of a plane lattice projected along <100> (Figure 2c) with side: $d_{10} = \mathbf{a_{sq}} = \mathbf{a_g}/\sqrt{2} = d_{110}$.

The highest frequency point-like maxima of the 2D plane group with Miller index {hk}=<10> are analysed as Bragg spots with inverse distance from the origin corresponding to the interplanar spacing of {110} planes within the 3D single-gyroid crystal structure. Similarly: $d_{11}=\mathbf{a_{sq}}/\sqrt{\mathbf{2}} = \mathbf{a_g}/\sqrt{4} = d_{200}$. Therefore, the general relationship is:

$$d_{sq(hk)} = \mathbf{a_{sq}}/\sqrt{(h^2+k^2)} = \mathbf{a_g}/\sqrt{(2(h^2+k^2))},$$

where h and k are the miller indices of the plane group.

For the plane square group (equivalent to <100> oriented domains in projection), we therefore expect the following sequence for the denominator of the plane square group: $\sqrt{(h^2+k^2)}$ = 1, √2, √4, √5, √8, √9, √10, √13. For the 3D group of the single gyroid, $I4_132$, the sequence for the 2D plane group above corresponds to the following sequence: $\sqrt{(h^2+k^2+l^2)}$ = √2, √4, √8 √10, √16, √18, √20, √26.

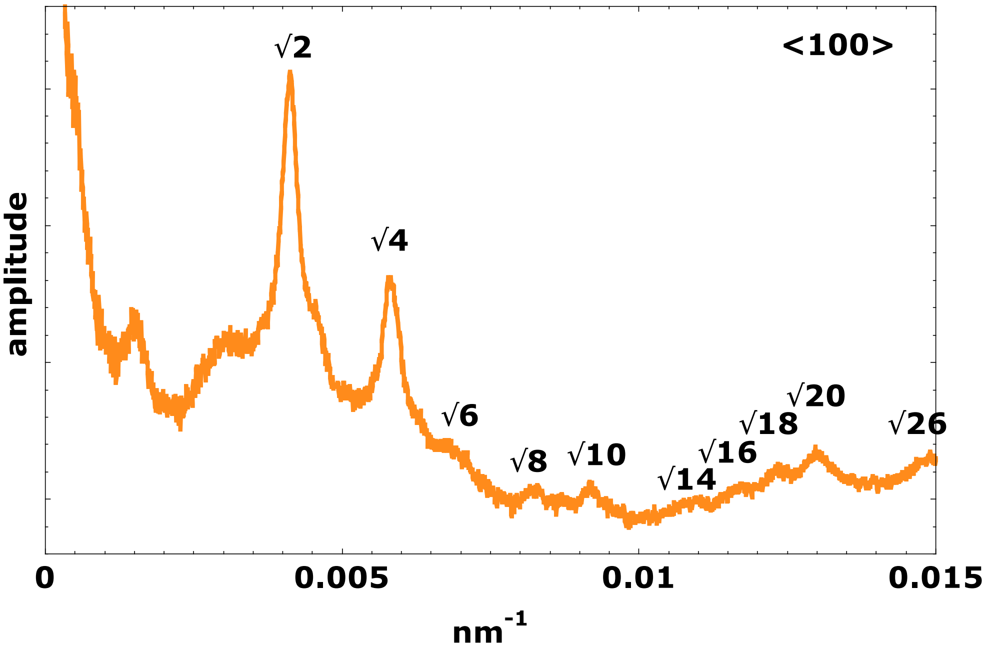

Figure S7. *Radially integrated FFT from wing scale 1. The FFT was obtained after masking out all but <100> oriented domains (see Figures S15 and S16a for corresponding masked image and FFT, respectively). The expected peaks for the projection along <100> are all present. In addition, two very weak peaks labeled '√6' and '√14' appear. The former likely arises from tilt variations of 5-10° in the direction of the <311> axis. The latter is likely due to 3D features of the projection that do not exist in a simple 2D square lattice or to a very small number of mis-assigned domains.*

b) Projection on the <111> axis

For domains oriented along <111>, we can use the same argument, knowing that $d_{hex(10)}=\mathbf{a}_{sq}$, to construct the following relationship:

$$d_{hex(hk)} = \mathbf{a}_{sq}/\sqrt{(h^2+hk+k^2)} = \mathbf{a}_g/\sqrt{(2(h^2+hk+k^2))}.$$

This yields the following sequence of maxima for the 3D group for the FFT of <111> oriented domains: $\sqrt{(h^2+k^2+l^2)} = \sqrt{2}, \sqrt{6}, \sqrt{8}, \sqrt{14}, \sqrt{18}, \sqrt{24}, \sqrt{26}$.

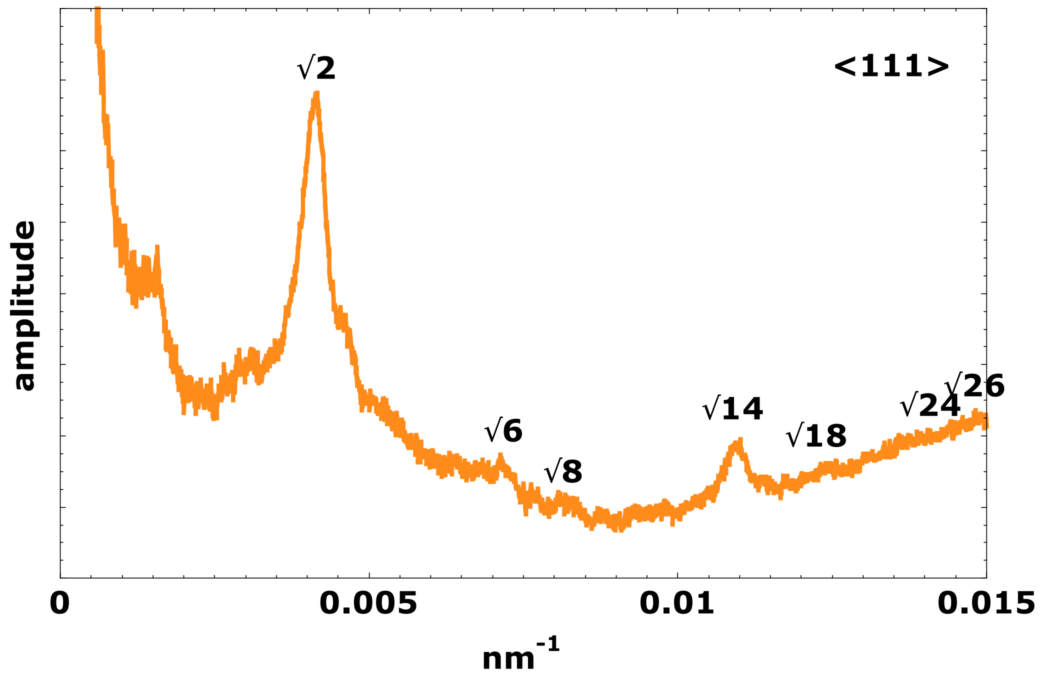

Figure S8. *Radially integrated FFT from wing scale 1 (see corresponding FFT in Figure S16a and example of the masking procedure in Figure S15). The FFT was obtained after masking out all but <111> oriented domains in wing scale 1. The main peaks expected for the projection along <111> are all present. Note that the $\sqrt{24}$, $\sqrt{26}$ peaks are poorly resolved.*

c) Projection on the <110> axis

For the <110> oriented domains the projected lattice of holes has rectangular plane symmetry with sides **a**/√**4** {200} planes and **a**/√2 {110} planes. From this we expect the following sequence: $\sqrt{(h^2+k^2+l^2)}$ = √2, √4, √6, √8, √12, √16, √18, √22, √24.

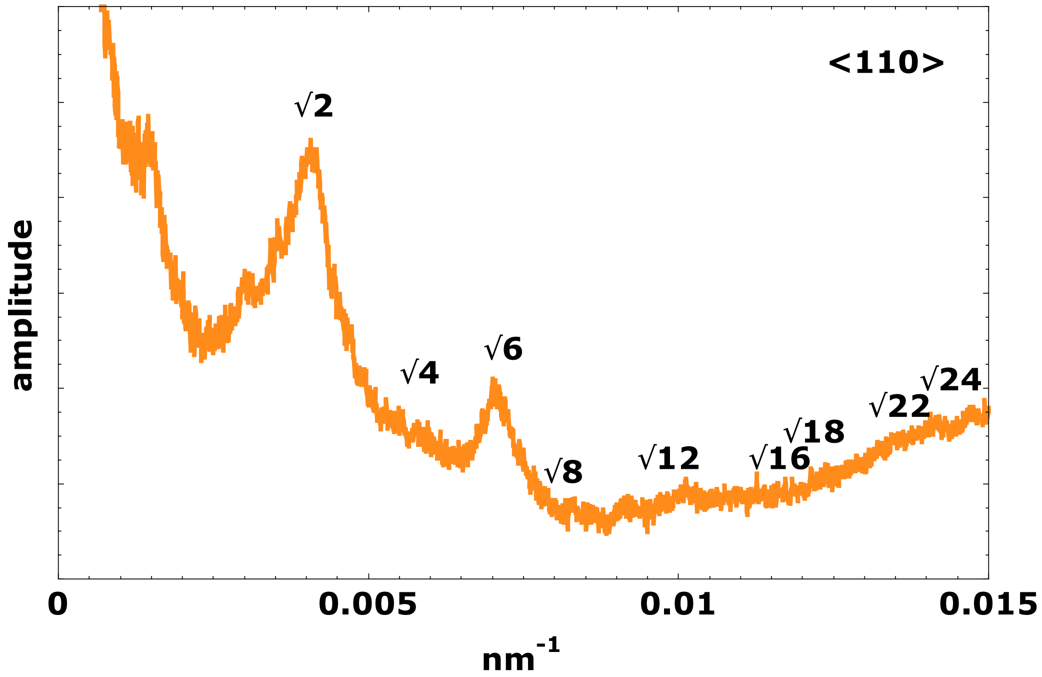

Figure S9. *Radially integrated FFT from wing scale 1. The FFT was obtained after masking out all but <110> oriented domains in wing scale 1 (see corresponding FFT in Figure S16a and example of the masking procedure in Figure S15). The expected √4 and higher order peaks beyond √6 are weak due to low image contrast of features defining the {200} planes at the resolution they were collected, and due to the more distorted lattices typical of domains in this orientation.*

d) Projection on the <311> axis

For the <311> oriented domains we have a plane oblique group with basal spacings $a/\sqrt{2}$ {100} planes and $a/\sqrt{6}$ {211} planes and angle $\cos^{-1}(1/(2\sqrt{3}))=73.22°$: $\sqrt{(h^2+k^2+l^2)} = \sqrt{2}, \sqrt{6}, \sqrt{10}, \sqrt{18}, \sqrt{22}, \sqrt{24}$.

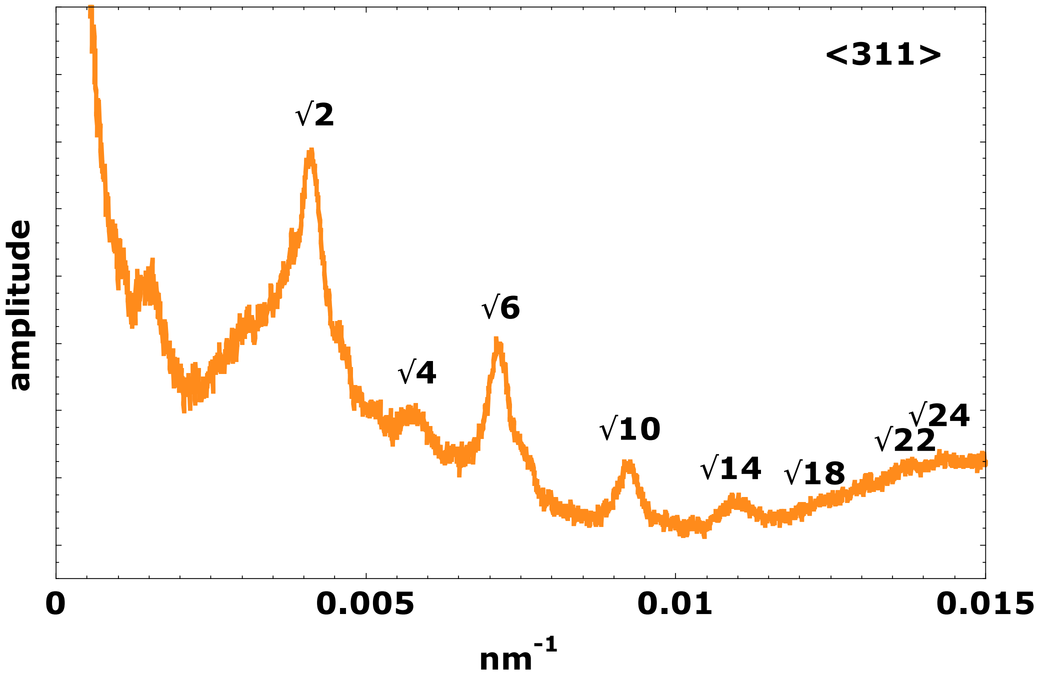

***Figure S10**. Radially integrated FFT from wing scale 1. The FFT was obtained after masking out all but <311> oriented domains in wing scale 1 (see corresponding FFT in Figure S16a and example of the masking procedure in Figure S15). The expected peaks for the projection along <311> are all present. In addition, two small peaks labeled '√4' and '√14' appear. These represent a very small fraction of the integrated area under all the peaks. These minor peaks arise from tilt variations of 5-10° in the direction of the <100> and <111> axes, respectively.*

Each member of the union of all sequences above for each of the orientations, namely $\sqrt{(h^2+k^2+l^2)} = \sqrt{2n}$, where n=1,10 are seen in the sequence of radially integrated FFTs maxima obtained from the respective projected images of wing scales 1 and 2 shown in Figure 5.

Simulated scale

Simulated projections for each of the four observed gyroid orientations were made into the mosaic shown in Figures S11a,b. Each of the projected domains was then placed onto a blank background with a random rotation until the entire area was covered. The frequency of occurrence of each of the four projections in the simulated scale was approximately matched to that observed in scale 1, according to Figure 4c. This was then Fourier transformed to yield a precursor to the simulated frequency domain image shown on the left side of Figure 2a.

Ribs and cross ribs were then added on in parallel sets of straight lines that were subsequently blurred (Gaussian) and geometrically distorted using an iterative trial and error process until a good match was found between the simulated and observed Fourier transforms (see Figures 2a & S16a). Fourier transforms of domains of the same orientation were then generated by masking out all domains of other orientations. The corresponding radial integrations of these Fourier transformed projections then gave good matches to the predicted values shown in section S3.

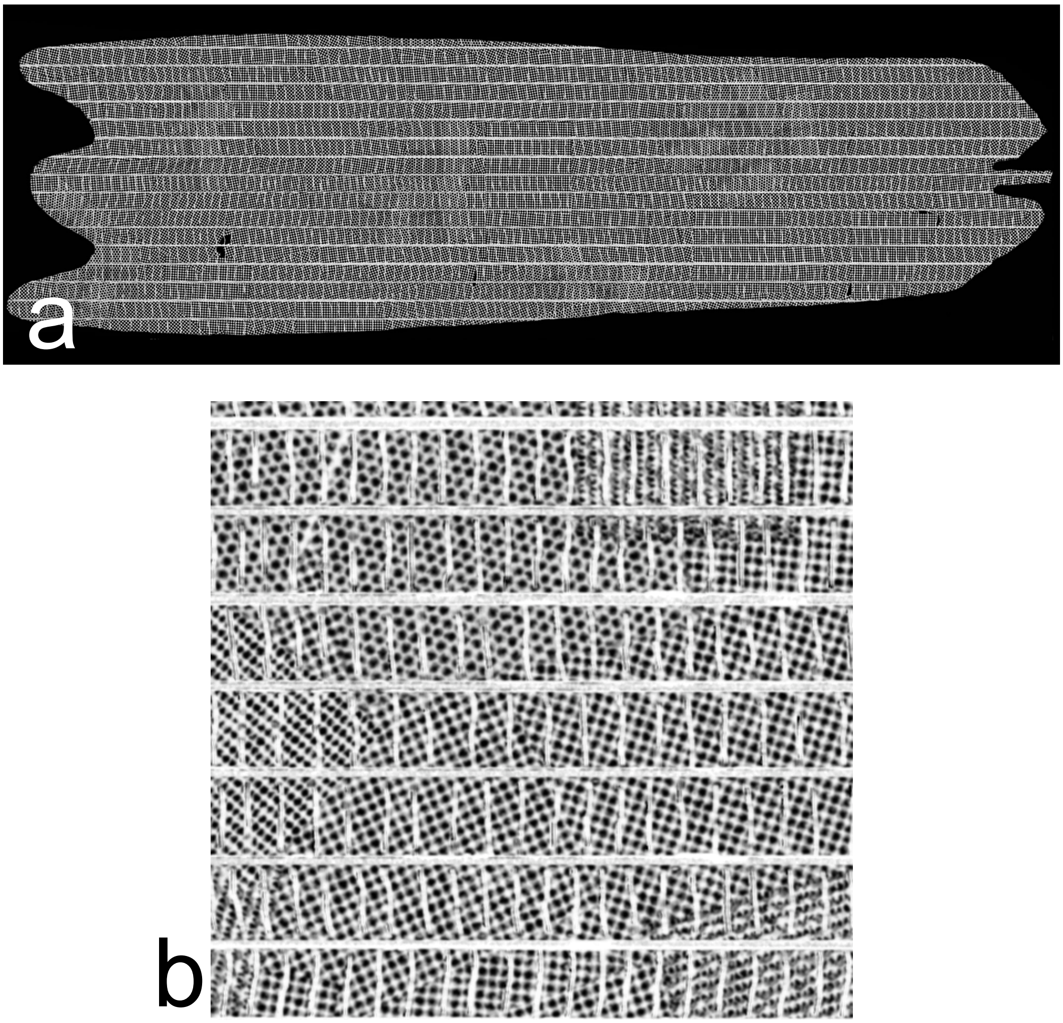

***Figure S11 (a)** Simulated whole wing scale containing projected images of ribs, cross ribs and variously oriented single gyroid domains. **(b)** Selected area from (a) above showing projections of ribs, cross ribs and simulated domains oriented along <100>; <110>; <111> and <311>. The left side of Figure 2a in the main text shows the FFT of the simulated scale.*

Section 4. Domain orientation and probabilities

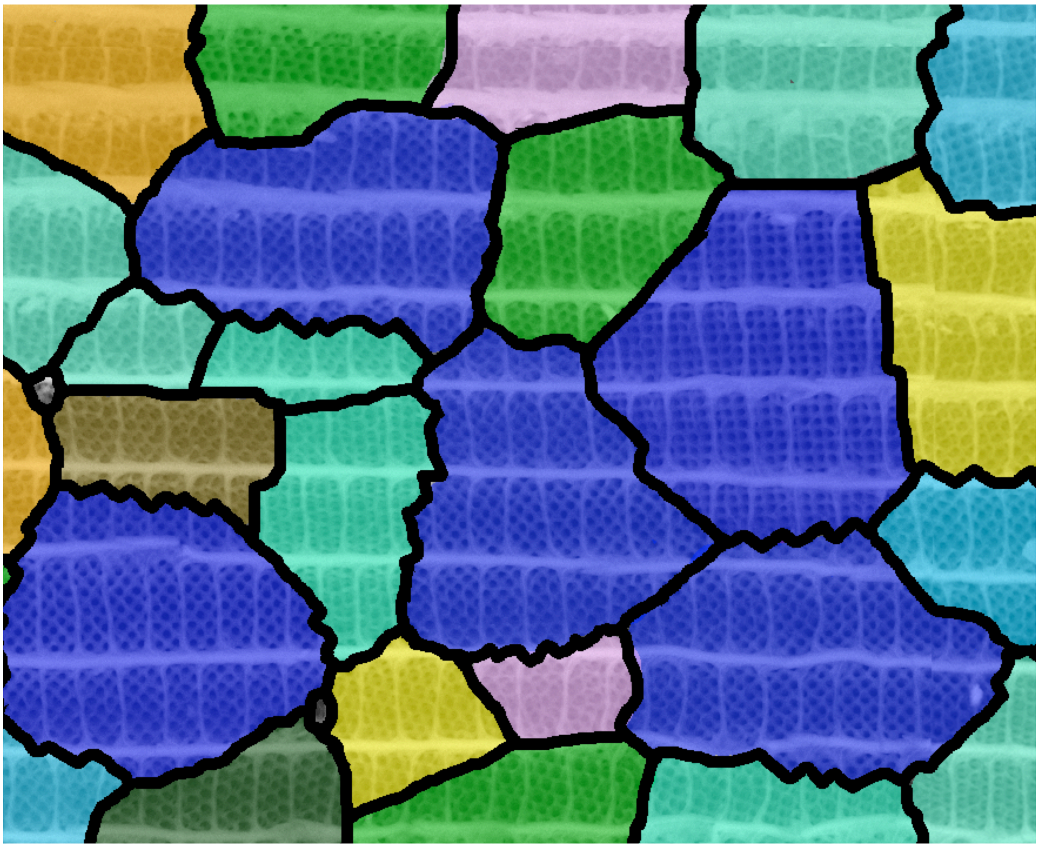

Figure S12 showing close up view of domains and their boundaries overlaid on the SEM image from which they were derived. Each domain is colour-coded according to its assigned orientation. Colour-code: dark/light blue - on/off axis <100>; cyan/orange – on/off axis <311>; bright/dark green – on/off axis <111>; yellow/brown – on/off axis <110>; pink – other (disordered/unidentified). Each dark blue <100> oriented domain is ca. 6 μm wide.

Area distributions

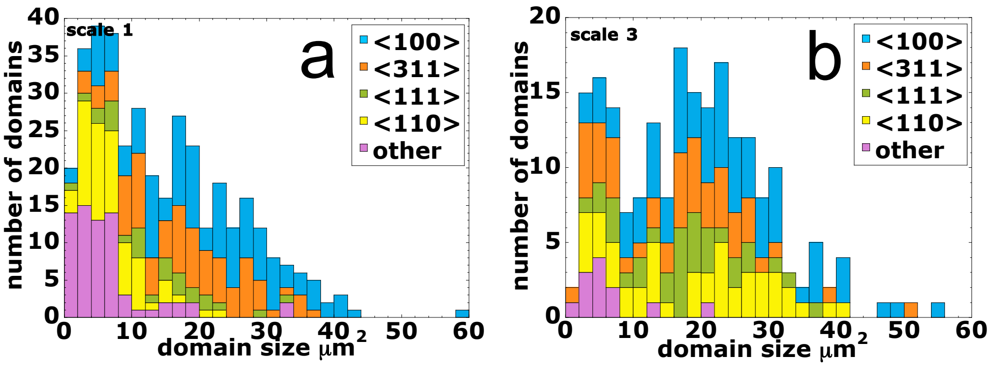

Figure S13. *Area distribution histograms corresponding to the domains of different orientation found in wing scales demoted scale 1 (a) and scale 3 (b). Note the higher proportion of relatively larger <110> domains in scale 3. Also see Figure 4 in the main text.*

Calculation of the probability of domain orientation

Each crystallographic orientation maybe represented by its pole, i.e., the projection of its director onto a sphere. The probability a domain will be oriented in a certain direction at random was therefore calculated as the area percent coverage of the sphere by a spherical cap with radius 5, 10 or 15°.

For example, there are a total of six <100> poles, four spaced 90° apart along the equator and two at the poles. Therefore the chance of occurring within say 10° of any of the six the <100> is equal to the % coverage of a single 10° cap multiplied by 6.

Similarly we can calculate the probability if the domain orientations are random but restricted to a band encircling the sphere determined by rotation around any <110> axis. The probability was therefore approximated by calculating the area of a square spherical cap with side 10, 15 or 20° and again multiplying by the number of poles for that set of <hkl> orientations.

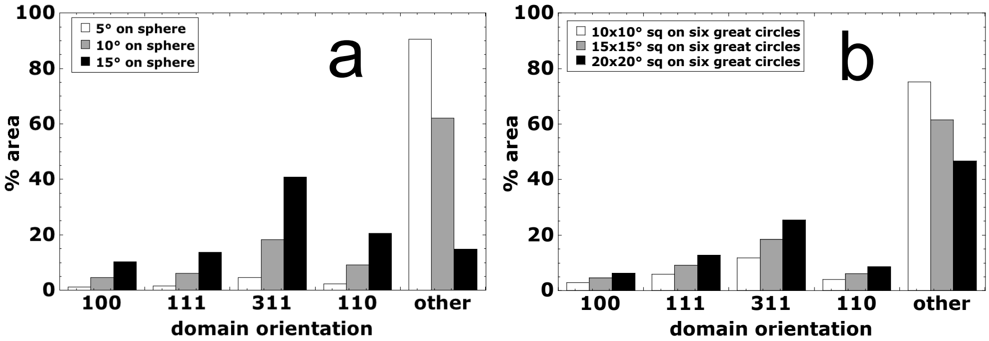

Figure S14 *Calculated probabilities (%) of a domain being oriented along a certain crystallographic direction (a) assuming that the there is no bias and (b) assuming a bias along the great circle bands defined by the traces of the <110> crystallographic poles. See main text for a discussion on this point, which is related to the fact that all observed crystallographic orientations namely the <100>, <111>, <311> and <110> can be rotated onto each other using a single rotation around a <110 > axis.*

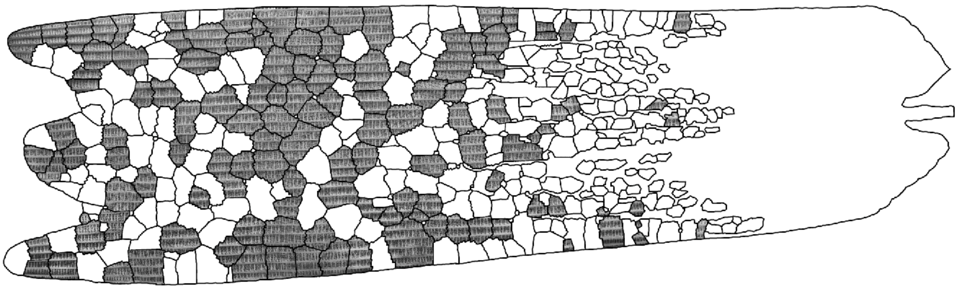

Figure S15 *Masked SEM image also showing domain boundaries of the wing scale denoted as scale 1. Masks were used to determine the relative area of domains of interest, for performing FFTs on selected areas of the SEM images and for obtaining colour information from selected optical images overlain in register with the SEM images. See main text for a discussion of the use of masks.*

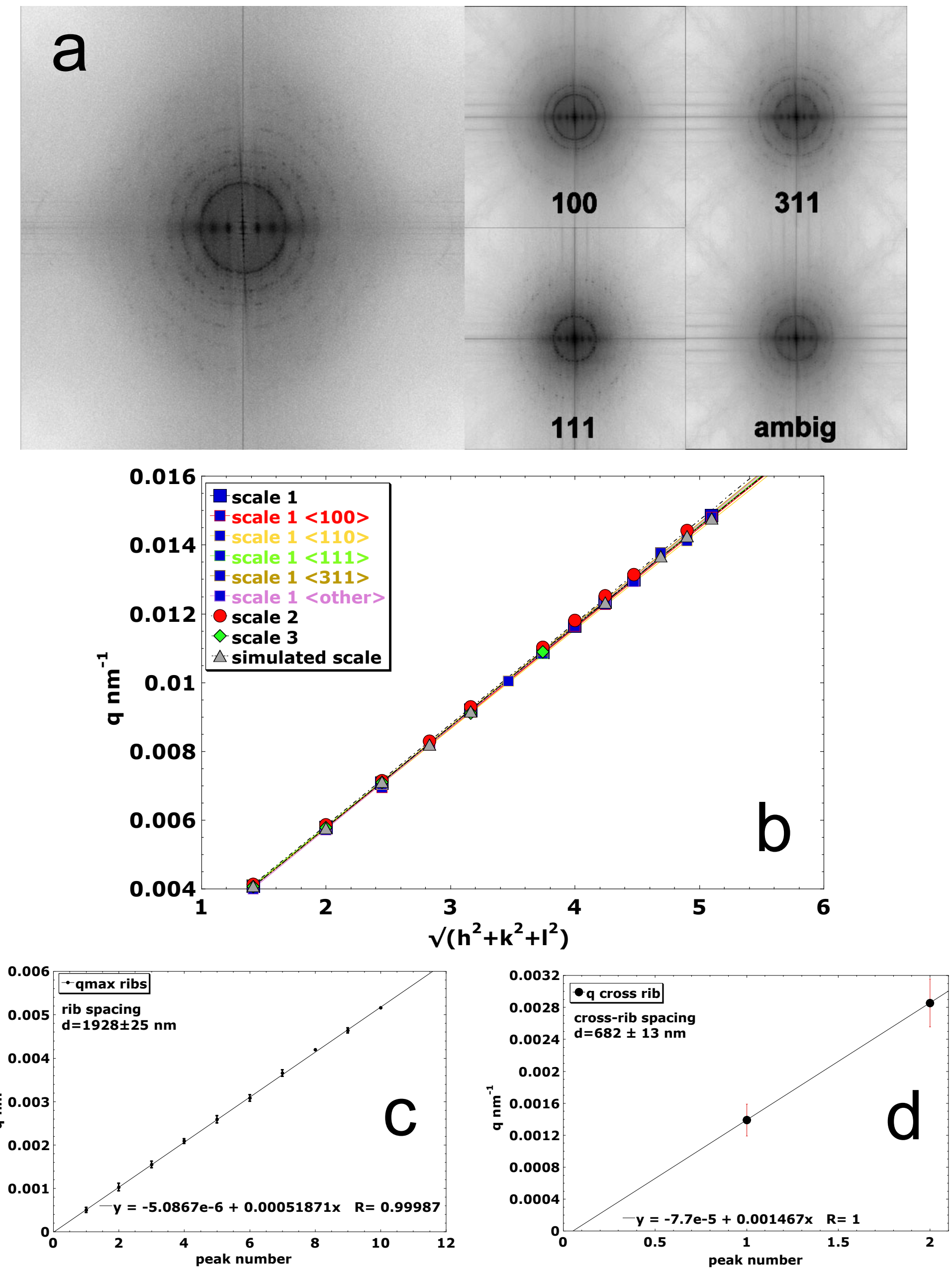

Figure S16 (a) *2D FFTs of unmasked (left) and selected masked areas of scale 1 (right),* ***(b)*** *Linear fits to plots of peaks found in radially integrated FFTs (see Figure 5 of main text) obtained from selectively masked areas within high resolution SEM mosaics of the various wing scales. The inverse slope of the linear fits yields the average lattice constant for the particular data set (see Table S2 below).* **(c,d)** as for (b), but with an azimuthal mask applied over the 2D FFT to highlight the ribs (c) and cross-ribs (d).

Table S2. *Results of linear regression fits to FFT data for unmasked scales 1-3, masked scale 1 and simulated scale.*

scale	masking	q nm^{-1}	d nm	std. dev.
**scale 1**	none	0.00292	342.2	1.0
**scale 1**	<100>	0.00291	343.5	2.7
**scale 1**	<110>	0.00289	346.5	1.0
**scale 1**	<111>	0.00292	342.8	1.4
**scale 1**	<311>	0.00295	338.9	2.2
**scale 1**	other	0.00295	338.9	4.5
**scale 2**	none	0.00296	338.0	1.2
**scale 3**	none	0.00292	342.8	1.4
**simulated**	none	0.00291	343.1	1.2

Section 5. Correlation between scattering angles and crystal structure

Table S3 *Optical objectives used in this study. Zeiss objectives were used to obtain the spectral data, while Leica objectives were used in the optical imaging.*

manufacturer	numerical aperture (NA)	NA angle °	magnification x
**Leica**	0.12	6.9	5
**Zeiss**	0.13	7.5	5
**Zeiss**	0.22	12.7	20
**Leica**	0.25	14.5	10
**Leica**	0.4	23.6	20
**Zeiss**	0.55	33.4	50
**Zeiss**	0.7	44.4	100
**Leica**	0.75	48.6	50
**Leica**	0.85	58.2	100

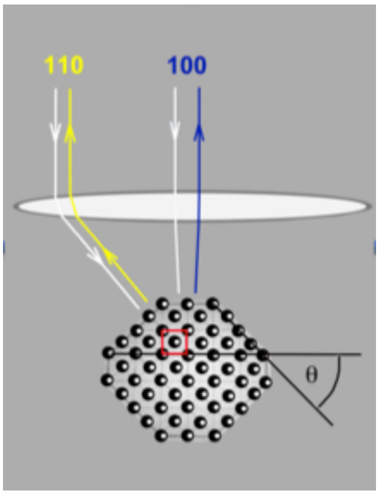

Figure S17*. Simple schematic model for the iridescence of <100> oriented domains at low and high NA imaging. At low NA, incident and reflected light 'sees' only the scattering arising parallel to the optic axis, in this case coinciding with the <100> crystallographic axis. At a larger NA corresponding to an angle of incidence/reflection of 45°, a <110> crystallographic axis becomes normal to some of the incoming/returning light. At NAs corresponding to > 45° the photonic response in both the <100> and <110> crystallographic directions will become mixed, thus explaining the iridescence observed for <100> oriented domains.*

	<100>			<110>		
	Tilt angle °	Miller index	# Azimuth °	Tilt angle °	Miller index	# Azimuth °
<100>				**45**	**[110]**<br>$\mathbf{[1\bar{1}0]}$<br>**[101]**<br>$\mathbf{[10\bar{1}]}$	**0**<br>**90**<br>**180**<br>**270**
<100>	90	[010]<br>$[00\bar{1}]$<br>$[0\bar{1}0]$*<br>[001]*	0<br>90<br>180*<br>270*	90	$[01\bar{1}]$<br>$[0\bar{1}\ \bar{1}]$<br>$[0\bar{1}1]$*<br>[011]*	45<br>135<br>225*<br>315*
<110>	**45**	**[010]**<br>**[100]**	**0**<br>**180**			
<110>				60	$[01\bar{1}]$<br>$[10\bar{1}]$<br>[101]<br>[011]	60<br>120<br>240<br>300
<110>	90	$[00\bar{1}]$<br>[001]*	90<br>270*	90	$[\bar{1}10]$<br>$[1\bar{1}0]$*	0<br>180*
<111>				**35.26**	**[110]**<br>**[101]**<br>**[011]**	**90**<br>**210**<br>**330**
<111>	**54.74**	**[010]**<br>**[100]**<br>**[001]**	**30**<br>**150**<br>**270**			
<111>				90	$[\bar{1}10]$<br>$[01\bar{1}]$<br>$[10\bar{1}]$<br>$[1\bar{1}0]$*<br>$[0\bar{1}1]$*<br>$[\bar{1}01]$*	0<br>60<br>120<br>180*<br>240*<br>300*
<311>	**25.24**	**[100]**	**ca. 130**			
<311>				**31.48**	**[110]**<br>**[101]**	**ca. 30**<br>**ca. 245**
<311>				64.76	$[10\bar{1}]$<br>$[1\bar{1}0]$<br>[011]	ca. 105<br>ca. 172<br>ca, 320
<311>				90	$[01\bar{1}]$<br>$[0\bar{1}1]$	ca. 48<br>ca. 228

** redundant orientation; # relative to the azimuth of the [001] orientation fixed at 270°.* ***Bold:*** *Crystallographic orientations with tilt angles in the range of current optical experiments*

Table S4 *above shows the tilt angles (or inclinations) of <100> and <110> family of crystallographic orientations relative to the observed orientations <100>, <110>, <111>, <311> of photonic crystal domains seen in the wing scales of the C. rubi specimens studied here. These were calculated using the dot product of the vectors corresponding to the normal of their respective Miller planes. The orientations are named using Miller indices of crystallographic orienations [hkl] that are normal to the optic axis when the wing scales are lying flat with the ribs up.*

The angle between various crystallographic orientations in Table S4 were calculated using equation 1 below:

$$\theta = \cos^{-1}\left(\frac{\boldsymbol{a}\cdot\boldsymbol{b}}{\|\boldsymbol{a}\|\|\boldsymbol{b}\|}\right) \tag{1}$$

where $\boldsymbol{a}$ and $\boldsymbol{b}$ are vectors corresponding to the normals of the respective Miller planes. For example the angle between the normals to the (111) and (110) crystallographic planes are the vectors:

$$\boldsymbol{a}= \begin{bmatrix}1\\1\\1\end{bmatrix} \text{ and } \boldsymbol{b}= \begin{bmatrix}1\\1\\0\end{bmatrix}$$

Now,

$$\boldsymbol{a}\cdot\boldsymbol{b} = 1+1+0 = 2,$$

and,

$$\|\boldsymbol{a}\|\|\boldsymbol{b}\| = \sqrt{(\mathbf{1}^2+\mathbf{1}^2+\mathbf{1}^2)}\times\sqrt{(\mathbf{1}^2+\mathbf{1}^2+\mathbf{0}^2)} = \sqrt{\mathbf{3}}\times\sqrt{\mathbf{2}} = \sqrt{\mathbf{6}}$$

Therefore,

$$\theta = \cos^{-1}\left(\frac{\mathbf{2}}{\sqrt{\mathbf{6}}}\right) \sim 35.26°$$

If $\boldsymbol{a}\cdot\boldsymbol{b} = 0$, then $\theta = 90°$, and the vectors (plus their corresponding Miller planes) are orthogonal.

Section 6. Spectral Response

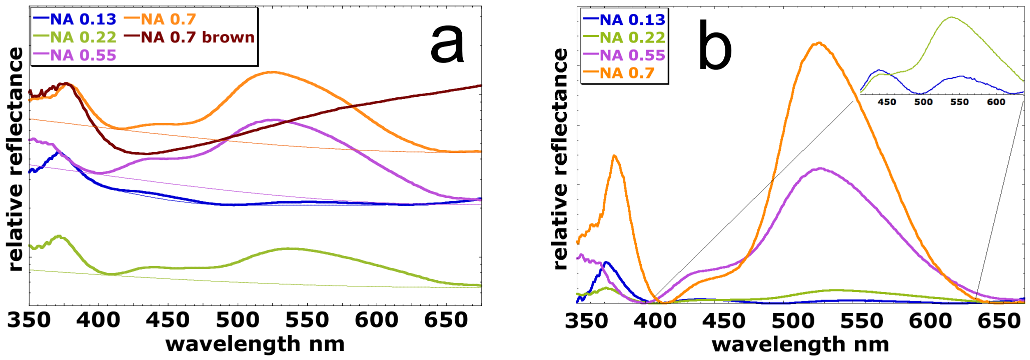

***Figure S18 (a)** Optical reflection raw spectra of a single scale obtained by the handheld spectrometer (see section 2.6 of the main text) and the parabolic backgrounds used for subtraction to obtain the spectra shown in **(b).** Figure 7a in the main text shows the same data normalized by intensity of the peak near 520 nm. For the spectrum collected with a NA 0.13 objective, segments of two parabolic curves were used as a background. The backgrounds are current best estimates of the background chitin-containing material based on typical spectra from non-photonic wing scales (see for example NA 0.7 brown spectra in (a) obtained from brown scales with no photonic crystal response).*

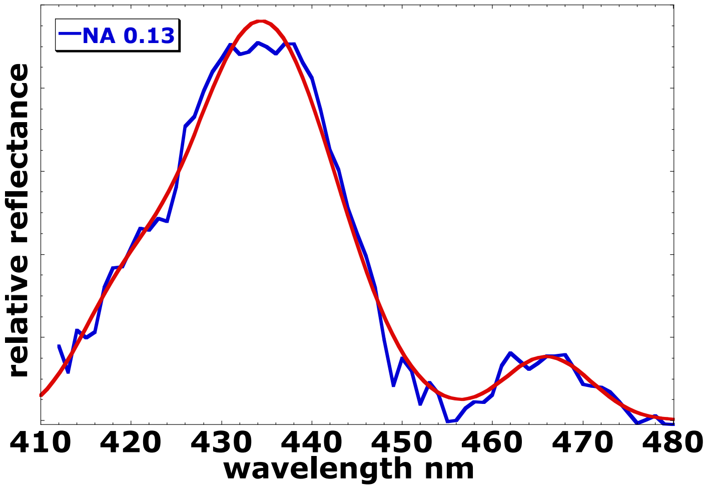

***Figure S19.** Spectra from C. rubi at NA 0.13 showing the blue peak arising from scattering of <100> oriented domains, and a smaller peak attributed to cyan scattering from <111> aligned domains. The peaks are fitted with two Gaussians. See section 2.6 in main text for experimental details.*

Table S5 *Wavelengths of observed spectral maxima for C. rubi**

	**NA 0.13**		**NA 0.22**		**NA 0.55**		**NA 0.7**	
	peak1	peak2	peak 1	peak2	peak 1	peak2	peak 1	peak2
**λ**	443.5	554.2	437.9	548.0	435.2	523.4	446.5	522.1
**σ**	18.5	29.0	21.1	46.3	13.7	33.3	14.7	26.1

*Based on Gaussian fits to the spectrometric peaks

Table S6 *Hue-based wavelengths determined for wing scale 3 (C. rubi)**

	**NA 0.12**		**NA 0.25**		**NA 0.4**		**NA 0.75**		**NA 0.85**	
	λ	σ	λ	σ	λ	σ	λ	σ	λ	σ
**100**	449.6	5.1	476.4	7.5	485.7	4.8	522.4	8.1	525.9	8
**111**	460.4	16.4	494.0	7.3	497.8	6.9	526.1	8.1	527.2	6.7
**311**	458.3	8.3	486.6	17.6	493.7	5.2	520.6	10.5	523.2	9.6
**110**	550.8	8.1†	547.9*	6.3	543.8	7.1	526.2	12.4	525.5	3.5

*Based on Gaussian fits to the peaks using optical microscopy images (hue-based).

Colour filter calibration for extraction of pseudospectral data from images

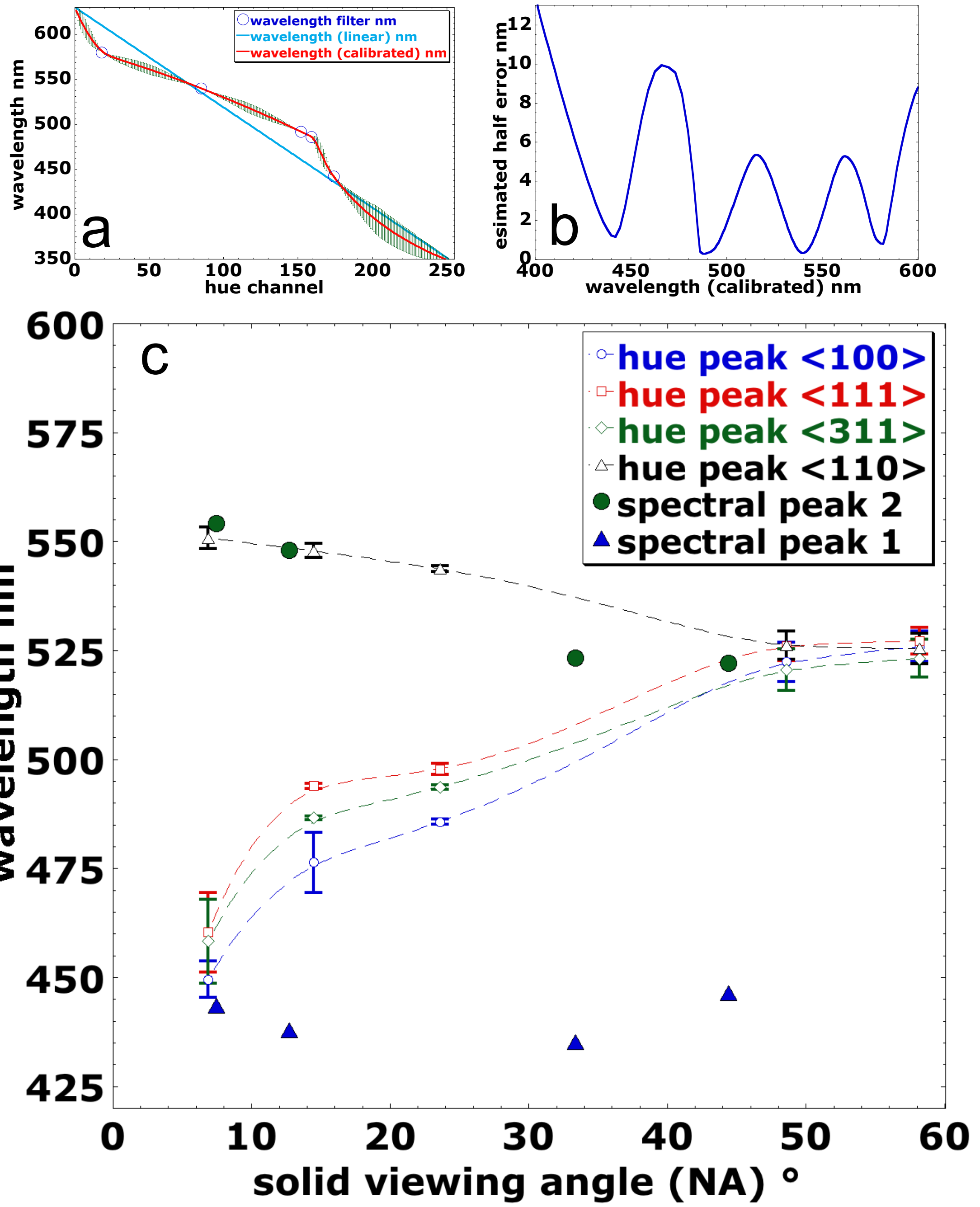

Figure S20 *(a) RGB-based hue to wavelength conversion. Note the sharp change in slope of the interpolated curve in the hue channels between approximately 160 and 175, which represents 44 nm in wavelength space or about 3 nm per channel. The exact shape of the camera response function in this range is subject to a larger uncertainty, which reflects in the error of the estimated wavelength (i.e. ± 10 nm at 465 nm based on the range of possible envelope curve shapes). However, the uncertainty rapidly reduces to ca. ± 0.7-1.5 nm (half the hue channel spacing) at the wavelengths of the colour filters. (b) An estimate of the error function in the calibration curve is given as a function of the distance between filters defined by some simple envelopes that are symmetric around the central interpolated fit curve. (c) Iridescence as a function of solid viewing angle for variously oriented domains in wing scale 3. Open symbols are image-based data and solid circles are from spectrometric measurements. Dashed lines are interpolated fits to the image-based data as a guide to the eye only.*

Each of the four curves in Figure S20c can be understood with reference to the stereographic projection in Figure 9a. For example, take the curve representing the reflected integrated colour from <100> oriented domains shown in blue in Figure S20c – the first point corresponds to NA 6.9°. Now in Figure 9a an NA of 6.9° is just under half the radius of the first small circle surrounding the point representing a <100> pole. Integrating inside this circle will give an average RGB value that can be equated to the blue hue plotted at lowest NA in Figure S20c. Also referring to Figure 9a, we can see that at NA of 15° (the second point in Figure S20c), integrated colour is now red-shifted and so on. By almost 60° NA, equivalent to the highest NA objective used, one can easily see (again with respect to Figure 9a) why the scattering from <100> oriented domains has moved to an average green hue. A similar argument is easily then made for domains corresponding to the other observed orientations. To understand the spectral behavior it is useful to recall explicitly that in contrast to the hue-based data, the spectral data also contains intensity information.

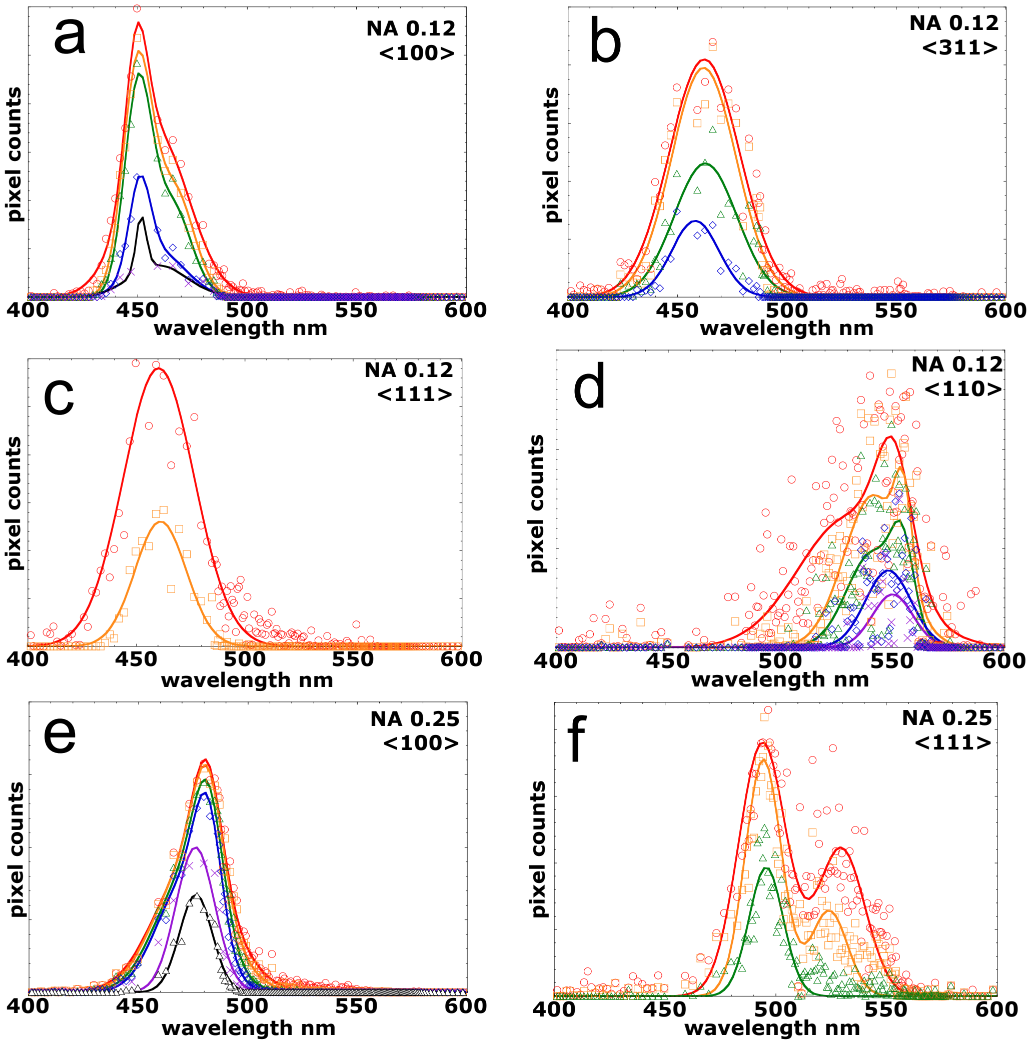

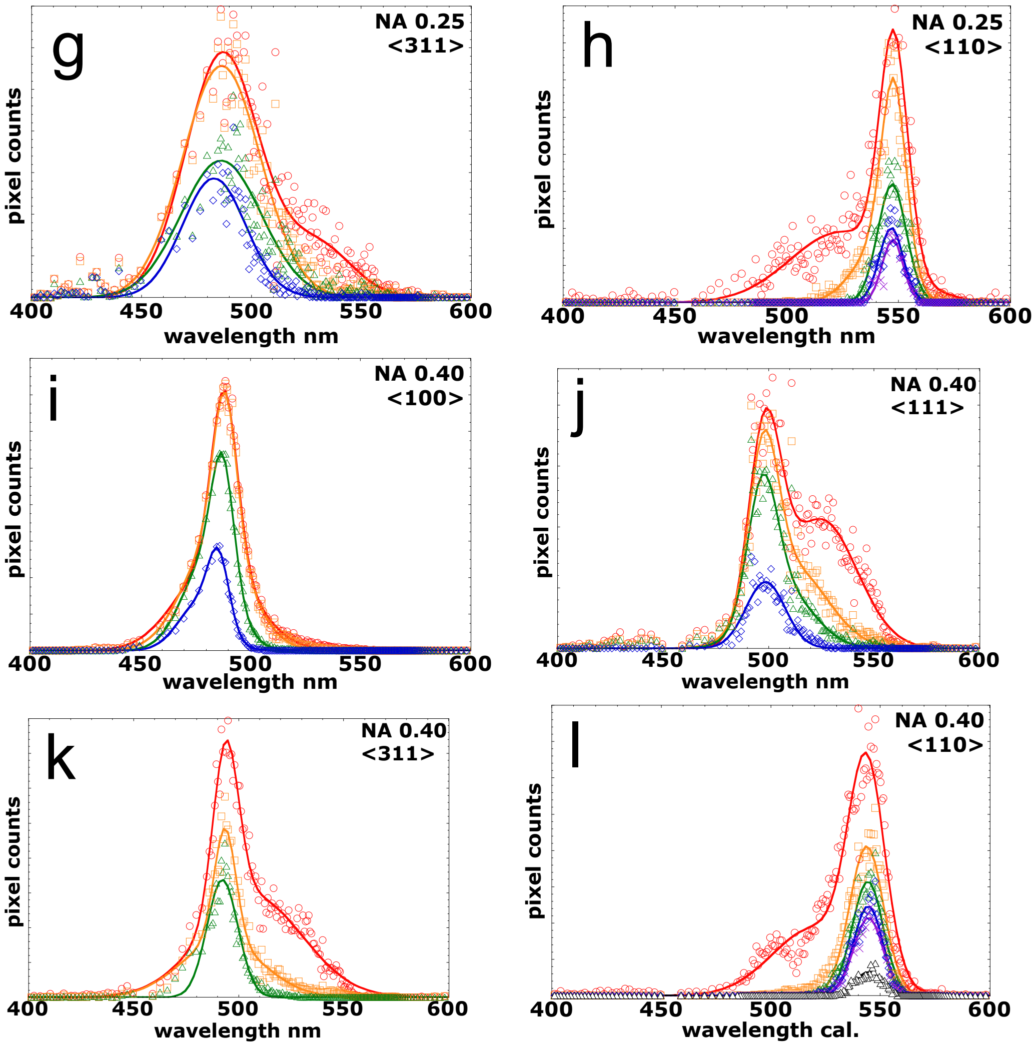

Figure S21(a-l) *Wavelength calibrated hue-histograms obtained from scale 3 at relatively low NA values (i.e., 0.12, 0.25 and 0.40) using masks for the various orientations. In each graph, the separate curves represent increasingly smaller area masks designed to decrease the effects of defects and scattering 'bleed-over' from neighbouring domains due to being near the resolution limit. This process has also selected domains with higher degrees of orientational alignment and crystallographic order, as judged by detailed inspection of each domain in high-resolution SEM images. The curves were fit with mono-, bi- and trimodal Gaussian fits, with each successive decrease in mask area better defining the primitive scattering from the best quality and most oriented domains of that particular orientation. It is clear that the bulk of the scattering in the minimally masked curves arises at the same wavelength as the maximally masked curves, strong evidence that the scattering from a particular orientation is centered at a particular wavelength.*

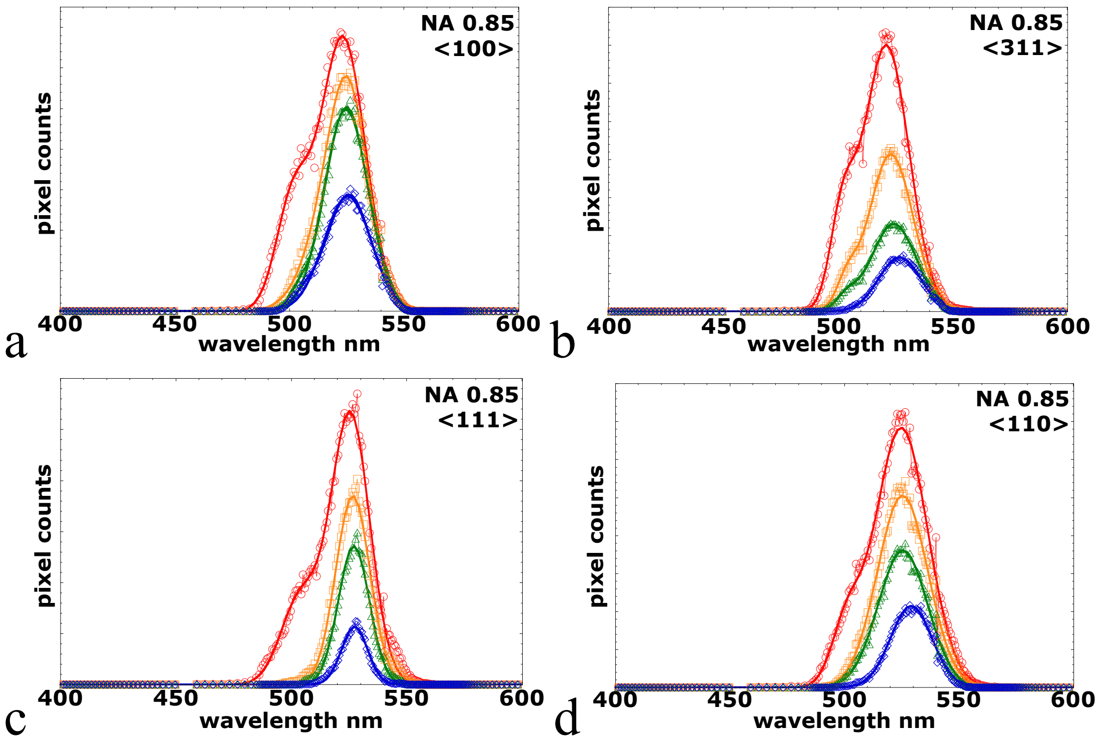

Figure S22a-d. *Wavelength calibrated hue-histograms obtained from C. rubi at NA 0.85, showing the convergence to scattering at a single wavelength near 520 nm.*

Section 7. Modeling of photonic response

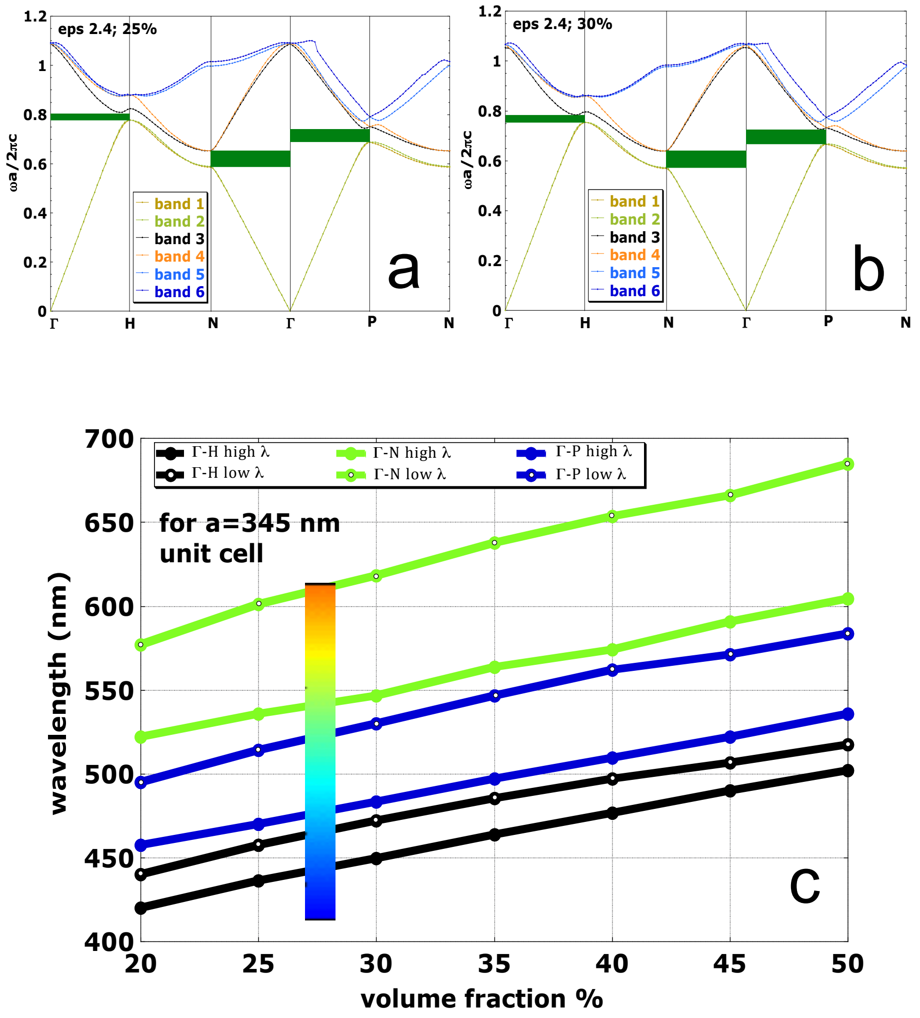

Figure S23 *Band diagrams of (a) 25% and (b) 30% volume fraction single-gyroid structures calculated using MPB. The gyroid was constructed as a trigonometric approximation with a level cut defined in table S5 below and using a dielectric constant of 2.4 for chitin. (c) Band gap map constructed from multiple runs of MPB while varying only the volume fraction of chitin for a slightly different dielectric constant (2.6).*

Table S7 *Conversion table. Level cut parameter (z) and corresponding volume fractions of the trigonometric approximation to the gyroid minimal surface.*

Volume fraction %	t
15.00	0.53
20.00	0.46
25.00	0.38
30.00	0.31
35.00	0.23
40.00	0.15
45.00	0.08
50.00	0.00
55.00	-0.08
60.00	-0.15
65.00	-0.23
70.00	-0.31
75.00	-0.38
80.00	-0.46
85.00	-0.53

The nodal surface or trigonometric approximation[1] used to approximate the gyroid was:

$$\sin x.\cos y + \sin y.\cos z + \sin z.\cos x = t$$

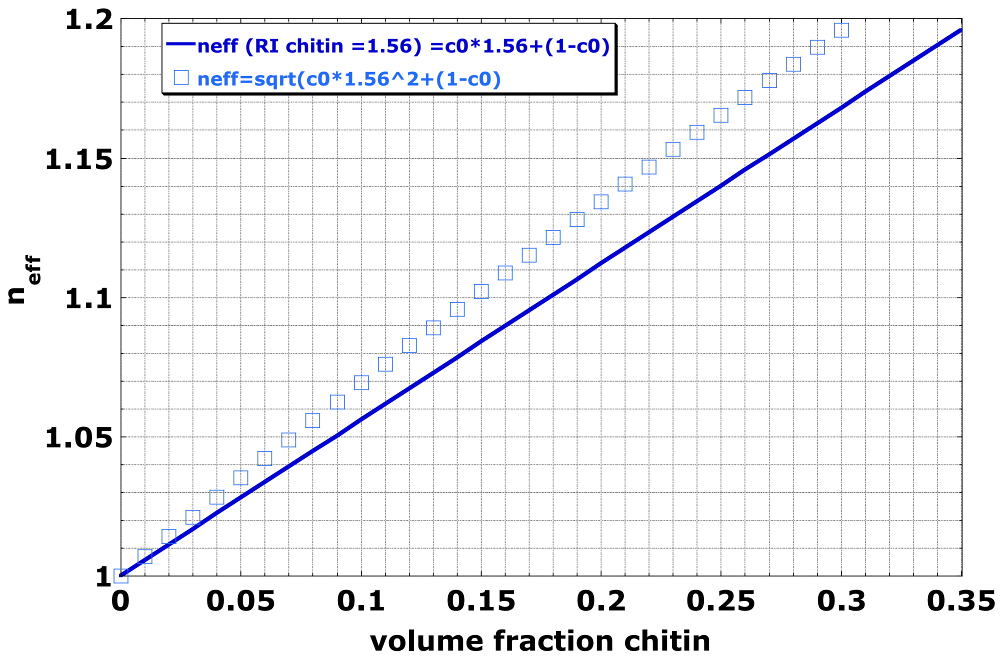

Figure S24 *Effective refractive index as a function of volume fraction using a refractive index for chitin of 1.56. The linear curve is based on a parallel mean field approximation, whereas the dotted line represents a Drude mean field approximation. The former approximation is used in this study.*

Section 8. Determination of orientation distributions from colour images.

Images of wing scales taken without polarisers at NA 0.12-0.4 were manually segmented using the Photoshop Magic Wand tool into separate colour files. The tool was used non-contiguously at a tolerance of 10, gradually adding picking larger areas of similar colour and brightness by repeatedly enlarging the selection in the order given below. Repeated efforts showed the images could be accurately demarcated into the following categories:

1. Bright yellow greens (YG). These were discrete and accurately demarcated.

2. Dark green edges (GE). These topologically surrounded YG areas. These form a very small amount and are considered to arise from optical effects spilling over from <110> domains predominantly onto <111> and <311> domains.

3. Bright to intermediate blues (BL). These were very well defined with brighter blues forming discrete areas. These arose mostly from the <100> oriented domains, with a few percent contributed from other orientations.

4. Dark cyans. (CY) These are also relatively well defined (see the hue histogram) arising mainly from <311> and also <100> and <111> oriented domains.

5. Dark greens. (DG) These are also relatively well defined and arise from equal contributions from <311> and <111> oriented domains.

Segmented images were divided into separate colour layers for each of the categories above. The hue histograms of the individual layers thus were used as a vector basis set for reconstructing the relative distribution of the four identified orientations. The aim was to take the basis set and find the weighted contributions from each colour layer that most accurately reproduces the observed hue histograms obtained for each orientation (see the figure S25 below).

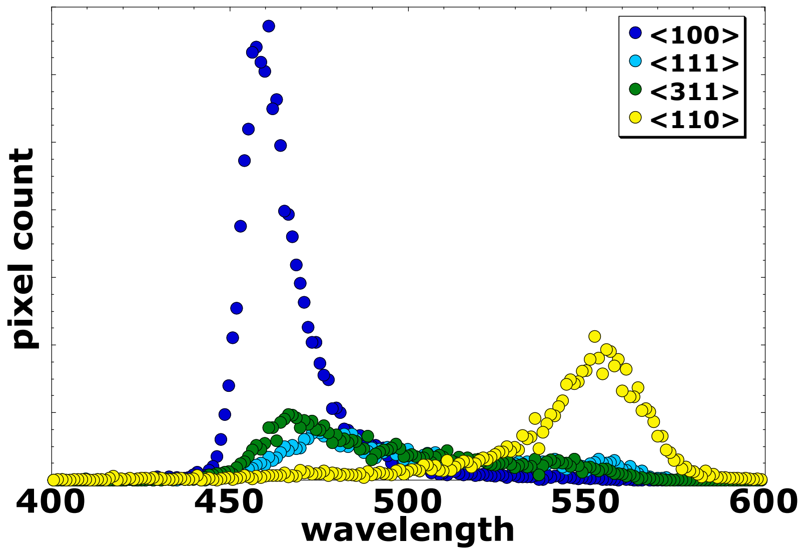

Figure S25. *Hue histograms indicating the observed colours found by masking relevant areas of a representative NA 0.40 colour optical microscope image overlaid on the structural domain map obtained using SEM mapping discussed in the main text.*

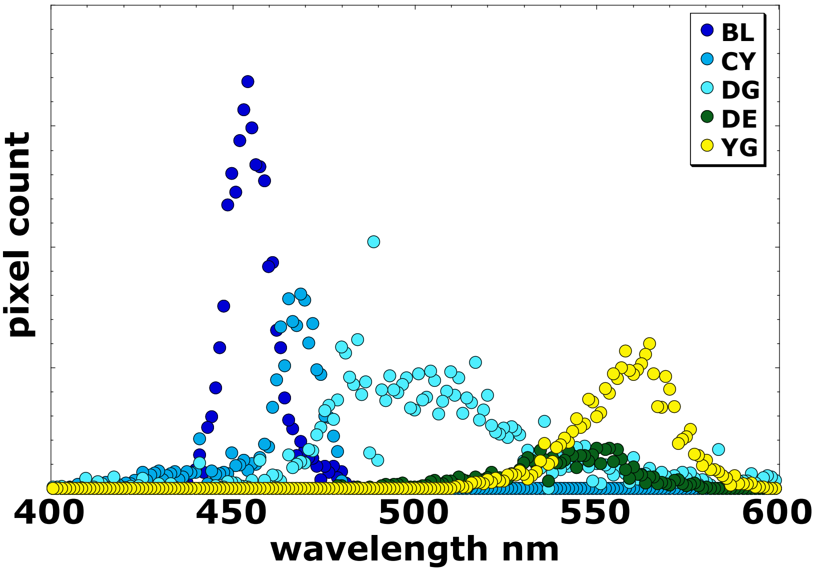

Figure S26. *Hue histogram for each of the segmented layers of the same NA =0.40 optical microscope image used to obtain Figure S25.*

As is apparent from a quick visual comparison, the blues (*ca.* 450 nm) and yellows-greens (*ca.* 560 nm) already give a very accurate indication of the relative distribution of the <100> and <110> orientated domains (recall the areas under the curves are the relative areas).

From comparing these two graphs, it is obvious that the mid-wavelength colours (dark cyans (*ca.* 470nm) and dark greens (*ca.* 470-540 nm) do not arise from any one orientation. Assuming a linearly independent basis set with five basis vectors, the following weighted vectors were found to most accurately reproduce the observed hue histograms for the respective orientations:

$$\begin{pmatrix} [100] \\ [111] \\ [311] \\ [110] \end{pmatrix}_n = \begin{bmatrix} 0.93 & 0.65 & 0.1 & 0 & 0 \\ 0.02 & 0.1 & 0.4 & 0.4 & 0 \\ 0.05 & 0.25 & 0.4 & 0.4 & 0 \\ 0 & 0 & 0.1 & 0.2 & 1 \end{bmatrix} \begin{pmatrix} \mathrm{BL} \\ \mathrm{CY} \\ DG \\ GE \\ YG \end{pmatrix}_n$$

where *n* is the hue channel number (0-255).

The resulting histograms after applying this transform are below. The magnitudes of the matrix elements were determined by manual trial and error.

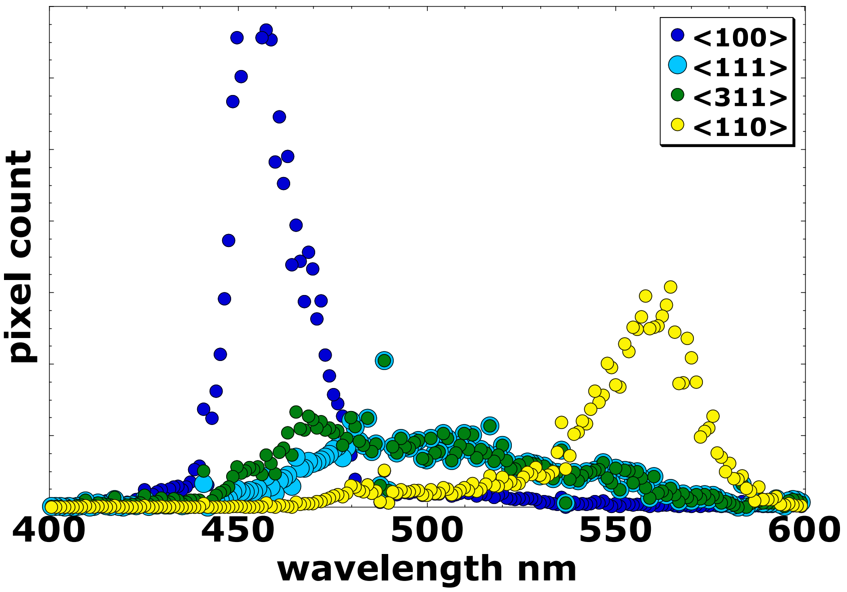

***Figure S27**. Hue histograms for each orientation determined from NA 0.40 optical imaging and trial and error fitting of the matrix elements in the equation above. It is a remarkable good fit when compared with Figure S25 above.*

A comparison of the relative areas determined from the SEM imaging and the colour segmentation method can now be made. With a reasonable fit of the colour segmentation areas to those determined from the SEM imaging, it was then possible to estimate domain orientations from larger area optical images, an otherwise prohibitive task using regular, non-automated SEM imaging.

So given a reasonable transformation matrix to operate on the segmented hues to give the orientation distributions, one can apply this to another image containing multiple scales to get an idea if a similar distribution of orientations occurs over a larger number of scales. The ability to differentiate the relative contributions of <311> and <111> domains by segmenting colour images is less certain than for those of <100> and <110> oriented domains, due to their relatively similar scattering profiles in imaging as evidenced in the profiles shown in Figure S25. The profiles of the <100> and <110> oriented domains are quite distinct in this respect, and thus are thus less prone to errors in the colour segmentation process.

Consequently, an image taken at NA 0.25 containing approximately 90 scales was segmented as described earlier into five channels, BL, CY, DG, DE and YG. Pixels specified in a sixth category named 'OTHER' were included to account for the few pixels that did not fall into these categories. The percentage areas found upon segmentation appear in Table S8 below.

Table S8. *Areas of the respective segmentation colour categories determined from one run of the segmentation process applied to multiple scales within an NA 0.25 image of C. rubi.*

Segmentation category	Area% NA 0.25
**BL**	45.52
**CY**	11.71
**DG**	25.87
**DE**	7.83
**YG**	8.97
**OTH**	0.12

Several independent repeats of the segmentation process confirmed that areas could be obtained to within approx. ±5% in absolute area terms.

The transformation matrix (1) shown above then returns the following values when operating on the values given in Table S8:

Table S9. *Frequency of occurrence of domains of certain orientation detected optically for wing scale 3.*

orientation	Area % NA 0.12	Area % NA 0.25	Area % NA 0.40
**100**	45.8	52.440	38.6
**111**	18.3	15.560	17.7
**311**	20.4	18.680	20.8
**110**	15.5	13.120	22.8
**other**	0	0.12000	0

Images taken at NA 0.12, NA 0.25 and NA 0.40 were compared to assess which objective was best to perform image-based orientation analyses. Magnification using a NA 0.25, 10x objective offered the best combination of relatively low NA (to achieve good colour separation) and sufficient spatial resolution (to obtain well-defined colour domain boundaries coincident with those obtained by SEM structural mapping).

Section 9. Low NA, low magnification imaging of iridescence in *C. rubi*.

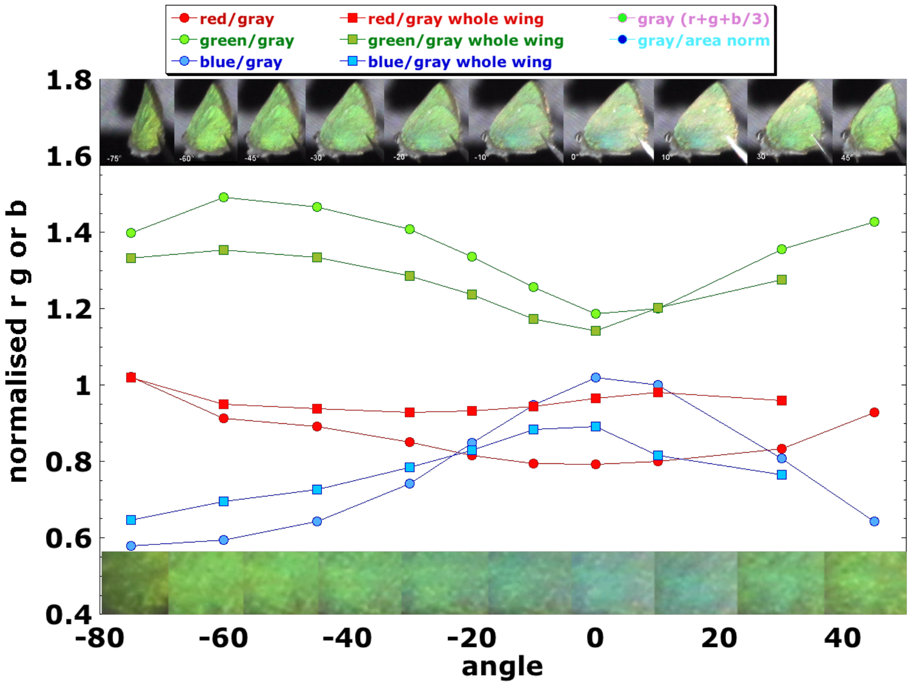

Figure S28. Low NA (<0.015) images of *C. rubi* at various angles. A telephoto lens with a 600 mm focal length was used at a distance of approximately 6 m to achieve an NA angle less than 1° and a total magnification of approximately 1x, well below the limit for resolving individual domains. A whole butterfly specimen was mounted on a rotating goniometer and imaged at different angles. Illumination was from an LED source immediately beside the camera at the same distance. The rgb channels were plotted separately to detect any iridescence. A slight blue shift is noted close to zero rotation angle where the wings were oriented normal to the optical axis of the system.

Section 10. Extension to other butterfly species.

Extension of the results for *C. rubi* to other related species also containing (or suspected of containing) single gyroid crystals.

***Chalbys (Thecla) hassan*:**

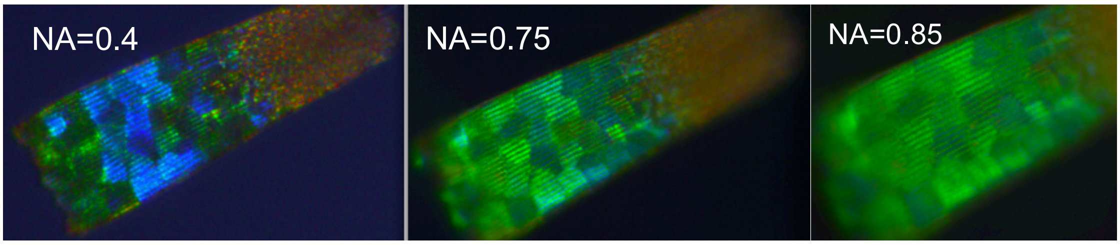

Figure S29. *Chalbys (Thecla) hassan scales at three different NAs (0.4, 0.75, and 0.85). The blue-green iridescence observed for the crystallites of this species is consistent with single gyroids oriented with a strong <100> orientation preference. Note the NA=0.4 colour change is due to a large curvature of the otherwise <100> oriented domains (assigned by analogy with C. rubi) the much larger size of crystallites in this species compared to all other gyroid-bearing species. These facts makes this species a particularly attractive for future studies of colours in butterflies arising from scattering from 3D photonic crystals. Scale width ca. 50 μm.*

Callophrys dumetorum:

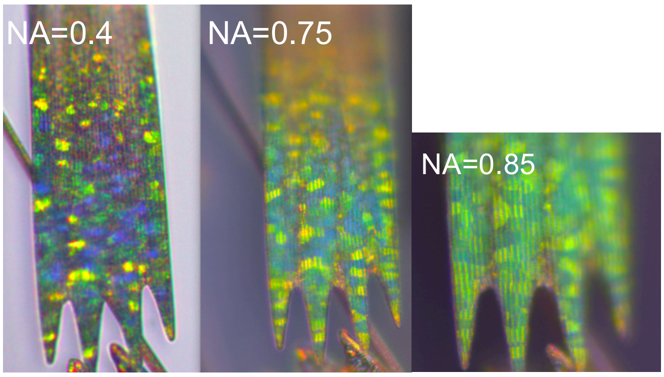

Figure S30 *Callophrys dumetorum scales at three different NAs (0.4, 0.75, and 0.85). Scale width ca. 50 μm.*

Callophrys (Thecla) acaste:

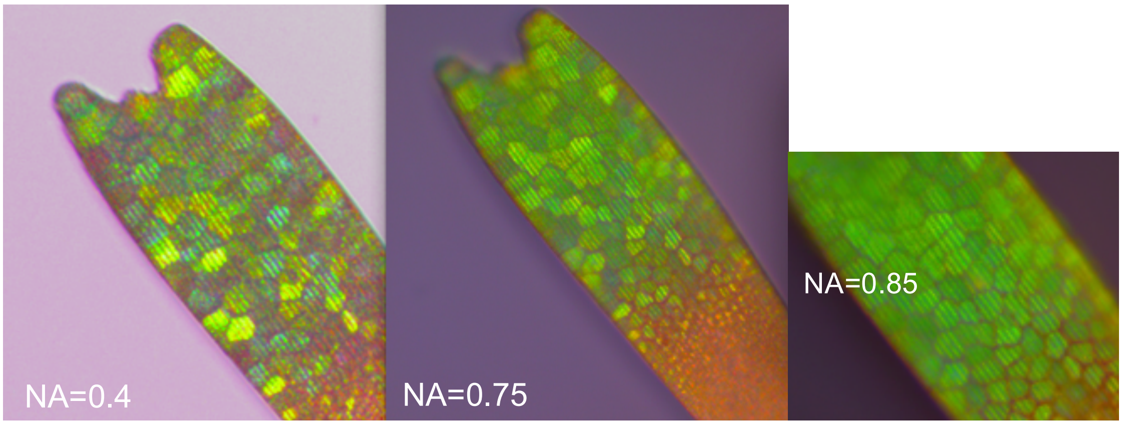

Figure S31 *Cyanophrys (Thecla) acaste (NA 0.4, 0.75, and 0.85). Scale width ca. 50 µm.*

Teinopalpus imperialis:

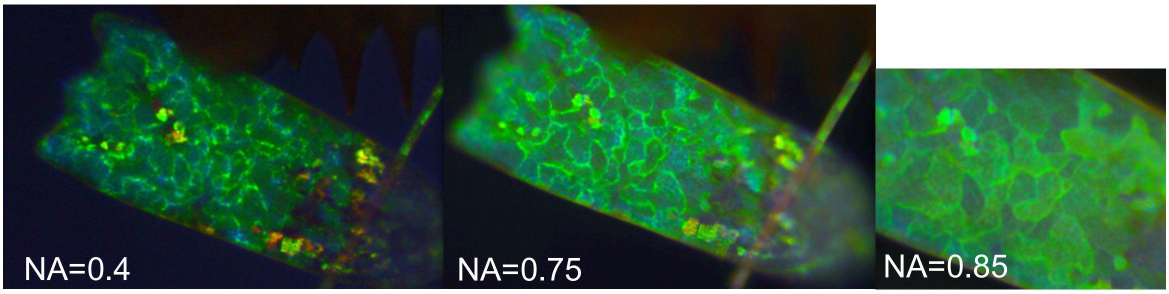

Figure S32 *Teinopalpus imperialis (crossed polarizers, NA 0.4, 0.75, and 0.85). Scale width ca. 50 µm.*

Parides sesostris:

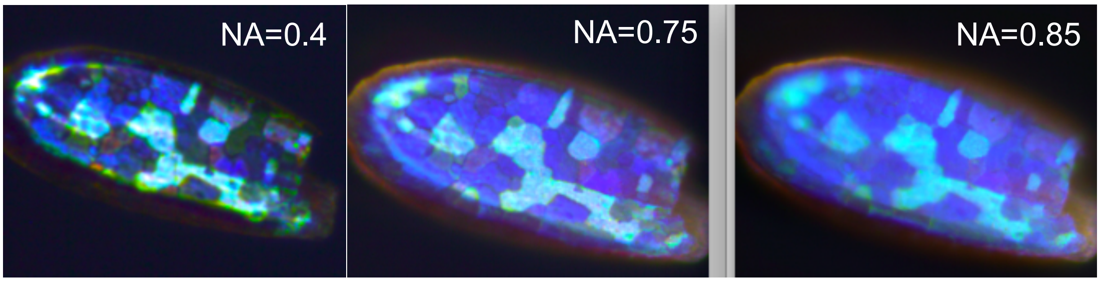

Figure S33 *Parides sesostris (reverse side of scale, crossed polarizers, NA 0.4, 0.75, and 0.85). Scale width ca. 50 µm.*

Section 11. Movie description

Movie S1. Simulated single-gyroid with 20% volume fraction with lighting to a depth similar to that seen in SEM images. Rotation is around a single <110> axis. This results in the following sequence of orientations parallel to the 'out of the page' view when rotated through 180° in 400 frames at a constant rotation rate:

Frame 001: <100>
Frame 56: <311>
Frame 122: <111>
Frame 200: <110>
Frame 277: <111>
Frame 343: <311>
Frame 400: <100>

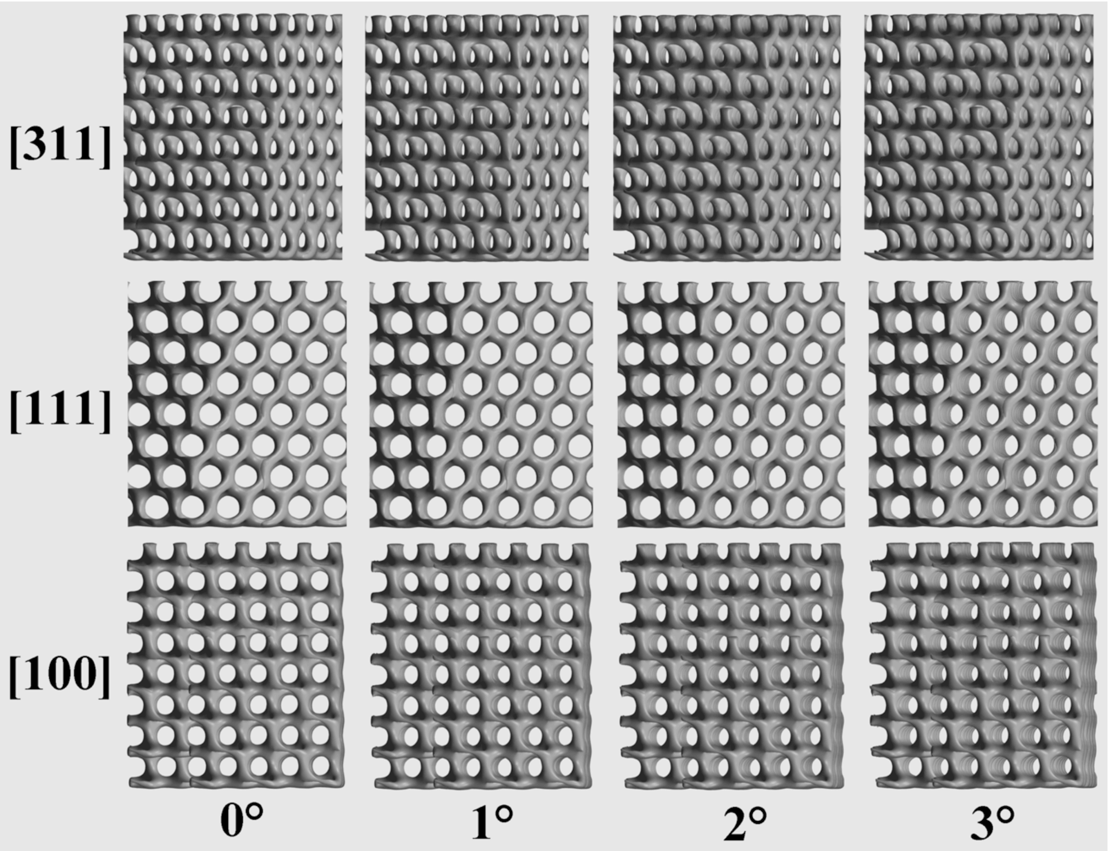

Figure S34. *Examples of simulated projections on and off axis in three of the four major axes. This illustrates that the symmetric alignment of tunnels can be quite a sensitive indicator for assessing (via SEM imaging) the degree of domain alignment along a major crystallographic axis.*

Section 12. Mapping of crystal domains: orientations.

The assignment of a particular domain orientation was performed entirely by visual inspection. The process is explained below. After the assignments were performed, the quality of these assignments was checked by analyzing the symmetry relations in their respective Fourier transforms. These checks were performed at the individual domain level for every domain in scale 1 to verify the process was reliable.

In assigning a crystal orientation to a domain, the least difficult were those domains with their respective <100> or <111> axes normal to the surface of the scale (see Figure S3a for an example). Under the conditions used for SEM imaging, it was relatively straight forward to see if the characteristic tunnels or holes of the single gyroid were also aligned at depth, particularly along the <100> and <111> directions, as the tunnels run straight through the structure from front to back in very characteristic arrays. Any misalignment of the tunnels necessarily manifested as a decrease in the size and circularity of the observed holes. Further, the inner 'walls' of the tunnels became visible at some depth into the structure if not in good alignment. This is a good example of how the 2D images provided a degree of 3D information.

The array of holes were square in the <100> direction or a hexagonal in the <111> direction. For the <311> and <110> directions, the holes also ran straight through the structure from front to back forming a characteristic symmetric pattern in each case. In these latter directions, the holes were narrow (elliptical) along one axis and were therefore more sensitive to tilt compared with the former directions. The <311> and <110> projections were readily distinguished from each other by the fact that the array of holes formed a 90° angle in the case of <110>, and an angle of 74° in the case of <311>.

We also simulated off-axis projections in order to see how quickly the symmetry relations changed beyond the point of being consistent with what was directly observed. In theory we could detect changes of less than 3°. However an uncertainty 5-10° was more likely due to image distortions associated with sample charging, variations in contrast or resolution and more obvious hindrances such as obscuration by the ribs or real distortions where the single gyroids fused to the ribs and cross ribs without preserving the underlying crystal symmetry.

Most domains in the three mapped wing scales were aligned on or near one of these four directions mentioned above and were each assigned accordingly.

Fourier transforming the images of individual domains gave a characteristic pattern of discrete peaks, shown, for example in Figure S35 and S36 right. In scale 1, more than 200 individual domains were transformed this way, and their reciprocal lattice vectors were checked against the expected reciprocal lattice parameters (see section S7). The typical observed angles and dimensions of the reciprocal space lattices were in good agreement with the calculated ones. A series of tilt images of simulated projections moving off axis are shown in Figure S34.

Assignment of <100> domains

Projection of a simulated single gyroid along <100> and the corresponding reciprocal lattice:

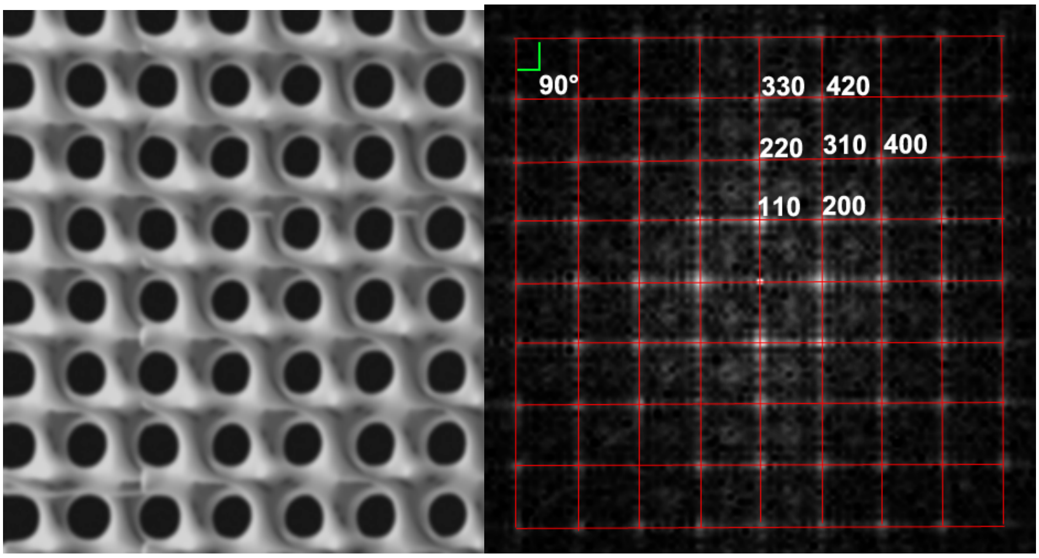

d_{110}=243.24 nm; angle between (110) planes = 90°

Figure S35. *Left: Projection image of simulated 3D structure along the <100> direction. Note that the holes or tunnels that run through the single gyroid crystal appear as black circles arranged in a square array with 90° angles between the rows and columns of tunnels. Right: Fourier analysis of the simulated projection showing the reciprocal lattice. Note that the reciprocal lattice is also a square grid.*

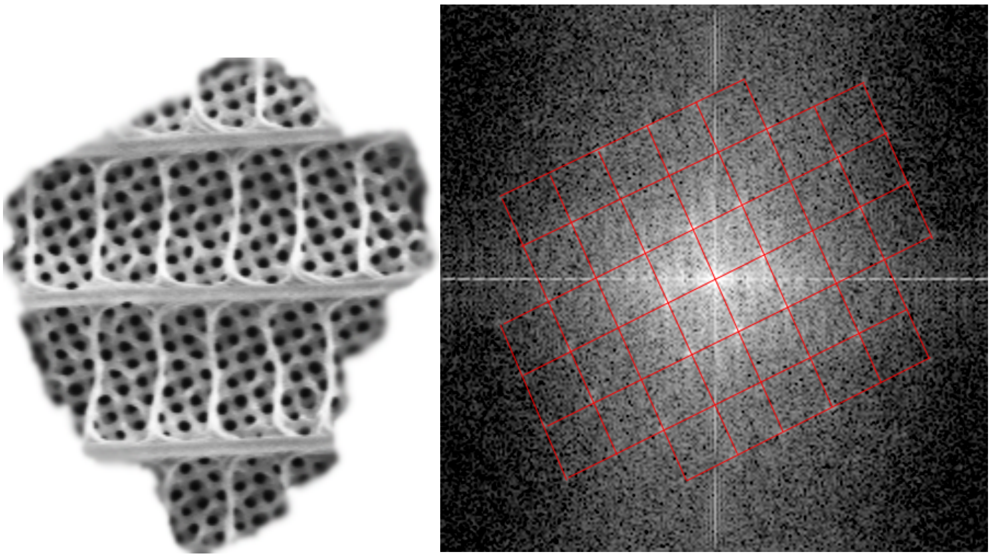

d_{110}=246.36; 242.04; 237.38; 246.36 nm; d_{200}=167.85; 169.55; 172.37; 172.66 nm

angles: 91.7; 88.22; 92.45; 91.01; 92.36; 92.14; 92.70; 87.66; 87.30 °

Figure S36 *Left: SEM image of an actual domain in Scale 1 aligned along the <100> direction. Right: Fourier analysis of the SEM image showing the reciprocal lattice.*

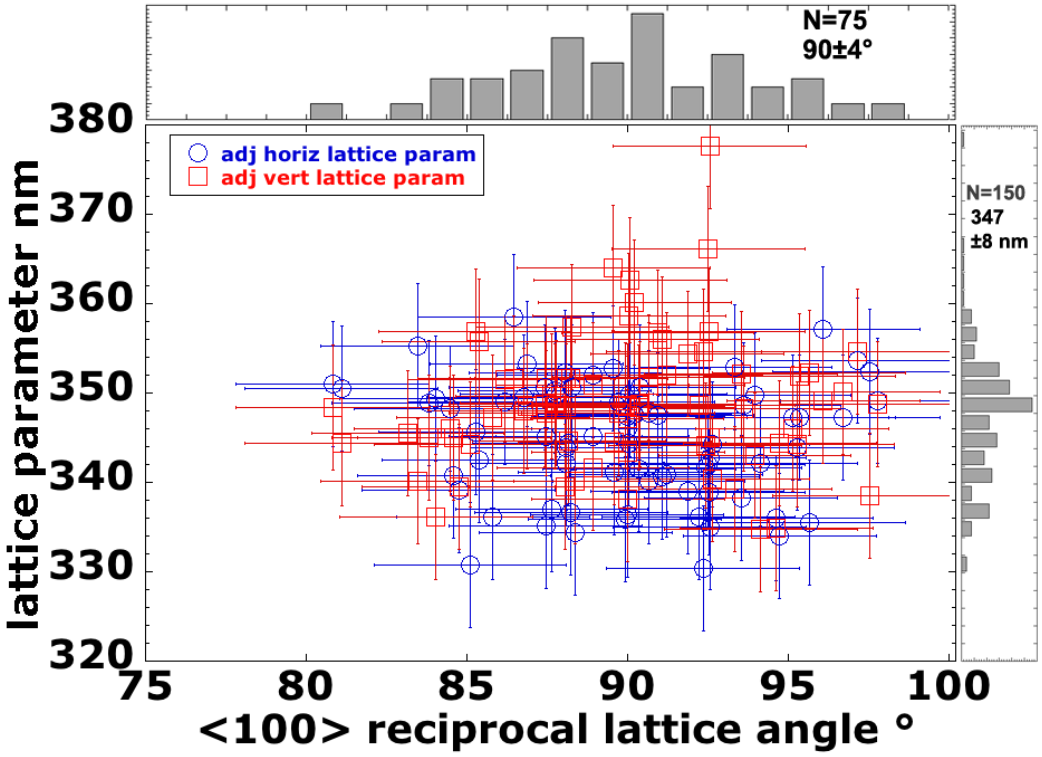

Figure S37. Lattice parameters and reciprocal lattice angle for 75 individual domains aligned along the <100> direction.

Assignment of <111> domains

Projection of a simulated single gyroid along <111> and the corresponding reciprocal lattice:

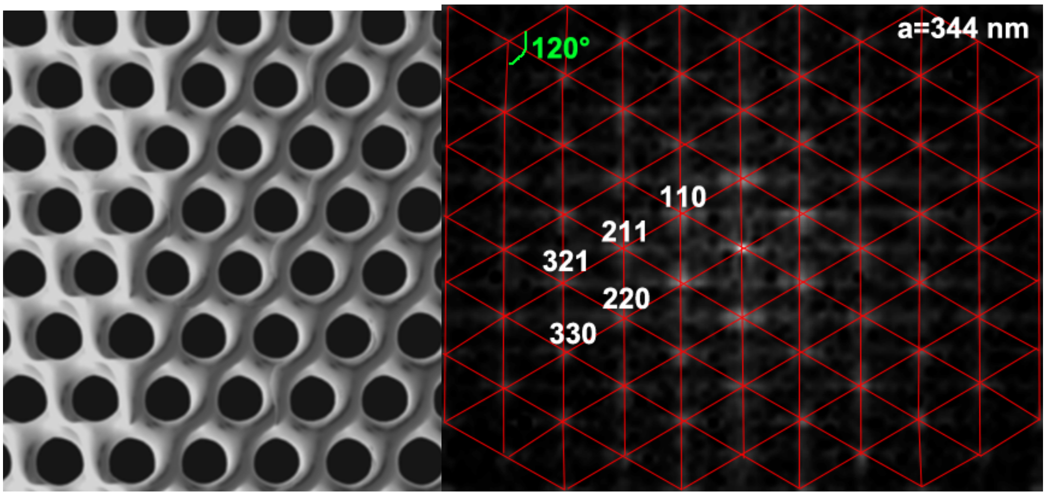

d_{110}=243.24 nm; angle between (110) planes = 120°

***Figure S38**. Left: Projection image of simulated 3D structure along the <111> direction. Right: Fourier analysis of the simulated projection showing the reciprocal lattice.*

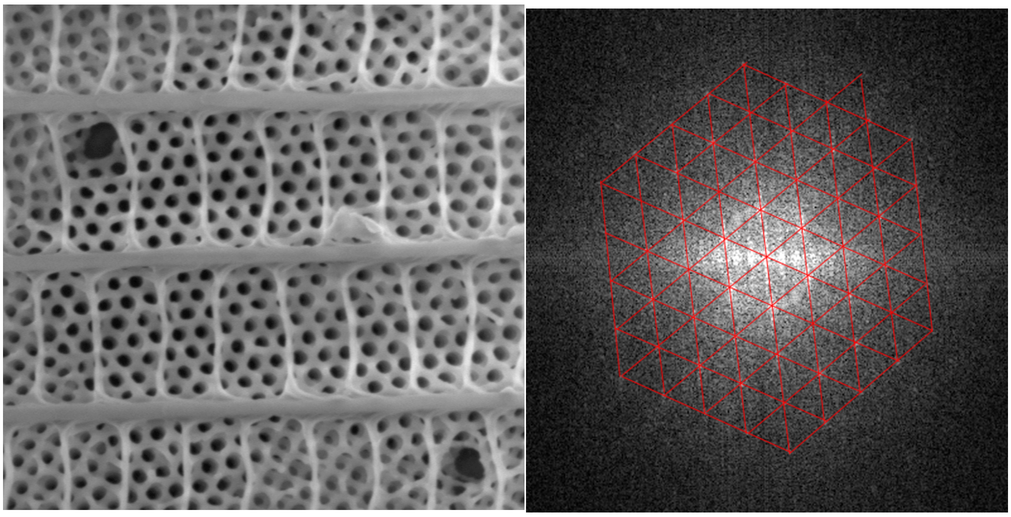

d_{110}=256.5; 243.6; 253.2; 259.9; 248.7; 257.5 nm;   angles=122.2; 123.6; 115.2; 116.3; 119.4; 122.5 °

***Figure S39**. Left: SEM image of an actual domain in Scale 1 aligned along the <111> direction. Right: Fourier analysis of the SEM image showing the reciprocal lattice.*

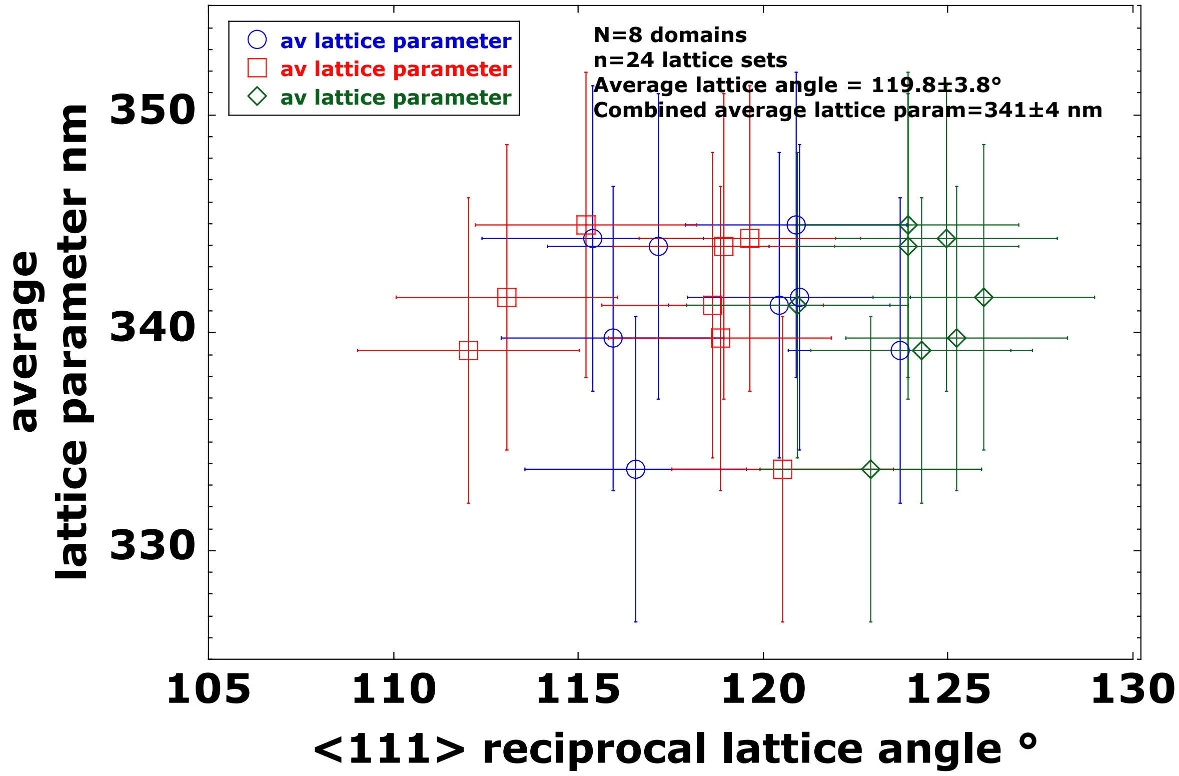

***Figure S40**. Data obtained from the Fourier transforms of individual domains assigned to the <111> orientation. Data from eight domains are included, each having three sets of lattice planes, thus 3x8=24 points. The error bars represent the uncertainty associated with the pixel resolution of the Fourier transforms. The spread is likely due to be a combination of image distortions associated with charging of the sample in the SEM electron beam and sample-specific phenomena such as a real spread in the distances and angles, surface distortions where the gyroids meet the ribs and cross ribs and tilting of the <111> axis.*

Assignment of <311> domains

Projection of a simulated single gyroid along <311> and corresponding reciprocal lattice:

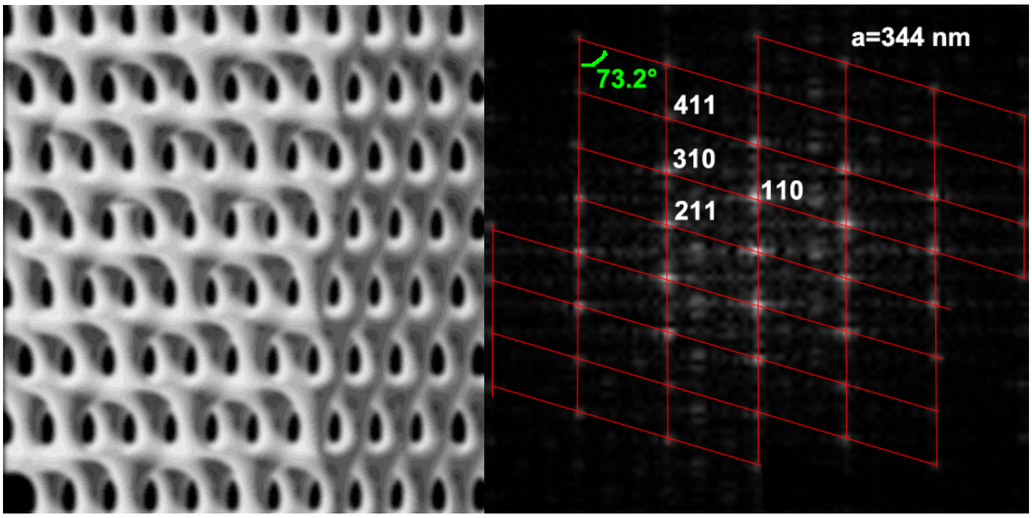

d110=243 nm; d211=140 nm; angle between (110) and (211) planes = 73.2°

Figure S41. *Left: Projection image of simulated 3D structure along the <311> direction. Right: Fourier analysis of the simulated projection showing the reciprocal lattice.*

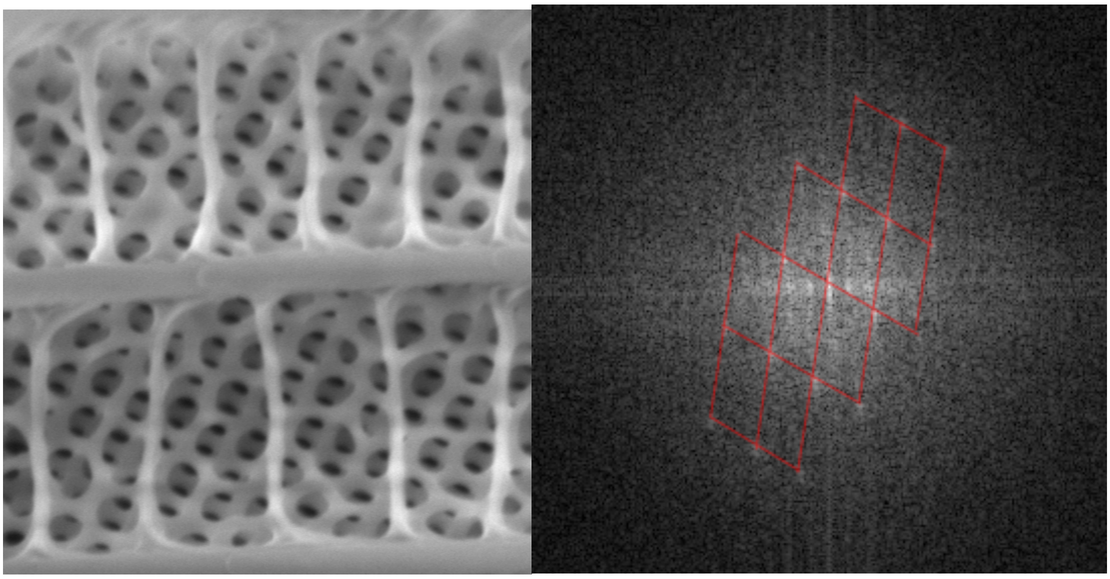

d_{110}=244.0; 248.1; nm d_{211}=144.7; 137.7; 134.0; 144.7 nm angles: 68.48; 69.28; 69.40; 68.73°

Figure S42. *First example. Left: SEM image of domain assigned to <311> from scale 1. Right: Corresponding reciprocal lattice.*

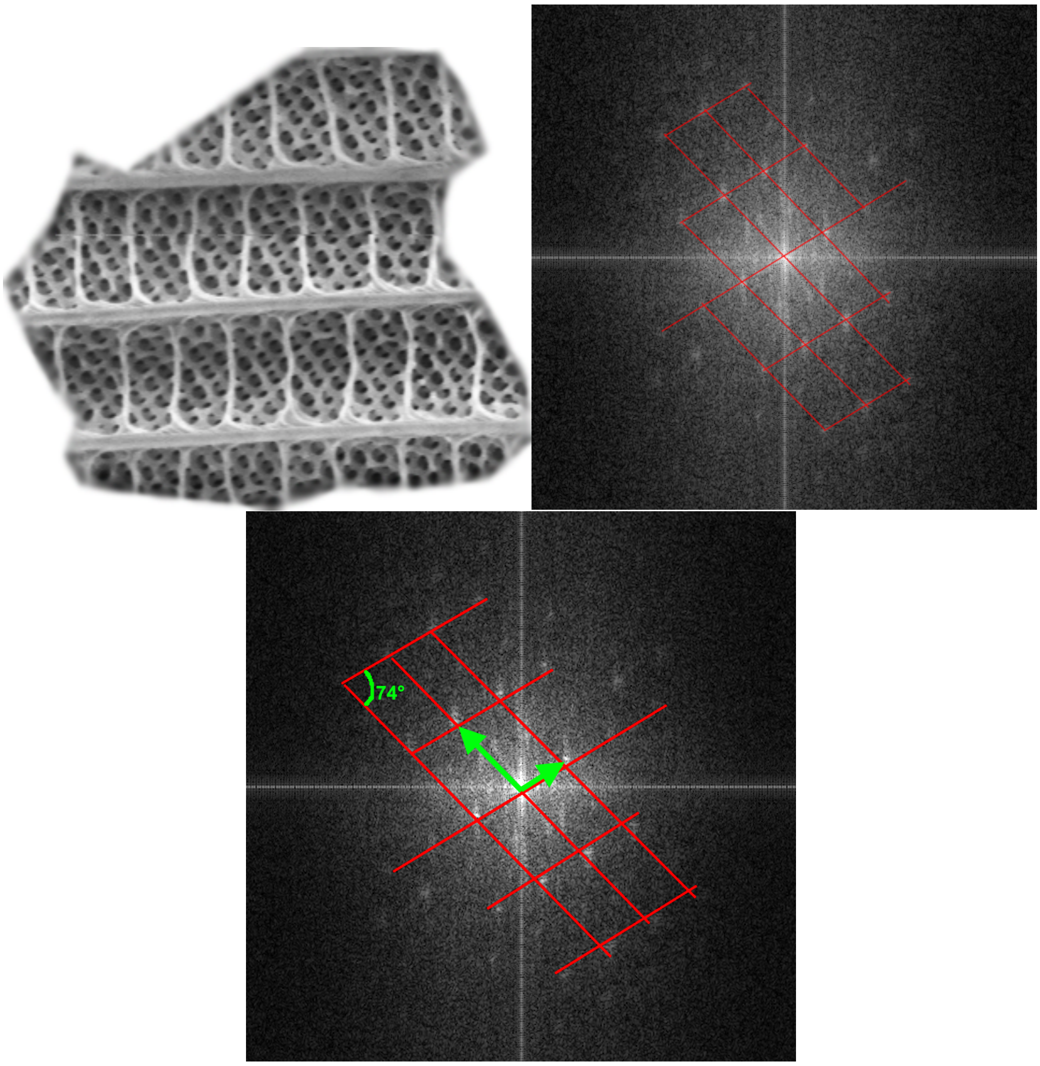

d_{110}=243.6; 245.26; 246.6; 242.9 nm; d_{211}=133.7; 136.9;134.7; 136.3 nm; angles: 77.5; 76.6; 77.5; 76.8 °

Figure S43*. Second example. Left: SEM image of domain assigned to <311> from scale 1. Right and bottom: Corresponding reciprocal lattice.*

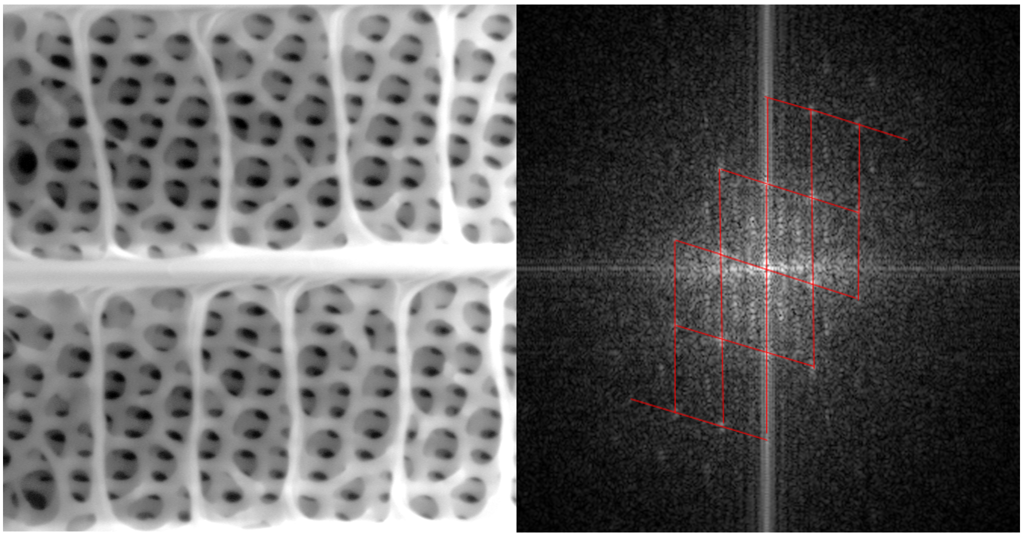

d_{110}=238.6; 234.5 d_{211}=130.9; 129.4; 133.0; 134.3 nm angles: 72.2; 73.8; 73.9; 73.4 °

Figure S44*. Third example. Left: SEM image of domain assigned to <311> from scale 1. Right and bottom: Corresponding reciprocal lattice.*

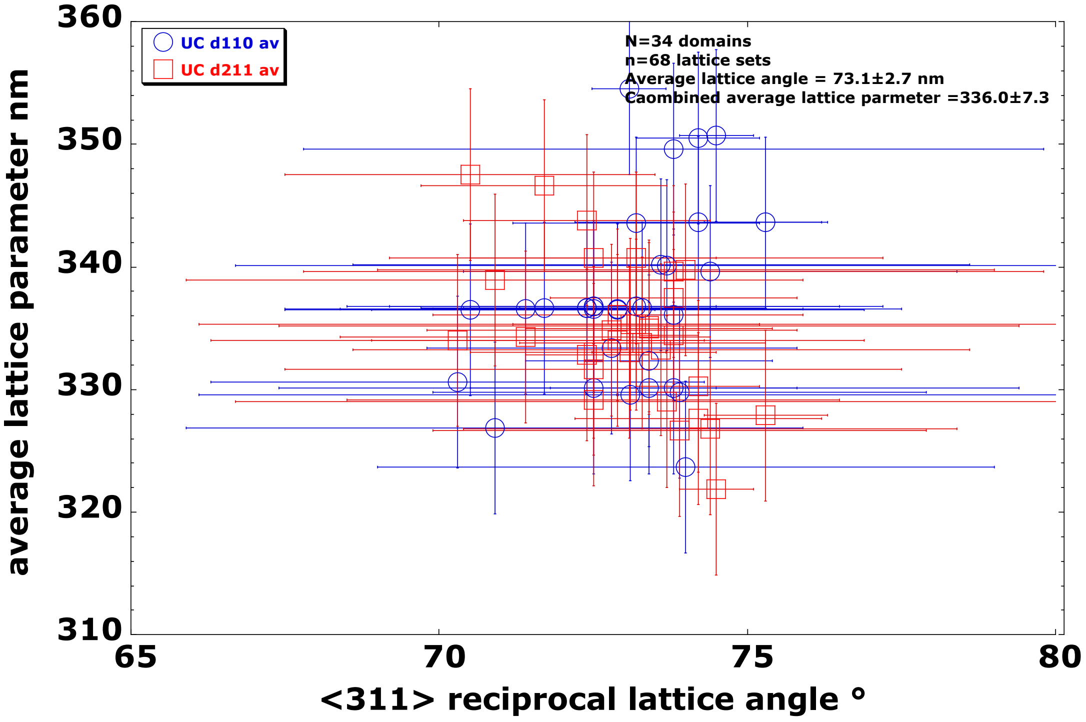

Figure S45. *Data obtained from the Fourier transforms of individual domains assigned to the <311> orientation. Data from 34 domains are included, each having two sets of lattice planes, thus 2x34=68 points. The error bars in the lattice parameter spacing represent the uncertainty associated with the pixel resolution of the Fourier transforms. The error bars in the angles represent the standard deviation of four measurements per domain.*

Assignment of <110> domains

Now the <110> has an rectangular plane lattice. The simulation looks quite different to that of the projection on <311> (also compare the SEM images below with Figure 5 of Yoshioka *et al.* (2014)[2]:

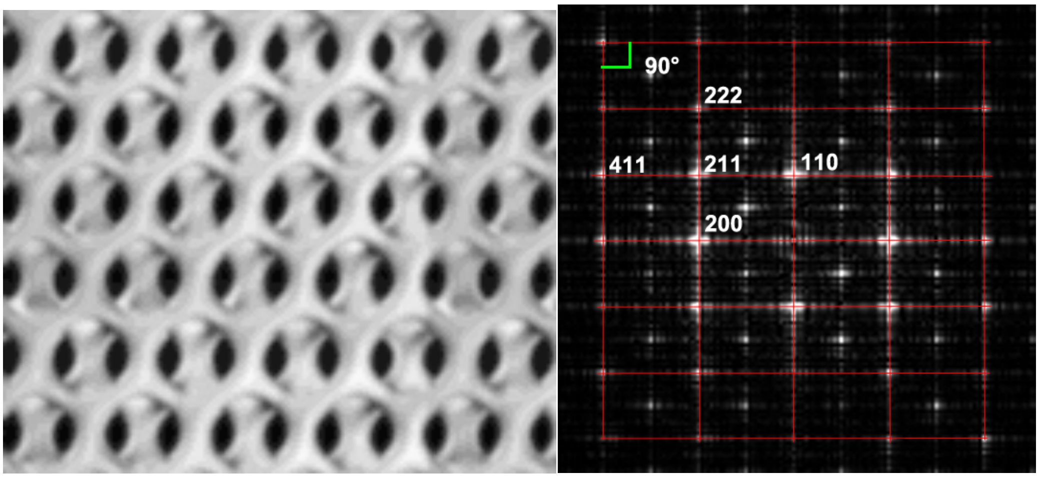

d_{110}=243 nm d_{200}=172 nm angle between (110) and (200) planes = 90°

Figure S46. *Left: Projection image of simulated 3D structure along the <110> direction. Right: Fourier analysis of the simulated projection showing the reciprocal lattice.*

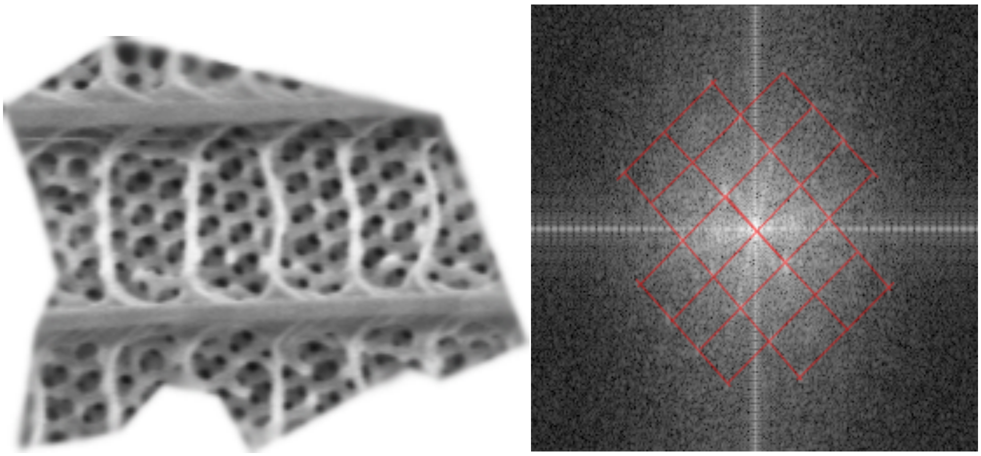

d_{110} =243.5; 244.2 nm d_{200}=177.2;177.2 nm angles: 85.5; 94.0; 86.6; 93.1°

Figure S47. *First example. Left: SEM image of domain assigned to <110> from scale 1. Right: Corresponding reciprocal lattice.*

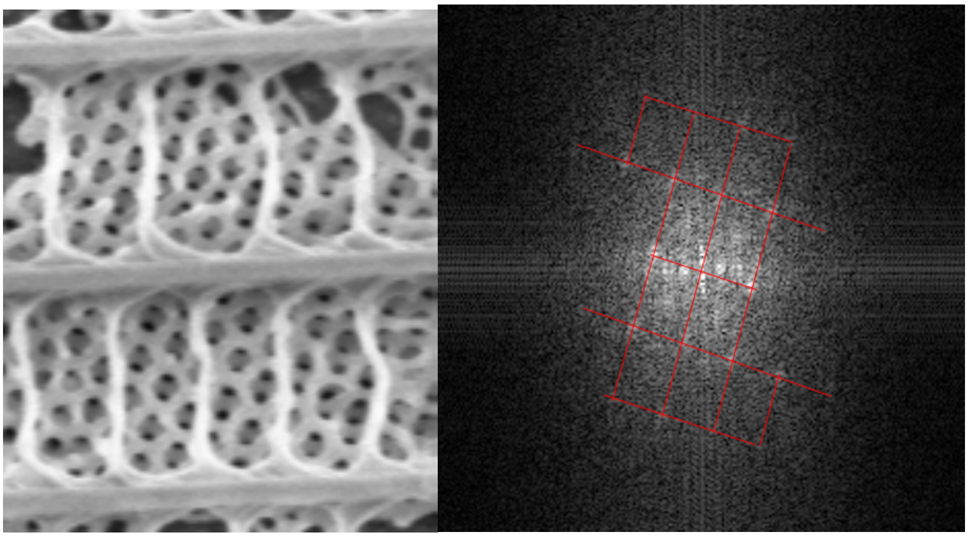

d_{110}=244.5; 247.6 nm d_{200}=165.8; 170.5 nm angles = 92.5; 88.1; 89.3; 91.5 °

Figure S48. *Second example. Left: SEM image of domain assigned to <110> from scale 1. Right: Corresponding reciprocal lattice.*

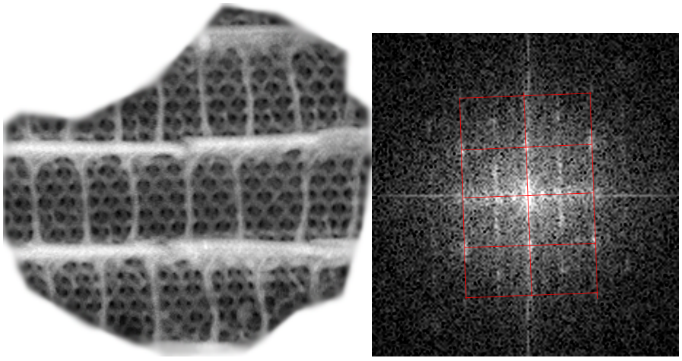

d_{110}=242.9; 242.9; 242.4; 247.8 nm d_{200}=175.1; 174.8 nm angles = 90.7; 90.0; 90.9; 89.6 °

Figure S49. *Third example. Left: SEM image of domain assigned to <110> from scale 1. Right: Corresponding reciprocal lattice.*

Multi-domain statistical study of <311> oriented domains

Given that a significant percentage of domains were found to align along or near the <311> axis, which is not a major crystal axis, a more thorough analysis was undertaken to check the assignments were consistent with projections along this particular axis.

The figure below shows a mosaic of all domains assigned to be aligned within 5° of the <311> and thus deemed "on-axis". Each domain has been rotated to align the projected (110) planes with the horizontal axis of the image. Note that rotating each domain in this way randomizes the alignment of the ribs and cross ribs and allows a more intuitive visualization of the in-plane or azimuthal randomness. Fourier transformation of this image therefore gives an effective average single crystal pattern in reciprocal space that is then readily compared against the theoretical prediction as another check of the quality of the assignments.

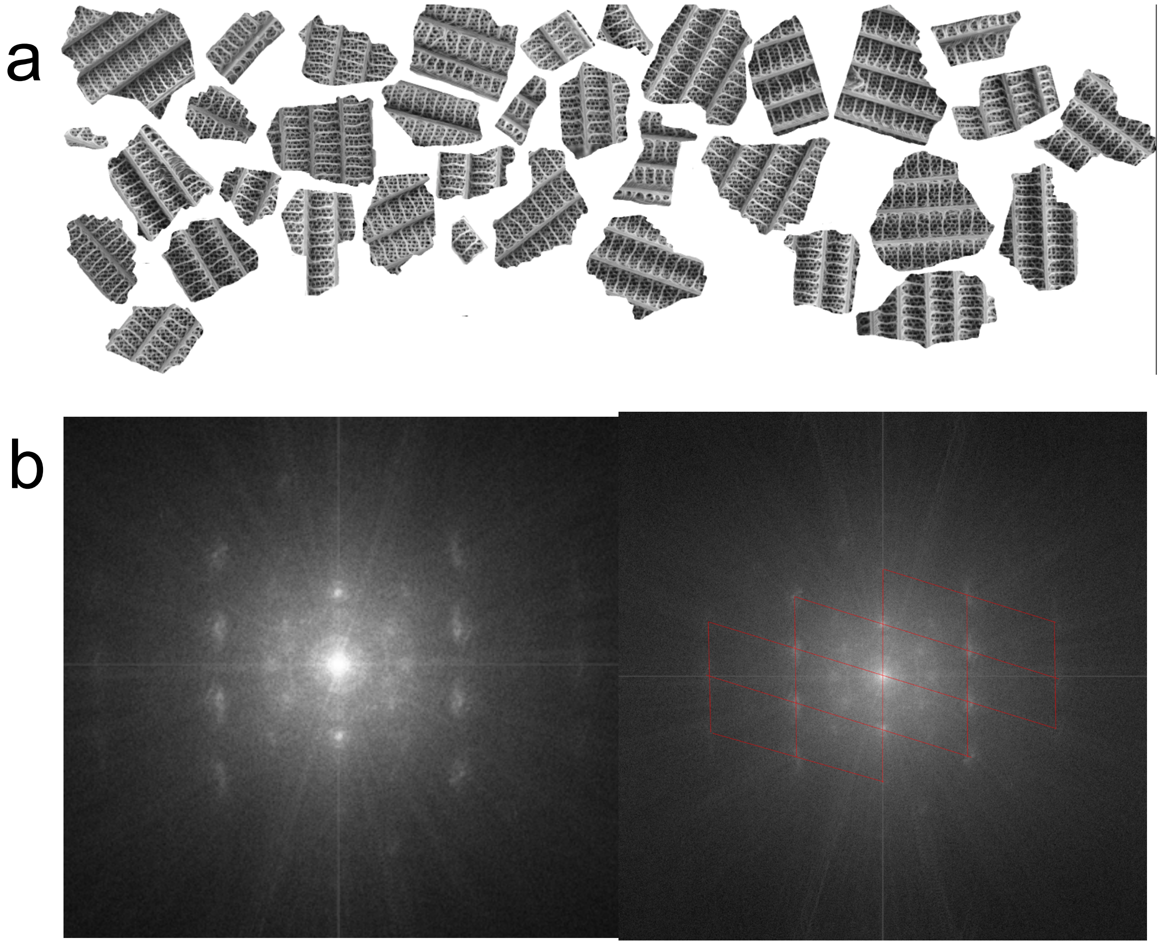

d_{110}=235,78; 238.14; Average = 236.96 nm d_{220}=120.0; 120.0; Average = 120°

d_{210}=135.73; 136.63;136.89; 138.32; Av.=136.89 nm d_{310}=102.28; 107.1; 106.0; 102.5; Average=104.47

angles: 71.94; 72.14; 72.58; 73.71; 72.75; 72.68; 72.94; Average = 72.26°

***Figure S50**. a) Mosaic of all domains in wing scale 1 assigned to be aligned within 5° of the <311> and thus deemed "on-axis". Each domain has been rotated to align the projected (110) planes with the horizontal axis of the image. b) Corresponding Fourier transform of the mosaic image is therefore valuable in returning the characteristic pattern of an average single crystal with well-defined point-like peaks. Note that the ribs and cross ribs appear as circular rings in the frequency domain with average spacings of: 1940nm; 1903; 1867 and 1794 nm for ribs and 645, 666, 721, 695; 666; 699; 681; 654 nm.*

This analysis was extended to the 56 domains from wing scale 1 classified as “off-axis” <311>. Below is the figure of the rotated domains.

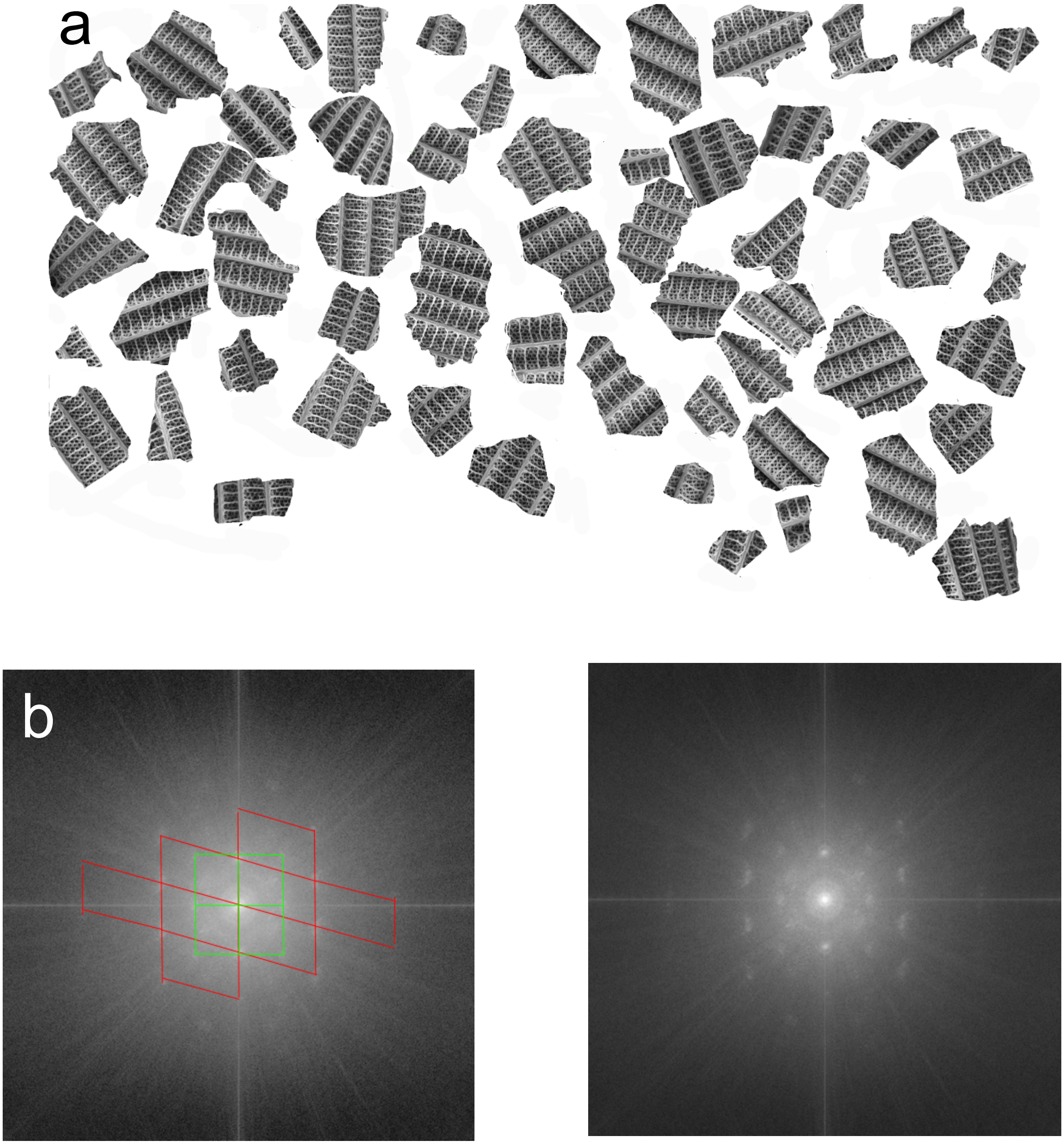

d110=235.74; 234.55; d211=137.42; 138.17; 139.67; 138.78

Figure S51. *a) Mosaic of all domains assigned to be “off-axis” <311>. Each domain was rotated in real space to align the projected (110) planes with the horizontal axis of the image prior to Fourier transformation. b) Corresponding Fourier transforms which are again highly similar to that of the simulated structure projected along <311>.*

The main power in the spots is associated with the intersecting (110) and (211) planes that define the plane reciprocal space lattice shown with red lines. However there is also secondary power associated with spots closer to the origin and can be seen to form another lattice shown in green (corresponding to (200) planes), and other spots are also seen but not indicated with colours. These secondary spots arise partly from the fact that the projected images contain textures related to the 3D structure, and appear to be sensitive to the degree of tilt away from the <311> axis. This is discussed further below.

The comparison of the on and off axis single crystal patterns shows that the spots associated with the (211) planes are more smeared out in the off-axis compared to the more concentrated corresponding spots in the FFT from the on-axis domains. Given that the vertical spots from the (110) planes form a spherically symmetric tight point, we cannot assign the smearing to inaccuracies in rotating each of the real space images prior to Fourier transformation.

Finally, radially integrated FFT data from <311> domains shown in Figure S10 is decomposed into the on- and off-axis contributions below. The primary difference is the intensity of the $\sqrt{4}$ peak, which appears more intense in the "off-axis" domains. As will be seen in the next sub-section, this is consistent with more tilt towards the <100>, where these spots become relatively more intense.

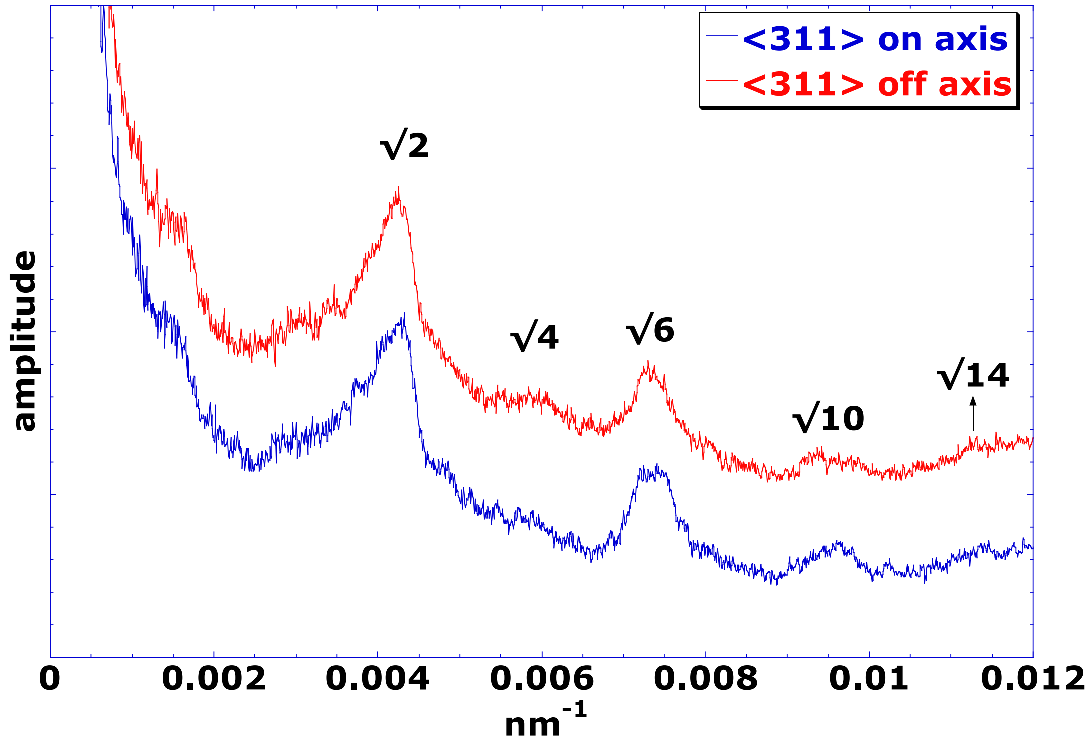

Figure S52*. Radially integrated FFT data from <311> domains from wing scale 1, decomposed into the on- and off-axis contributions.*

FFT series as a function of rotating the single gyroid about a <110> axis

The simulated projections below were adjusted by inspection so that the lighting closely approximated the effective “lighting” seen in the SEM images, in particular to match the level of illumination at depth into the structure. The structures in this series of simulations were clipped so that plane closest to the viewer was as planar as possible (as it is in the real butterfly scales flat). In this case, the surface of a large radius sphere was used as the clipping “plane”.

Here the sequence starts with a projection down <100> (0° rotation) with subsequent projections in 5° steps out to 10° beyond <311> (≈25° rotation) to the <211> (≈35° rotation). So below we see 0-35° from 100. The observed data sets are shown in their respective positions of estimated best fit.

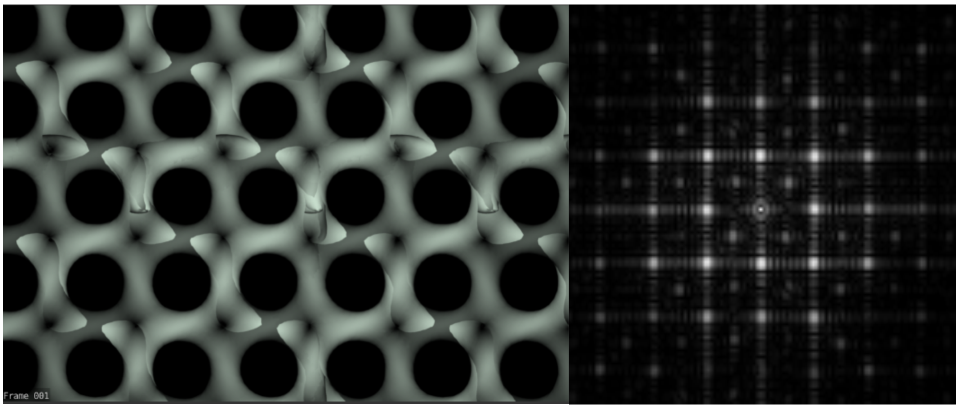

Figure S53. *Simulated projection and corresponding Fourier transform aligned along the <100> axis. 0° tilt*

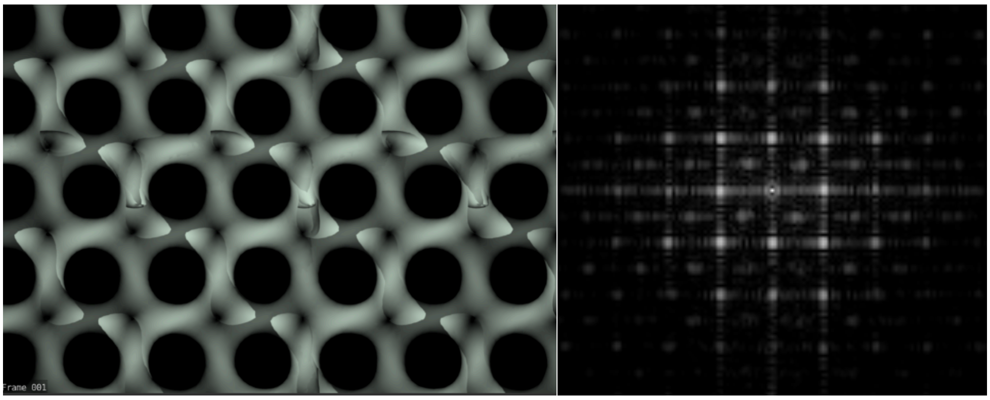

Figure S54. *Simulated projection and corresponding Fourier transform aligned along the <100> axis. 5° tilt*

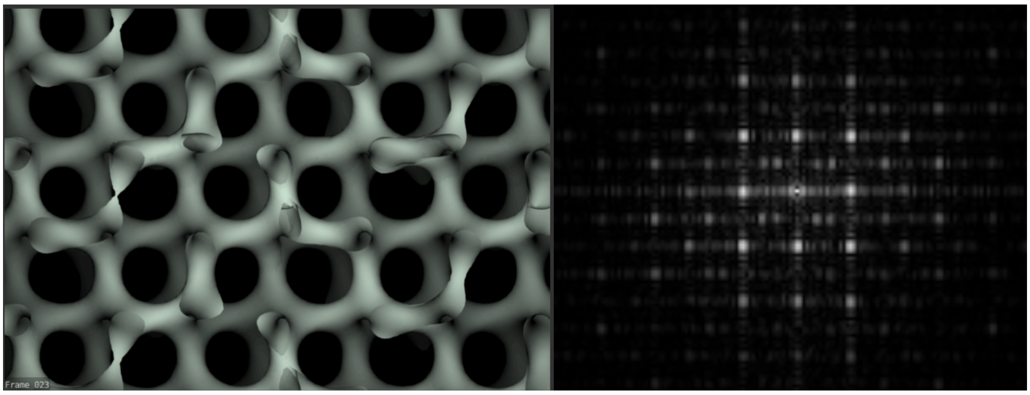

Figure S55. *Simulated projection and corresponding Fourier transform aligned along the <100> axis. 10° tilt*

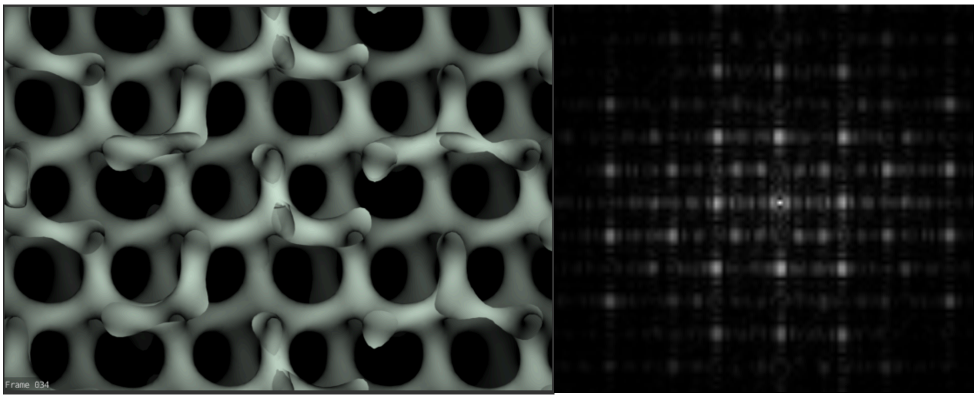

Figure S56. *Simulated projection and corresponding Fourier transform aligned along the <100> axis. 15° tilt*

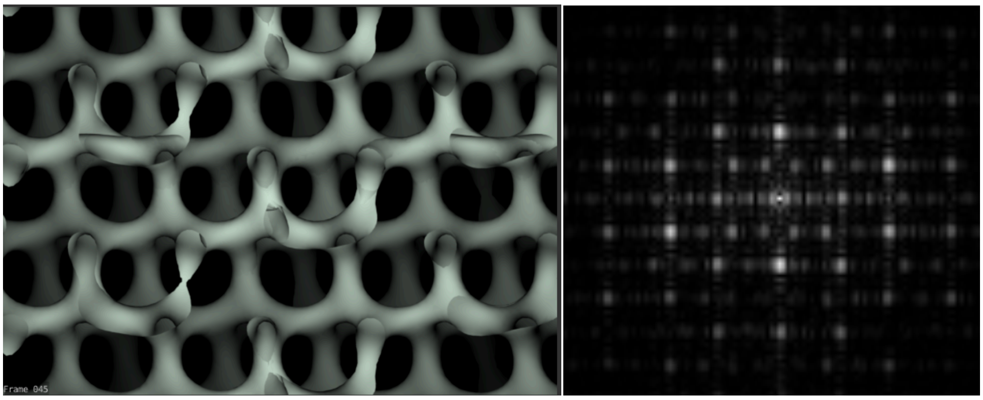

Figure S57. *Simulated projection and corresponding Fourier transform aligned along the <100> axis. 20° tilt.*
Note that 25° to 35° tilt projections are shown in Figures S60 to S62.

FFTs as a function of tilt while rotating from <311> to <110> 5° per step, shows that the pattern most resembling the average scattering from the <311> domains is 5° tilted from <311> towards <100> around the <110> axis.

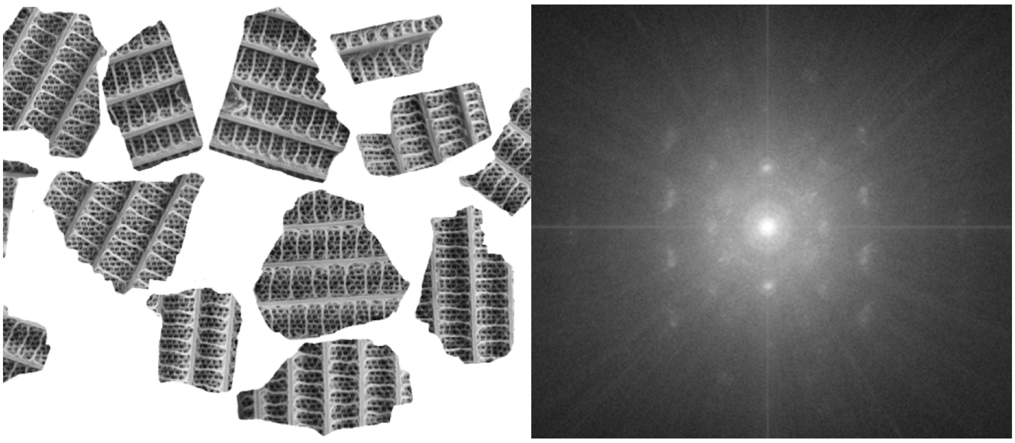

Figure S58. *Observed "off axis" <311> domains from wing scale 1 and corresponding Fourier transform.*

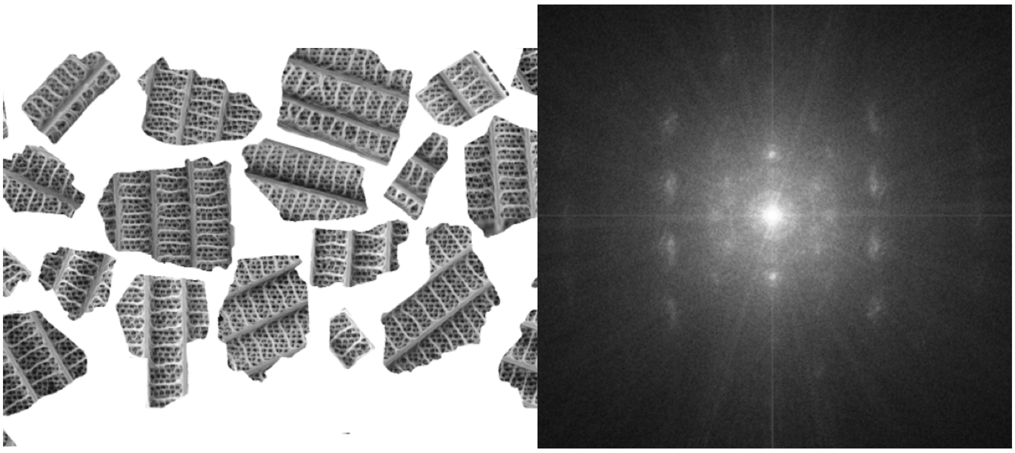

Figure S59. *Observed "on axis" <311> domains from wing scale 1 and corresponding Fourier transform.*

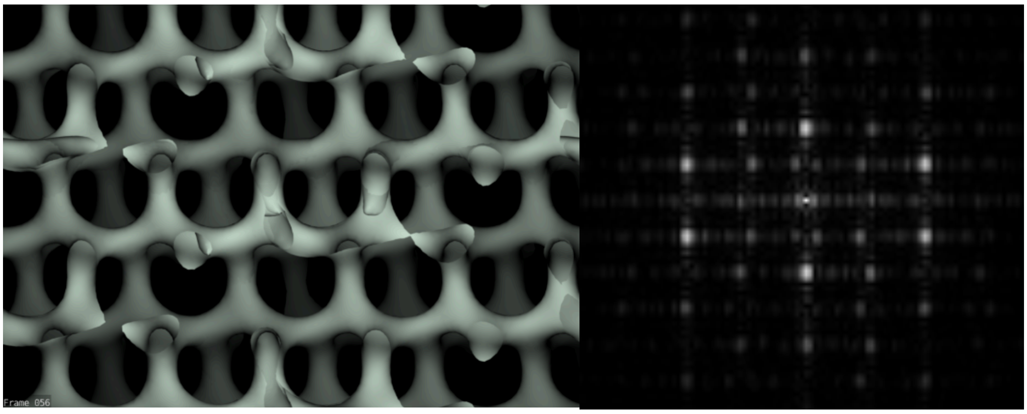

Figure S60. *Simulated projection and corresponding Fourier transform aligned along the <311> axis. 0° tilt (or 25.24° tilt from the <100> axis)*

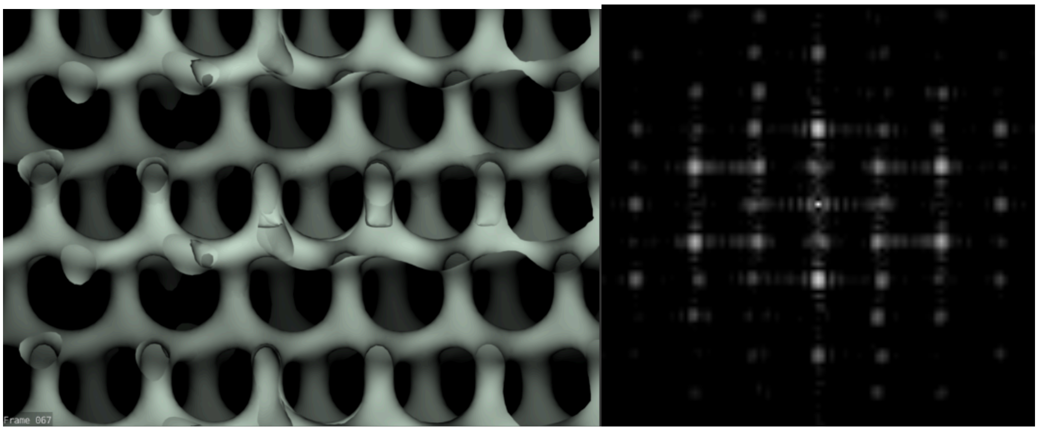

Figure S61. *Simulated projection and corresponding Fourier transform aligned along the <311> axis. 4.76° tilt (or 30° tilt from the <100> axis)*

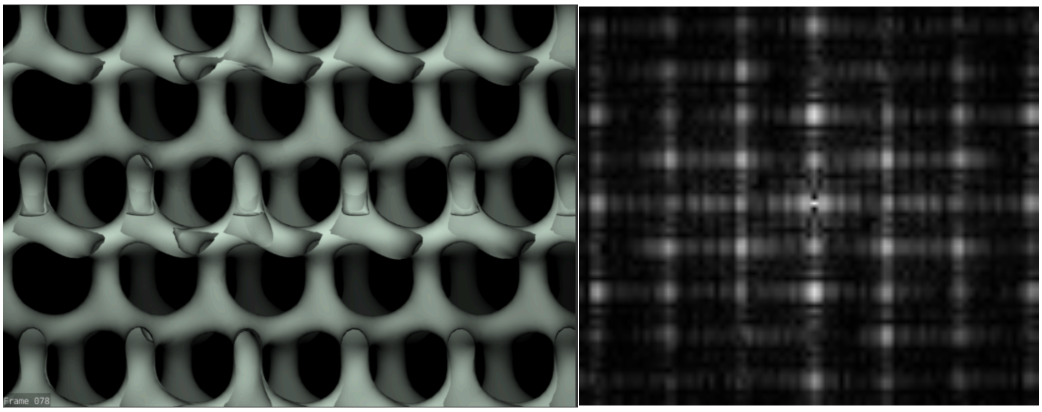

Figure S62. *Simulated projection and corresponding Fourier transform aligned along the <311> axis. 9.76° tilt (or 35° tilt from the <100> axis)*

Figure S63 below shows 5 and 10° displacements of the normal vector by rotation around an arbitrary low symmetry axis orthogonal to some <110> axis, and away from the <311> axis:

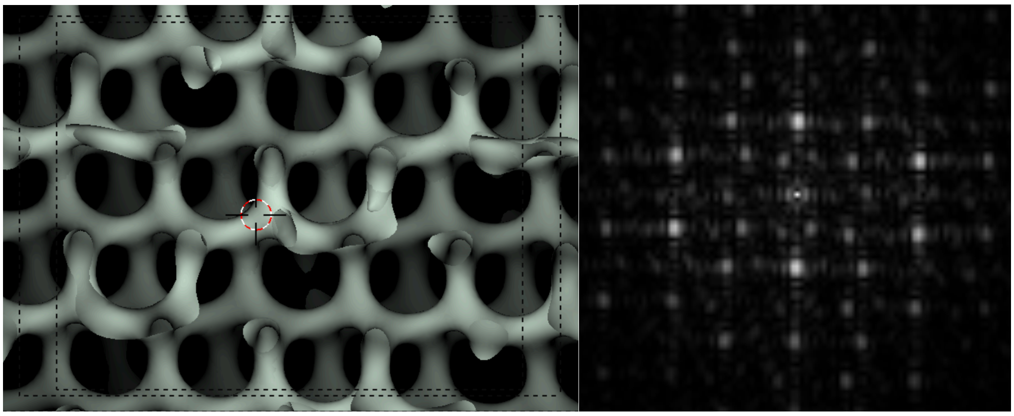

5° away from <311>

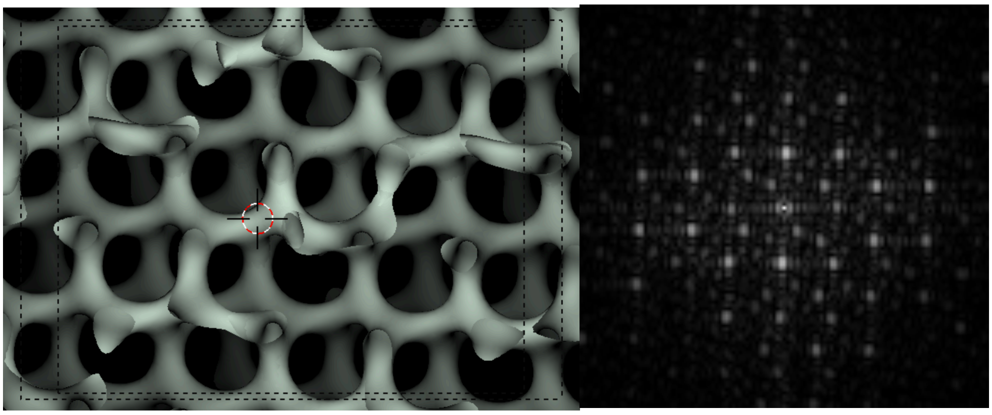

10° away from <311>

Figure S63. *Simulated projection and corresponding Fourier transform aligned with an arbitrary 5° and 10° angular displacement away from the <311> axis . Note the immediate break in horizontal and vertical mirror symmetries. This degree of symmetry breaking does not appear in the Fourier transform of the observed <311> domains, thus it is likely that the spread of orientations in <311> oriented crystals is likely by rotation around either an axis that preserves these mirror symmetries, i.e., either a <110> or <100> axis.*

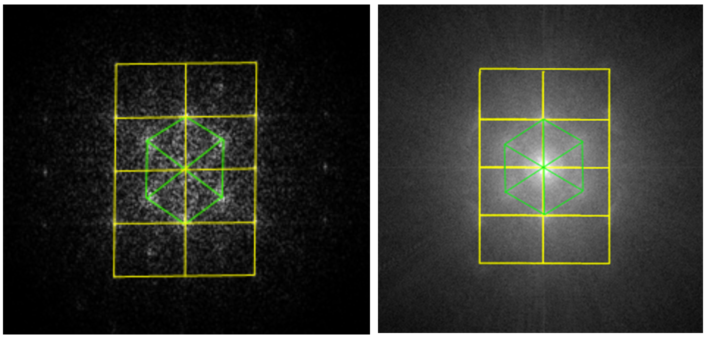

Figure S64. *Left: Fourier transform of the bottom right <110> oriented domain in Figure 5 of Yoshioka et al. (2014) NB it is rotated by 90° relative to the original published image. Compare this with Figures S46-S49. Right: Average single crystal pattern for rotated domains of on-axis <110> oriented crystals found here (same as Figure 3g in the main text). Note that the well-aligned <110> domain also produces a higher order pseudo-hexagonal set of spots characteristic of this orientation.*

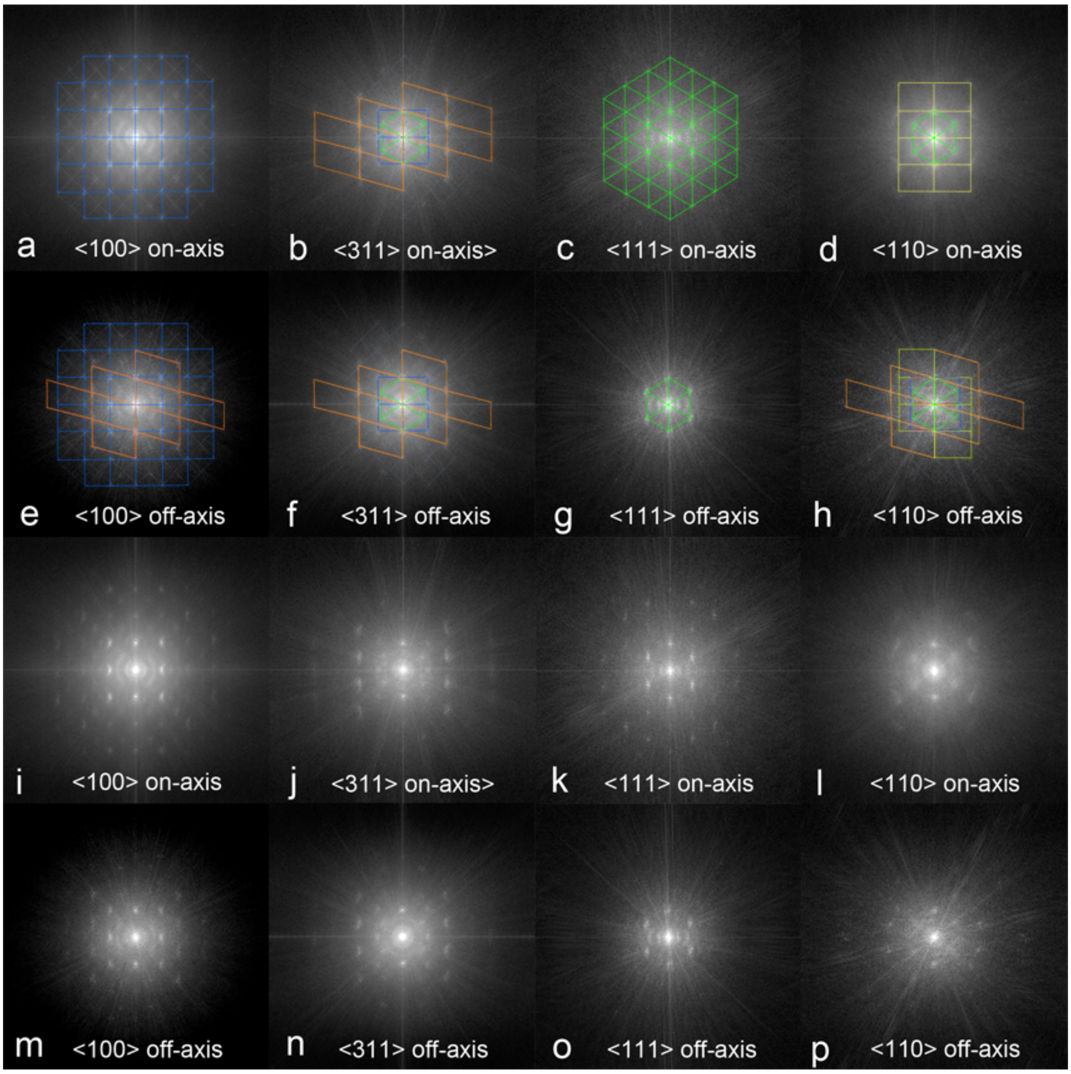

Figure S65. *'Stacked' FFTs corresponding to all on- and off-axis domains from wing scale 1. Compare with Figure 3 of the main text. Here the coloured grids have been removed in the lower half of the figure to show the raw FFT peaks.*

Section 13. Notation for describing crystallographic axes and planes

The following notations were used in this article to:

(hkl) is the Miller index of a specific crystal plane belonging to the general set of {hkl} crystal planes.

For example the specific crystal planes {011}, {$\bar{1}$01} and {110} are three of the 12 members belonging to the set of general set of (110) crystal planes.

[hkl] is the Miller index of a specific crystal axis belonging to the general set of <hkl> crystal axes.

For example the specific crystal axes [311], [$\bar{1}$31] and [113] are three of the 24 members belonging to the set of general set of <311> crystal planes.

As a further note, a direction normal to a specific crystal plane (hkl) shares the same Miller indices, i.e. [hkl].

For example, the direction [010] is normal to the (010) plane.

Section 14. References

1 von Schnering, H. G. & Nesper, R. Nodal surfaces of Fourier series: Fundamental invariants of structured matter. *Z. Physik. B - Condensed Matter* **83**, 407-412 (1991).

2 Yoshioka, S., Fujita, H., Kinoshita, S. & Matsuhana, B. Alignment of crystal orientations of the multi-domain photonic crystals in *Parides sesostris* wing scales. *Journal of The Royal Society Interface* **11**, 20131029 (2014).
